# A CRISPR/Cas9 toolkit for multiplex genome editing in plants

**DOI:** 10.1186/s12870-014-0327-y

**Published:** 2014-11-29

**Authors:** Hui-Li Xing, Li Dong, Zhi-Ping Wang, Hai-Yan Zhang, Chun-Yan Han, Bing Liu, Xue-Chen Wang, Qi-Jun Chen

**Affiliations:** State Key Laboratory of Plant Physiology and Biochemistry, College of Biological Sciences, China Agricultural University, Beijing, 100193 China

**Keywords:** CRISPR/Cas9, Genome editing, Multiple gene mutations, Assembly of multiple gRNAs

## Abstract

**Background:**

To accelerate the application of the CRISPR/Cas9 (clustered regularly interspaced short palindromic repeats/ CRISPR-associated protein 9) system to a variety of plant species, a toolkit with additional plant selectable markers, more gRNA modules, and easier methods for the assembly of one or more gRNA expression cassettes is required.

**Results:**

We developed a CRISPR/Cas9 binary vector set based on the pGreen or pCAMBIA backbone, as well as a gRNA (guide RNA) module vector set, as a toolkit for multiplex genome editing in plants. This toolkit requires no restriction enzymes besides *Bsa*I to generate final constructs harboring maize-codon optimized *Cas9* and one or more gRNAs with high efficiency in as little as one cloning step. The toolkit was validated using maize protoplasts, transgenic maize lines, and transgenic *Arabidopsis* lines and was shown to exhibit high efficiency and specificity. More importantly, using this toolkit, targeted mutations of three *Arabidopsis* genes were detected in transgenic seedlings of the T1 generation. Moreover, the multiple-gene mutations could be inherited by the next generation.

**Conclusions:**

We developed a toolkit that facilitates transient or stable expression of the CRISPR/Cas9 system in a variety of plant species, which will facilitate plant research, as it enables high efficiency generation of mutants bearing multiple gene mutations.

**Electronic supplementary material:**

The online version of this article (doi:10.1186/s12870-014-0327-y) contains supplementary material, which is available to authorized users.

## Background

Approaches for precise, efficient gene targeting or genome editing are highly important for functional genomic analysis of plants and for the production of genetically engineering crops. For the majority of researchers, transfer DNA (T-DNA) and transposon insertional mutagenesis remain the main sources of mutants of genes of interest in model plants such as the dicot *Arabidopsis thaliana* and the monocot rice (*Oryza sativa*) [1,2]. There is an increasing demand for plants bearing mutations in multiple genes in order to dissect the functions of gene family members with redundant functions and to analyze epistatic relationships in genetic pathways. However, the current method for generating plants carrying multiple mutated genes requires time-consuming and labor-intensive genetic crossing of single-mutant plants. Moreover, T-DNA insertional mutants cannot be obtained for every gene of interest. Therefore, new technologies that are affordable, efficient, and user-friendly are needed for plant genome targeting.

Double-strand breaks (DSBs) at specific genomic sites can introduce a mutation at the DNA break site via the error-prone non-homologous end-joining (NHEJ) pathway. DSBs can also result in homologous recombination (HR) between chromosomal DNA and foreign donor DNA through the HR pathway [3]. Based on DSBs at target loci, sequence-specific nucleases, including homing meganucleases, zinc finger nucleases, and transcription activator-like effector (TALE) nucleases have emerged as powerful technologies for targeted genome editing in eukaryotic organisms [3].

Recently, another DSB-based breakthrough technology for genome editing, the CRISPR/Cas system, was developed [4,5]. This system is based on the bacterial and archaeal clustered regularly interspaced short palindromic repeats (CRISPR) adaptive immune system for purging invading viral and plasmid DNA, which relies on the endonuclease activity of CRISPR-associated (Cas) proteins, with sequence specificity directed by CRISPR RNAs (crRNAs) [6-11]. The CRISPR/Cas system, which is employed in a variety of organisms, is derived from the *Streptococcus pyogenes* type II CRISPR system and consists of three genes, including one encoding Cas9 nuclease and two noncoding RNA genes: trans-activating crRNA (tracrRNA) and precursor crRNA (pre-crRNA). The programmable pre-crRNA, which contains nuclease guide sequences (spacers) interspaced by identical direct repeats, is processed to mature crRNA in combination with tracrRNA. The two RNA genes can be replaced by one RNA gene using an engineered single guide RNA (gRNA) containing a designed hairpin that mimics the crRNA–tracrRNA complex. The binding specificity of Cas9 with the target DNA is determined by both gRNA–DNA base pairing and a protospacer-adjacent motif (PAM, sequence: NGG) immediately downstream of the target region. Both nuclease domains of Cas9 (HNH and RuvC-like) cleave one strand of double-stranded DNA at the same site (three-nucleotide [nt] distance from the PAM), resulting in a DSB [8-11]. The CRISPR/Cas system has been harnessed to achieve efficient genome editing in a variety of organisms, including bacteria, yeast, plants, and animals, as well as human cell lines [12-27]. More importantly, using this RNA-guided endonuclease technology, multiple gene mutations and their germline transmission have been achieved [28-30].

In vertebrates such as zebrafish, mice, rats, and monkeys, coinjection of gRNA and Cas9-encoding mRNA transcribed in vitro into single-cell-stage embryos can efficiently generate animals with multiple biallelic mutations that can be transmitted to the next generation with high efficiency [18,28-32]. However, this method is not feasible in plants, where transgenic lines stably expressing the CRISPR/Cas9 system are required for the generation of plants with one or more gene mutations. *Agrobacterium*-mediated transformation is a routine method used to generate transgenic plants, and a few binary vectors have been developed to deliver the CRISPR/Cas9 system into plant genomes via this method [15,20,23,24,33-40]. Nevertheless, to accelerate the application of this system to a variety of plant species under normal or complex conditions (such as targeted mutation of genes in the background of T-DNA insertional mutants), a toolkit with additional plant selectable markers, more gRNA modules, and easier methods for assembling one or more gRNA expression cassettes is frequently required, especially for targeted mutation of multiple genes. We report the development of such a toolkit for multiplex genome editing in plants.

## Results

### CRISPR/Cas9 binary vector set and gRNA module vector set for multiplex genome editing in plants

**Figure 1:**
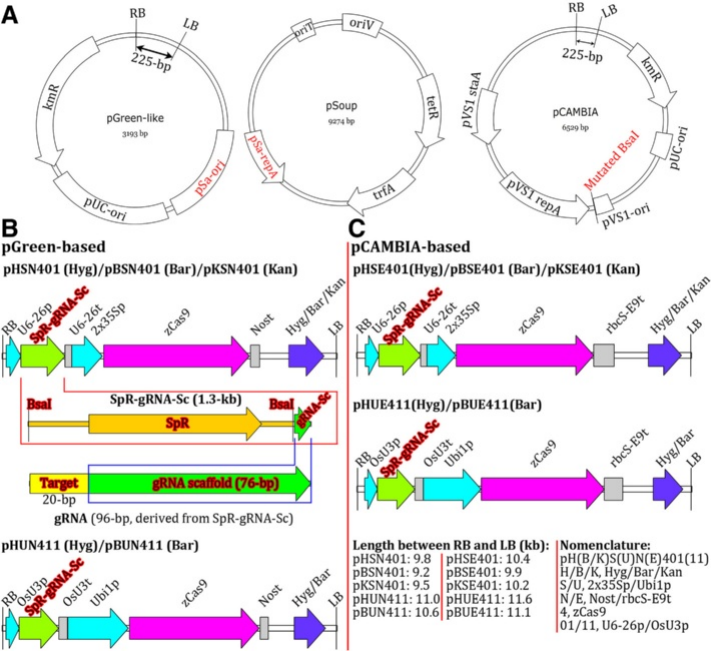
**Physical maps and structures of CRISPR/Cas9 binary vectors. (A)** Physical maps of the backbones of pGreen and pCAMBIA from which CRISPR/Cas9 binary vectors were derived. The map of the helper plasmid required for propagation of pGreen in *Agrobacterium* and the mutated *Bsa*I site on the pCAMBIA backbone are indicated. LB/RB, left/right border of T-DNA; pSa-ori, required for replication in *Agrobacterium* engineered with the corresponding replication protein (pSa-repA); KmR, kanamycin resistance gene; pUC-ori, replication origin required for replication in *E. coli*; pVS1-staA, pVS1-ori and pVS1-rep are the DNA elements required for replication in *Agrobacterium*. Only the 225-bp fragment between the LB and RB was left for comparison of the sizes of the pGreen and pCAMBIA backbones. **(B, C)** Physical maps of the regions between the RB and LB. The sizes of T-DNA regions and the structures of SpR-gRNA-Sc and final working gRNA are indicated. *zCas9*, *Zea mays* codon-optimized *Cas9*; U6-26p, *Arabidopsis U6* gene promoter; U6-26t, *U6-26* terminator with downstream sequence; OsU3p, rice *U3* promoter; OsU3t, rice *U3* terminator with downstream sequence; SpR, spectinomycin resistance gene; gRNA-Sc, gRNA scaffold.

Binary vectors with two types of backbones were utilized; one type is based on pGreen, while the other is based on pCAMBIA. The pGreen binary vectors were constructed based on a previously reported strategy [41]. The advantage of pGreen-like vectors is their relatively small size, allowing them to be used for transient Cas9 and gRNA expression in protoplasts to test the effectiveness of target sites. As the vectors can be directly used to generate transgenic plants after validation in protoplasts, the use of this single vector-based strategy for both transient and stable expression of CRISPR/Cas9 can save time, effort and money. In *Agrobacterium*, the pGreen-like vectors depend on their pSa origin for propagation, and they require a helper plasmid to provide replication protein (RepA). *Agrobacterium* containing pSoup helper plasmid can be used as hosts for pGreen-like vectors [41]. Among the pCAMBIA-derived binary vectors, those with a hygromycin-resistance gene as a selectable marker were derived from pCAMBIA1300, while those with a kanamycin-resistance gene were derived from pCAMBIA2300, and those with a Basta-resistance gene were derived from pCAMBIA3300. The vectors pCAMBIA1300/2300/3300 and their derivatives (including the Gateway-compatible pMDC series) are some of the most widely used binary vectors for a variety of plant species [42,43], and some plant transformation protocols have been specifically optimized based on these vectors. Therefore, the generation of pCAMBIA-based CRISPR/Cas9 binary vectors enhances the compatibility of these vectors with some optimized plant transformation protocols and/or the habits or preferences of some researchers. An important improvement in each of the pCAMBIA-derived vectors is that the *Bsa*I site in the pVS1 region (which is required for plasmid propagation in *Agrobacterium*) was disrupted in order to enable the use of *Bsa*I sites to assemble gRNA expression cassettes (Figure 1).

**Figure 2:**
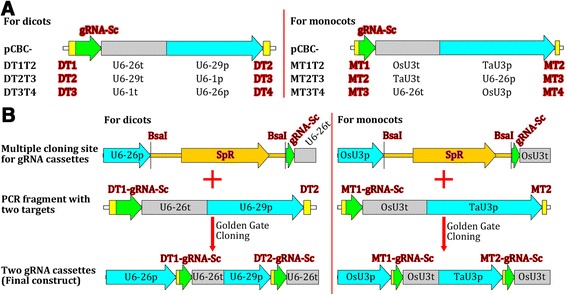
**Premade gRNA modules used for the assembly of two to four gRNA expression cassettes. (A)** gRNA-expressing modules for both dicots and monocots. U6-29p, U6-26p, and U6-1p are three *Arabidopsis U6* gene promoters; U6-29t, U6-26t, and U6-1t, corresponding *Arabidopsis U6* gene terminators with downstream sequences; OsU3p and TaU3p, rice and wheat *U3* promoters, respectively; OsU3t and TaU3t, rice and wheat *U3* terminators with downstream sequences, respectively; gRNA-Sc, gRNA scaffold; DT1/2/3/4, dicot target-1/2/3/4; MT1/2/3/4, monocot target-1/2/3/4. The vector pCBC is the cloning vector into which the gRNA modules were inserted separately. **(B)** Examples of the assembly of two-gRNA expression cassettes for dicots and monocots using the gRNA modules. Note: Each PCR fragment is flanked by two *Bsa*I sites (not shown).

In order to integrate multiple gRNAs into a single binary vector for multiplex genome editing, we constructed six gRNA module vectors, including three designed for dicots and three designed for monocots (Figure 2). Using these gRNA module vectors, two to more gRNA expression cassettes could easily be assembled using the Golden Gate cloning method [44,45] or the Gibson Assembly method [46]. By employing more suitable Pol III promoters, additional gRNA modules can be constructed for the assembly of more gRNA expression cassettes. Therefore, the gRNA module vector set is extensible and can easily be updated.

### Validation of the CRISPR/Cas9 toolkit in maize protoplasts

To validate the toolkit and to compare the mutation efficiency of different *Cas9* or Pol III promoters used to drive the gRNAs, we generated two sets of test vectors targeting the same maize genomic DNA site (*ZmHKT1*). One set comprises pBUN201-ZT1, pBUN301-ZT1, and pBUN401-ZT1, which harbor different *Cas9* sequences, including *hCas9-NLS-3 × FLAG* in pBUN201-ZT1, *3 × FLAG-NLS-hCas9-NLS* in pBUN301-ZT1 and *3 × FLAG-NLS-zCas9-NLS* in pBUN401-ZT1. The *hCas9* and *zCas9* sequences are human-codon and *Zea mays*-codon optimized *Cas9,* respectively. Another set comprises pBUN401-ZT1, pBUN411-ZT1, and pBUN421-ZT1. These vectors differ based on the Pol III promoters used to drive the gRNA: AtU6-26p in pBUN401-ZT1, OsU3p in pBUN411-ZT1 and TaU3p in pBUN421-ZT1.

**Figure 3:**
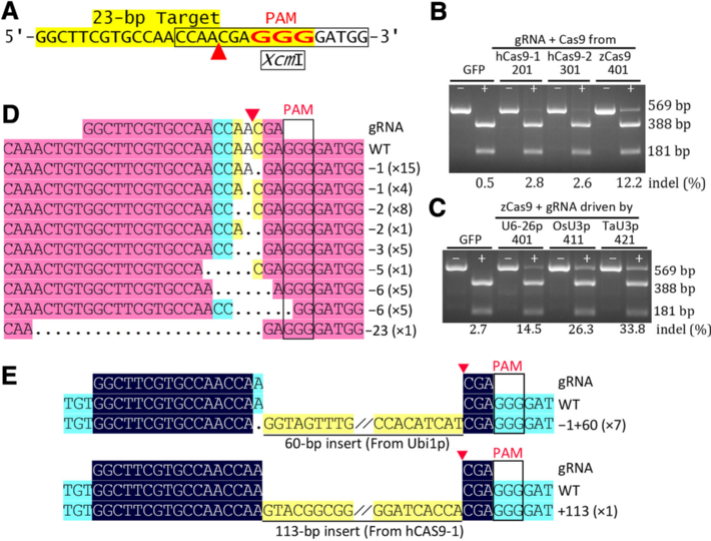
**Validation of maize codon-optimized Cas9 and three Pol-III promoters driving gRNA expression in maize protoplasts. (A)** Sequence of the target site from the Zm*HKT1* locus. The PAM, the putative cleavage site (red arrowhead), and the *Xcm*I site (boxed) are indicated. **(B,C)** Mutation analysis by *Xcm*I digestion of PCR fragments. GFP, 201, 301, 401 **(B)**: PCR fragments amplified from the genomic DNA of maize protoplasts transfected with pUC-GFP (control), pBUN201-ZT1, pBUN301-ZT1, and pBUN401-ZT1, respectively. The three CRISPR/Cas9 vectors have the same gRNA but different *Cas9*: *hCas9-1*/*2*, two types of human-codon-optimized *Cas9*; *zCas9*, *Zea mays* codon-optimized *Cas9*. GFP, 401, 411, 421 **(C)**: PCR fragments from the pUC-GFP, pBUN401-ZT1, pBUN411-ZT1, and pBUN421-ZT1 transfections, respectively; the three CRISPR/Cas9 vectors have the same z*Cas9* and gRNA, but the gRNA is driven by three different Pol-III promoters. − and + indicate whether the PCR fragments were digested with *Xcm*I. Mutation efficiency (% indel) calculated based on the percent ratios of residual undigested PCR fragments (+ lanes: 569 bp) to total PCR products (− lanes); the WT indel values should be treated as the background level. **(D,E)** Alignment of sequences of mutated alleles identified from cloned PCR fragments resistant to *Xcm*I digestion. The mutated alleles include deletions **(D)** and insertions **(E)**. Dots, deleted bases. Highlighting denotes the degree of homology of the aligned fragments, and only aligned regions of interest are shown. The type of indel and the number of indels of the same type are indicated.

For the target site *ZT1*, the mutated alleles were examined via *Xcm*I digestion of the PCR fragments surrounding the putative cleavage site (Figure 3A). *Xcm*I analysis indicated that maize codon-optimized *Cas9* performed considerably better than the two human codon-optimized *Cas9* genes (Figure 3B). The *TaU3* promoter appeared to perform slightly better than the *OsU3* promoter, and the *OsU3* promoter performed much better than the *AtU6-26* promoter (Figure 3C).

To verify mutation events, the PCR products were cloned, and the resulting colonies were screened by colony PCR and *Xcm*I digestion of the colony PCR products. DNA from clones whose colony PCR products were resistant to *Xcm*I digestion was sequenced (Figure 3D). Interestingly, we obtained eight insertional mutations, including one derived from *hCas9* from the vector and seven from the ubiquitin promoter, which were presumably derived from the degraded vector rather than the maize genome (Figure 3E). These results suggest that the efficiency of targeted integration is relatively high when donor genes are provided.

### Validation of the CRISPR/Cas9 toolkit in transgenic maize

**Figure 4:**
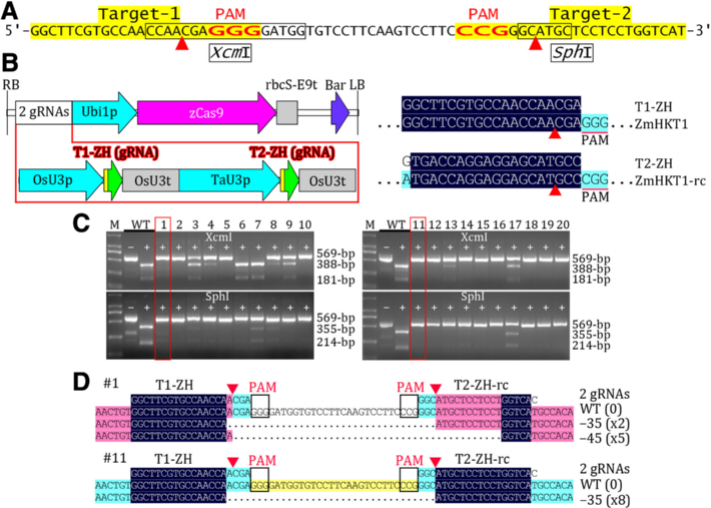
**Validation of the toolkit by targeted mutation of a maize gene. (A)** Sequence of a region of maize *ZmHKT1* with two target sites indicated. **(B)** Physical map of T-DNA carrying two-gRNA expression cassettes. The alignment of target of gRNA with its target gene is shown. Only aligned regions of interest are displayed. -rc, reverse complement. **(C)** Mutation analysis of 20 T0 transgenic lines by *Xcm*I or *Sph*I digestion of PCR fragments. The lines used for sequencing analysis are indicated with boxes. **(D)** Alignment of sequences of mutated alleles identified from cloned PCR fragments from two representative T0 transgenic lines. Highlighting denotes the degree of homology of the aligned fragments, and only aligned regions of interest are displayed. The number of indels of the same type is indicated.

To test the targeted mutation efficiency of the toolkit in monocots, we generated a pCAMBIA-derived CRISPR/Cas9 binary vector with two gRNA expression cassettes targeting the two adjacent sites of the same maize gene, *ZmHKT1* (Figure 4A, B). We analyzed 20 T0 transgenic lines by restriction enzyme digestion of a PCR fragment spanning the two target sites, finding that more than 60% of the transgenic lines had a mutation efficiency of approximately 100% for both target sites (Figure 4C). We cloned and sequenced the PCR fragments from two lines with a mutation efficiency of approximately 100%, finding that sequences between the two target sites were deleted, as shown in Figure 4D. These results indicate that the toolkit can be used for high efficiency targeted mutation in maize and possibly other crops.

### Validation of the CRISPR/Cas9 toolkit in *Arabidopsis* for the generation of mutants with multiple gene mutations

**Figure 5:**
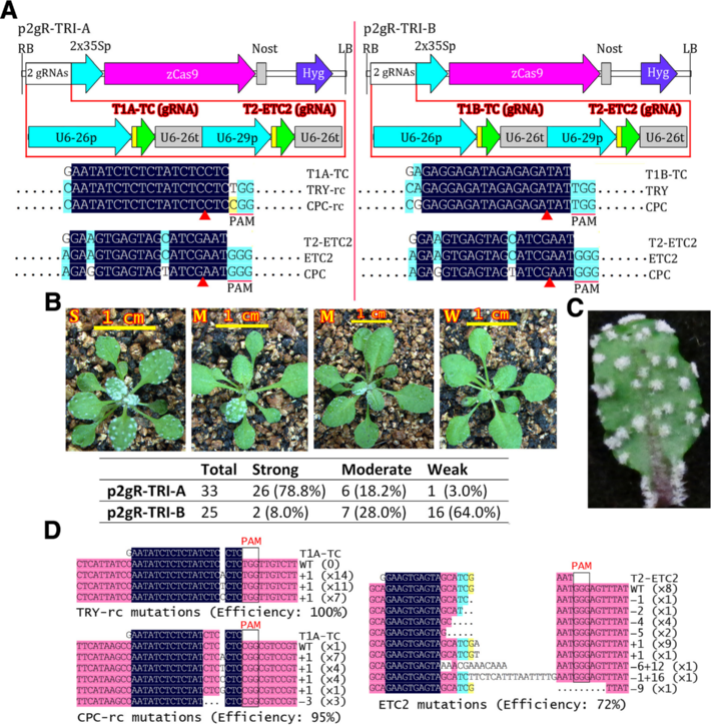
**Validation of the CRISPR/Cas toolkit in** ***Arabidopsis*** **. (A)** Physical maps of the T-DNAs of two pGreen-derived CRISPR/Cas9 binary vectors, each carrying two-gRNAs targeting three *Arabidopsis* genes (*TRY*, *CPC* and *ETC2*). The alignment of gRNA with its target gene is shown. Only aligned regions of interest are displayed. -rc, reverse complement. **(B)** Representative phenotypes of p2gR-TRI-A T1 transgenic lines. S, strong phenotypes similar to that of *try cpc etc2* triple mutant, with highly clustered trichomes on leaf blades and petioles; M, moderate phenotypes with parts of leaf blades or a partial leaf blade displaying the phenotypes of the *try cpc* double mutant or the triple mutant; W, plants with weak or no mutant phenotypes. The total number of T1 transgenic plants, the number of T1 transgenic plants displaying strong, moderate, and weak phenotypes, and the percentage (in parentheses) of the total number are shown. The T0 seeds were screened on hygromycin MS plates for 13 days and grown in soil for 10 days before photographing. **(C)** Magnified image of a detached leaf displaying highly clustered trichomes on petioles, which is similar to the phenotype of the *try cpc etc2* triple mutant. **(D)** Sequencing analysis of target gene mutations of a representative p2gR-TRI-A line. Dots, deleted bases. Highlighting denotes the degree of homology of the aligned fragments. The type of indel and the number of indels of the same type are indicated.

Two vectors, p2gR-TRI-A and p2gR-TRI-B (Figure 5A), each carrying two gRNAs targeting three genes related to trichome development, were used to transform *Arabidopsis*. Both vectors contain the same gRNA (T2-ETC2), which targets *ETC2* and possibly *CPC*, a much less favorable target (Figure 5A). The vectors also contain different gRNAs (T1A-TC or T1B-TC). The 18-bp target sequence in T1A-TC is reversely complementary to that in T1B-TC. Both T1A-TC and T1B-TC target the same two genes: *TRY* and *CPC* (Figure 5A). There is only one mismatch between the 20-nt target of T1A-TC gRNA and *TRY* or *CPC* and between that of T1B-TC and *TRY*, whereas there are two mismatches between that of T1B-TC and *CPC* (Figure 5A). For p2gR-TRI-A, more than 70% of the T1 transgenic plants displayed highly clustered trichomes (Figure 5B,C), as expected for *try cpc* double or *try cpc etc2* triple mutant plants [47]. For p2gR-TRI-B, less than 10% of the plants displayed the expected phenotypes, which suggests that T1B-TC has a much less favorable performance level than T1A-TC. Sequencing of the mutated alleles from a p2gR-TRI-B T1 transgenic line revealed that although the mutation efficiency of the *TRY* allele was more than 90%, that of *CPC* resulting from the same T1B-TC gRNA was only 42% (Additional file 1: Figure S1). By contrast, both *CPC* and *TRY* targeted by the same T1A-TC gRNA had similar mutation frequencies (greater than 90%), regardless of the fact that there were different PAMs between the two target sites (Figure 5A, D). These results suggest that the two mismatches might explain the poor performance of T1B-TC, although the two mismatches are located at the 5’-end of the gRNA. Furthermore, when there were three mismatches between the 20-nt target sequence of T2-ETC2 gRNA and the targeted gene *CPC* (Figure 5A), no mutation was detectable in more than 100 clones from the p2gR-TRI-A transgenic line. By contrast, *ETC2* from the same T2-ETC2 gRNA had a mutation efficiency of 72% (Figure 5D). These results indicate that *in planta*, the CRISPR/Cas9 system has high sequence specificity, and two or more mismatches can greatly reduce the targeting efficiency and off-target effects, especially when a mismatch is near the 3′-end of the 20-nt target of a gRNA.

**Figure 6:**
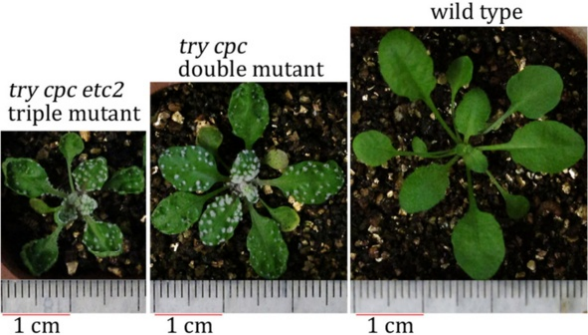
**The** ***try cpc etc2*** **triple mutant can be differentiated from** ***try cpc*** **double mutant.** Representative triple and double mutants and the wild type are shown. The seeds were sown on MS plates, vernalized at 4°C for 3 days, and transferred to an illumination incubator and allowed to grow for 10 days. The seedlings were transplanted to soil and allowed to grow for 17 days before photographing. The triple and double mutants were segregated from A17 T1 lines.

There were many different mutated alleles in a single transgenic plant (Figure 5D), which suggests that the CRISPR/Cas9 functioned after the division of fertilized eggs. To confirm that germline transmission of the mutations into T2 plants is possible, we examined the transmission of mutations of five p2gR-TRI-A T1 transgenic lines with strong phenotypes. Since Cas9 and gRNA are constitutively expressed in T2 transgenic lines, it is sometimes difficult to determine the sources of mutations, which might have arisen from germ cells or somatic cells. On the contrary, mutations from separated T2 nontransgenic plants must result from germline transmission of the mutations from T1 plants. Therefore, we focused on segregated nontransgenic T2 plants to simplify analysis of germline transmission of the mutations. The nontransgenic plants were identified by PCR counterselection with three primer pairs, including two for the hygromycin-resistance gene and one for *Cas9*. We determined the biallelic mutations for both *TRY* and *CPC* based on their clustered trichome phenotypes, finding that the *TRY* and *CPC* mutations were transmitted to T2 plants with high efficiency; 46.2%, 100%, 82.6%, 100%, and 100% of nontransgenic T2 plants derived from five T1 lines, respectively, were biallelic mutants for both *TRY* and *CPC* (Table 1). We first analyzed *ETC2* mutations of nontransgenic T2 double mutant plants by directly sequencing PCR products or by sequencing DNA from different clones harboring the PCR products, and we then analyzed *TRY* and *CPC* mutations of *etc2* mutants verified in the first step of analysis (Table 2). We found that the verified *try cpc etc2* triple mutants could easily be differentiated from *try cpc* double mutants; the former plants were shorter than the latter and had upwardly curled leaves (Figure 6). Biallelic T2 mutants for *ETC2* were segregated from only two T1 lines among the five lines examined (Table 1), demonstrating that the frequency of germline transmission of the *ETC2* mutations into T2 plants was much lower than that of the *TRY* and *CPC* mutations. This result could be explained by the lower mutation frequencies of *ETC2* in T1 plants.

**Table 1:** **Germline transmission of T1 mutations to segregated nontransgenic T2 plants**

**T1 lines**	**Nontransgenic T2 plants**		
	**NT/Total-T2**	**ttcc/NT**	**ttccee/NT**
A1	13/62 (21.0%)	6/13 (46.2%)	0
A17	17/74 (23.0%)	17/17 (100%)	9/17 (52.9%)
A25	23/91 (25.2%)	19/23 (82.6%)	0
A32	16/86 (18.6%)	16/16 (100%)	0
A33	12/51 (23.5%)	12/12 (100%)	3/12 (25.0%)

NT, nontransgenic plants; Total-T2, total number of T2 plants examined. ttcc and ttccee correspond to *try cpc* double and *try cpc etc2* triple mutants, respectively.

**Table 2:** **Mutation analysis of nontransgenic T2 triple mutant plants**

**T1 lines**	**NT T2 triple mutant lines**	***ETC2***	***TRY***	***CPC***
**A17**	A17-1	+A/+A	+C/+C	+T/+T
	A17-2	+A/+A	+C/+T	+T/+T
	A17-3	+A/+A	+G(×2)/+T(×8)	+A/+T
	A17-4	+A/+A	+C(×6)/+T(×3)	+C/+T
	A17-5	+A/+A	+C/+C	+T/+T
	A17-6	+A/+A	+C/+T	+C/+T
	A17-7	+A/+A	+C/+T	+T/+T
	A17-8	+A/+A	+T(×5)/+T(×5)	+T/+T
	A17-9	+A/+A	+T/+T	+C/+T
**A33**	A33-1	+C/+C	-C(×4)/-C(×4)	+G/+G
	A33-2	+A/+C	-C/-C	+G/+G
	A33-3	-TCG/-TCG	+T/+T	+A/+A

Two types of mutations from direct sequencing of PCR products were obtained based on double-peaks on chromatograph. “+” indicates insertion, “–” indicates deletion. Two alleles are separated by “/”. For mutations identified by sequencing of DNA from clones harboring PCR products, the number of clones harboring the same mutation is indicated in parentheses.

**Figure 7:**
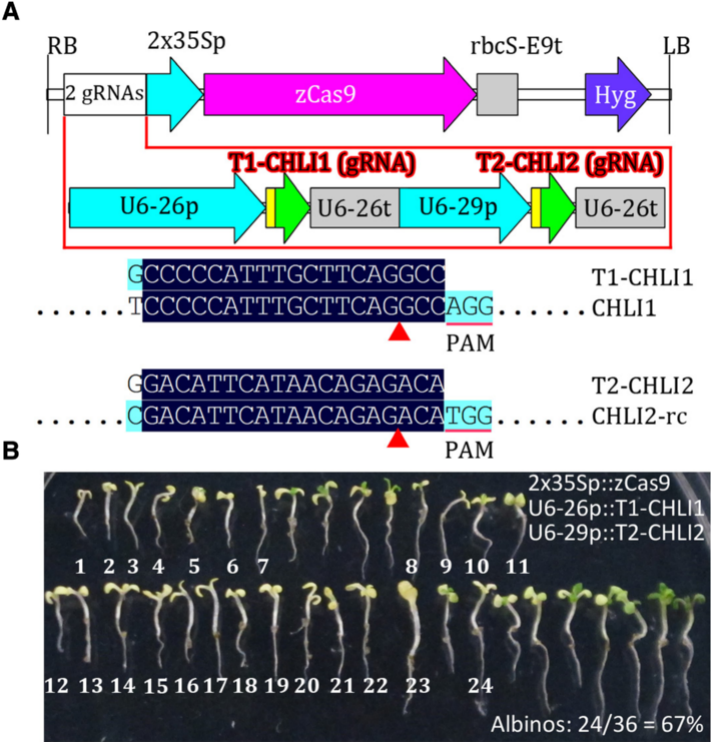
**Validation of pCAMBIA-derived CRISPR/Cas binary vectors in** ***Arabidopsis*** **. (A)** Physical map of T-DNA of the pCAMBIA-derived vector carrying two-gRNAs targeting two *Arabidopsis* genes (*CHLI1* and *CHLI2*). The alignment of gRNA with its target gene is shown. Only aligned regions of interest are displayed. -rc, reverse complement. **(B)** Phenotypes of all transgenic seedlings from one screening. The T0 seeds were screened on hygromycin MS plates for 7 days, and all of the hygromycin-resistant seedlings were transferred to a fresh MS plate before photographing. The albino seedlings were numbered.

To further validate the toolkit in *Arabidopsis*, we constructed a pCAMBIA-based vector, pHSE-2gR-CHLI, carrying two gRNAs targeting *CHLI1* and *CHLI2* (Figure 7), which are the same as the gRNAs employed in a previous study [22]. Simultaneous disruption of *CHLI1* and *CHLI2* led to an albino phenotype, while *chli1* or *chli2* single mutants displayed a pale green phenotype [48]. A higher ratio of T1 transgenic *Arabidopsis* seedlings displayed an albino phenotype (24/36 = 67%) than that reported previously (23/60 = 38%), further demonstrating that the toolkit works well for *Arabidopsis*. Mutation frequencies could be enhanced further through the use of two or more gRNAs to target two or more target sites of the same gene. With the enhanced mutation efficiencies, somatic mutations could be more efficiently transmitted to the next generation. Thus, the toolkit developed in this study could be used to generate *Arabidopsis* mutants with high levels of efficiency and specificity.

## Discussion

Dissecting the functions of gene family members with redundant functions and analyzing epistatic relationships in genetic pathways frequently require plant mutants bearing mutations in multiple genes. The recently developed CRISPR/Cas9 system provides an excellent method for genome editing [4,9,21]. However, to produce multiple gene mutations in plants, resources and methods for the assembly of multiple gRNA expression cassettes are frequently required. In this report, we describe methods used to generate gRNA modules and to assemble multiple gRNA expression cassettes using premade gRNA modules. These resources, comprising binary vectors and gRNA module vectors, are able to meet most of the requirements for use in a variety of plants under normal or complex conditions. These methods also allow researchers to customize their own gRNA modules and to assemble multiple gRNA expression cassettes for multiplex genome editing. Using this kit, we found that CRISPR/Cas9 could be used to knock out multiple plant genes simultaneously, and the efficiencies of multiple-gene mutations, in accordance with the “Bucket effect” theory in economics, depended on the lowest mutation efficiencies of the targeted genes.

Binary vectors are required for the use of CRISPR/Cas9 in plants. To fuse a 20-bp target sequence to the 5′-end of the gRNA scaffold, it is best to use type IIs restriction enzymes. Although a few type IIs restriction enzymes, such as *Ara*I, *Bbs*I/*Bpi*I, *Bsa*I/*Eco*31I, *Bsm*BI/*Esp*3I, *Bsp*MI/*Bfu*AI/*Bve*I, and *Btg*ZI are commercially available, few such enzymes can be used to linearize commonly used binary vectors, such as pCAMBIA series and pPZP series vectors [43,49], due to the presence of one or more sites in the backbones of these vectors. For example, not including the T-DNA region, the pCAMBIA backbone contains one *Bsa*I, two *Bbs*I, two *Bsm*BI, two *Bsp*MI, and four *Btg*ZI sites. Although no *Aar*I site can be found in the pCAMBIA backbone, there is an *Aar*I site in the *Bar* selectable marker gene of the T-DNA region of pCAMBIA3300. Fortunately, despite the presence of a *Bsa*I site in the pVS1 replication region, which is required for plasmid propagation in *Agrobacterium*, there are no *Bsa*I sites in commonly used elements, such as promoters including the double *CaMV 35S* promoter and the *Ubi1* promoter, or in selectable markers including *Kan*, *Hyg* and *Bar*. Moreover, *Bsa*I is the least expensive of the commonly used type IIs restriction enzymes. For example, the price per activity unit of *Bsa*I/*Eco*31I is only approximately 1/50 that of *Aar*I (Thermo Fisher Scientific and New England Biolabs). To utilize *Bsa*I to assemble gRNA expression cassettes into pCAMBIA binary vectors, we disrupted the *Bsa*I site of the pVS1 region. Thus, for the binary vector set we developed, no restriction enzyme but *Bsa*I is required for the assembly of one or more gRNAs.

This toolkit provides the easiest method for generating plant CRISPR/Cas9 binary vectors. When constructing binary vectors carrying one or two gRNAs, only two 23-nt synthetic oligos (annealed to an insert) or a PCR fragment, respectively, are required, along with any of the binary vectors described in this report, to set up Golden Gate reactions. When constructing binary vectors carrying multiple gRNAs, two or more PCR fragments are required. Based on either the Golden Gate cloning method [45] or Gibson Assembly [46], two or more PCR fragments could easily be assembled into multiple gRNA expression cassettes onto any of the *Bsa*I-linearized binary vectors in only one cloning step. Two strategies can be used to assemble more than four gRNA expression cassettes, i.e., generating more gRNA modules with additional validated Pol III promoters, and inserting (for the second time) gRNA expression cassettes harboring the spectinomycin-resistance gene into binary vectors that already contain four gRNAs followed by the assembly of additional gRNAs into the *Bsa*I-linearized vectors. Thus, the binary vector set combined with the gRNA module vector set comprise an efficient, inexpensive, time-saving, user-friendly, multifaceted, extensible toolkit for the generation of CRISPR/Cas9 binary vectors carrying one or more gRNAs for targeted mutations of multiple genes.

## Conclusions

We developed a CRISPR/Cas9-based binary vector set and a gRNA module vector set as a toolkit for multiplex genome editing in plants. We validated the kit using maize protoplasts, maize transgenic lines, and *Arabidopsis* transgenic lines and found that it exhibited high efficiency and specificity. The binary vector set combined with the gRNA module vector set comprise an efficient, inexpensive, time-saving, user-friendly, multifaceted, extensible toolkit for the generation of CRISPR/Cas9 binary vectors carrying one or more gRNAs for targeted mutations of multiple plant genes. This toolkit, which facilitates transient or stable expression of CRISPR/Cas9 in a variety of plant systems, can be applied to a variety of plants and is especially useful for high-efficiency generation of mutants bearing multiple gene mutations.

## Methods

### Vector construction

Detailed descriptions of the vector construction are provided in Additional file 2: Methods S1. All primers used in this report are listed in Additional file 1: Table S1.

### Golden gate method to construct a vector expressing one or two gRNAs

For assembly of one gRNA, equal volumes of 100 μmol/L oligos 1 and 2 were mixed, incubated at 65°C for 5 minutes, and cooled slowly to room temperature, resulting in a double-stranded insert with 4-nt 5′ overhangs at both ends. For assembly of two gRNAs, the two target sites were incorporated into PCR forward and reverse primers, respectively. The PCR fragment was amplified from pCBC-DT1T2 for dicot targets or pCBC-MT1T2 for monocot targets with two long primers or four shorter primers, among which two forward or two reverse primers were partially overlapping. The insert or the purified PCR fragment (T1T2-PCR), together with any of the binary vectors described in this report, were used to set up restriction-ligation reactions, as described elsewhere [44], using *Bsa*I and T4 Ligase (New England Biolabs). The reaction was incubated in a thermocycler for 5 hours at 37°C, 5 min at 50°C and 10 min at 80°C. Detailed information including gRNA module sequences, PCR primers, colony PCR primers, and sequencing primers can be found in Additional file 3: Methods S2.

### Golden gate cloning or Gibson assembly method to generate a vector expressing three or four gRNAs

Two methods were used to assemble more than three gRNAs: Golden Gate Cloning [45] and Gibson Assembly [46]. For Golden Gate Cloning, two (T1-PCR and T2T3-PCR2) or three (T1-PCR, T2-PCR and T3T4-PCR2) PCR fragments were purified and mixed with any of the CRISPR/Cas9 binary vectors to set up Golden Gate reactions as described above. For Gibson Assembly, two (T1T2-PCR and T2T3-PCR) or three (T1T2-PCR, T2T3-PCR and T3T4-PCR) PCR fragments were purified and mixed with Gibson Assembly Master Mix (New England Biolabs) to set up reactions according to the manufacturer’s protocol. The fragment of desired size was gel purified and used as a PCR template for the second round of PCR amplification. The products from the second round of PCR were purified and mixed with any of the binary vectors described in this report to set up the Golden Gate reaction as described above. Detailed information including gRNA module sequences, PCR primers, colony PCR primers, and sequencing primers can be found in Additional file 4: Methods S3 and Additional file 5: Methods S4. The Fusion PCR method was also used to assemble more than three gRNAs; however, the efficiency of the second round of PCR was sometimes greatly reduced due to persistent non-specific amplifications.

### Maize protoplast isolation and transfection

Seeds of B73 maize were immersed in sterile water overnight, sown in soil, and grown under a 16-h light/8-h dark cycle at 22°C in a growth room for 4–6 days. Tissues from the stems and sheaths of 20–30 seedlings were used for protoplast isolation according to a previously described method [50], with one modification, i.e., the protoplast pellets were collected by centrifugation at 100 × *g* for 3 min. PEG-mediated transfections were carried out as described [50]. For each sample, 10–15 μg plasmid DNA was mixed with 200 μL protoplasts (approximately 2 × 10^5^ cells). Freshly prepared PEG solution (200 μL) was added and the mixture was incubated at room temperature for 10–20 min in the dark. Subsequently, 800 μL W5 solution was added and mixed, and the protoplasts were pelleted by centrifugation at 100 × *g* for 3 min. The protoplasts were resuspended in 1.5 mL W5 solution and pelleted by centrifugation at 100 × *g* for 3 min. The protoplast were then resuspended in 800 μL W5 solution and cultured in the dark at 22°C for 14–16 h. Protoplast transfection was performed with three replicates per plasmid.

### Verification of mutations of maize protoplasts

Three transfected protoplast samples from the same vector were pooled and the genomic DNA was extracted. The DNA fragment encompassing the CRISPR target site was amplified from genomic DNA by nested PCR with two pairs of gene-specific primers ZT-IDF0/-IDR0 and ZT-IDF/-IDR (Additional file 1: Table S1). For restriction enzyme digestion analysis of mutations, two restriction enzyme reactions for each PCR product were set up: in one reaction, the corresponding restriction enzyme was added; in the other reaction, the enzyme was replaced by water as a negative control. About 500 ng purified PCR products from each reaction was digested overnight in a 20-μL reaction. Together with the control, digested DNA was separated on a 2.0% ethidium bromide–stained agarose gel. For sequencing analysis of mutations, the purified PCR product was cloned into cloning vector pCBC and the resulting transformants were identified by colony PCR followed by restriction enzyme digestion analysis. Some of the restriction enzymes, such as *Xcm*I and *Sph*I, have activity in *Taq* PCR mixtures. At the end of the PCR, the enzymes were added to the PCR mixtures for overnight digestion, followed by agarose gel electrophoresis analysis. The digestion-resistant fragments were sequenced using a T7 primer.

### Generation of transgenic maize and analysis of mutations

The CRISPR/Cas9 binary vector pBUE-2gRNA-ZH was transformed into *Agrobacterium* strain EHA105, and *Agrobacterium*-mediated method was used to transform immature embryos of B73 maize at China Agricultural University Transgenic Facility Center. The genomic DNA was extracted from 20 transgenic seedlings and the PCR fragment, primers and reactions were the same as those described above. For restriction enzyme digestion analysis, about 500 ng purified PCR products from each reaction was digested overnight with *Xcm*I or *Sph*I in a 20-μL reaction volume. For sequencing analysis, the PCR products from two representative transgenic seedlings were cloned into the cloning vector pCBC and positive clones were sequenced using the T7 primer.

### Generation of transgenic *Arabidopsis* plants and analysis of mutations

The p2gR-TRI-A and p2gR-TRI-B vectors were transformed into *Agrobacterium* strain GV3101/pSoup using the freeze-thaw method, whereas pHSE-2gR-CHLI was transformed into *Agrobacterium* strain GV3101. *Arabidopsis* Col-0 wild-type plants were used for transformation via the floral dip method. The collected seeds were screened on MS plates containing 25 mg/L hygromycin. Genomic DNA was extracted from T1 transgenic plants grown in soil. Fragments surrounding the target sites were amplified by PCR using gene-specific primers TRY-IDF/R, CPC-IDF/R, and ETC2-IDF/R (Additional file 1: Table S1). The purified PCR product was cloned into cloning vector pCBC, and DNA from positive clones for each PCR fragment was sequenced using the T7 primer to identify mutations. To screen segregated nontransgenic T2 plants, genomic DNA was extracted from T2 plants grown in soil. With wild-type genomic DNA serving as a negative control and genomic DNA from T1 transgenic plants serving as a positive control, counterselection PCR was performed with three primer pairs, including Hyg-IDF/R and Hyg-IDF2/R2 for the hygromycin-resistance gene and zCas9-IDF/R for *zCas9* (Additional file 1: Table S1). To analyze mutations of nontransgenic T2 plants, fragments surrounding the target sites of *TRY*, *CPC* or *ETC2* were amplified by PCR using gene-specific primers TRY-IDF0/R0, CPC-IDF0/R0, and ETC2-IDF0/R0 (Additional file 1: Table S1). Purified PCR products were submitted for sequencing with primers (TRY/CPC/ETC2-seqF) located within the PCR fragments (Additional file 1: Table S1). Badly sequenced PCR products were then cloned into cloning vector pCBC and DNA from positive clones was sequenced using the T7 primer.

## Additional files

Additional file 1: Figure S1. Sequencing analysis of target gene mutations of a representative p2gR-TRI-B line. **Table S1.** Primers used in this study. Additional file 2: Methods S1. Vector construction. Additional file 3: Methods S2. Golden Gate cloning method for the assembly of one or two gRNAs. Additional file 4: Methods S3. Golden Gate cloning method for the assembly of three or four gRNAs. Additional file 5: Methods S4. Gibson Assembly method for the assembly of three or four gRNAs.
