# Supplementary material for: A CRISPR/Cas9 toolkit for multiplex genome editing in plants
Source: BMC Plant Biol. 2014 Nov 29;14:327. doi: 10.1186/s12870-014-0327-y (PMC4262988; doi:10.1186/s12870-014-0327-y)
Supplement: Additional file 1: Figure S1. — Sequencing analysis of target gene mutations of a representative p2gR-TRI-B line. Table S1. Primers used in this study. [file 12870_2014_327_MOESM1_ESM.pdf]

Additional file 1

Figure S1

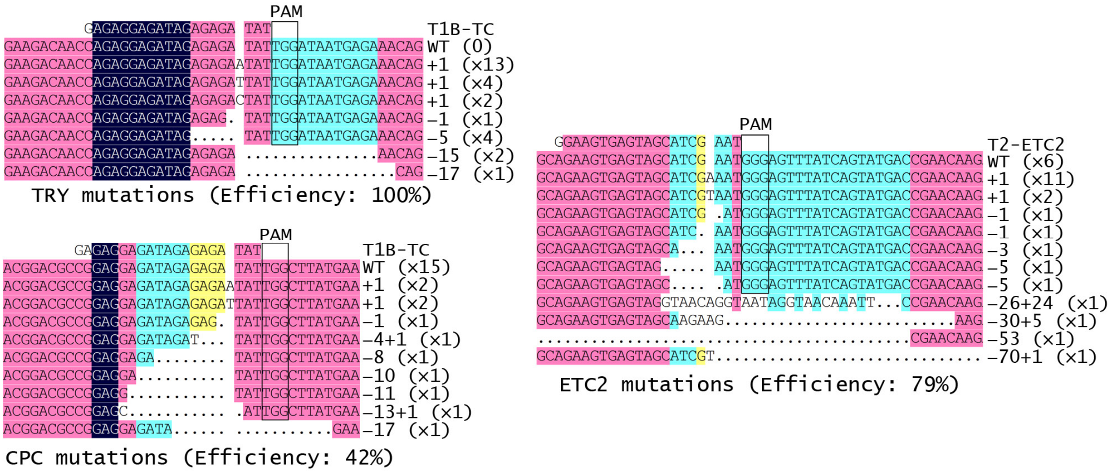

Figure S1 Sequencing analysis of target gene mutations of a representative p2gR-TRI-B Line.

Table S1

Table S1 Primers used in this study

Primer name	Primer sequence (5′→3′)
**Primers for construction of gRNA expression cassettes and premade gRNA modules**	
U6-26-HiF	TCAAGCTTCGACTTGCCTTCCGCACAATACATC
U6-26-HiR	ATCAAGCTTATTGGTTTATCTCATCGGAACTGC
U6-1-HiF	TAAGCTTGAGACGGTCATTGTTTTAGTTCCAC
U6-1-HiR	CAAGCTTAACGGACCAATCACTTTGTCTTAGC
U6-29-HiF	TACAAGCTTAATCCAAACTACTGCAGCCTGAC
U6-29-HiR	TCAAGCTTCATCATATGTCCTCATTCCCTGTC
OsU3-HiF	ATATCAAGCTTAGTAATTCATCCAGGTCTCCAAGTT
OsU3-HiR	AATTCAAGCTTAGCAGCAAGCAGTATCGATCACACC
TaU3-HiF	ATATCAAGCTTCATGAATCCAAACCACACGGAGTTC
TaU3-HiR	ATTATGAAGCTTGTTCGGCTGTCTTGTCACAGAG
Sp-gR-BsF	TGTGAGACCAACCCAGTGGACATAAGC
Sp-gR-R01	AGCCTTATTTTAACTTGCTATTTCTAGCTCTAAAACCGAGACCGGCTTATTATGCACGCT
Sp-gR-R02	AAGCACCGACTCGGTGCCACTTTTTCAAGTTGATAACGGACTAGCCTTATTTTAACTTGCTATTTCTAGC
Sp-gR-DrR	TCTATCTTTAAAAGCACCGACTCGGTGCCACTT
U6-26-hDrF	TTTTGCAAAATTTTCCAGATCGATTTCTTCTTCCTC
U6-26-EcR	TATTAGATATCACTACTTCGACTCTAGCTGTAT

U61-hDrF	TTTTGGCAAAAATTTTCAGATTTTTTCTTCATCTGTAGATTTCTGG
U61-EcR	ATTCAGATATCACTACTTCGTCTCTAACCATA
U6-29-hDrF	TTTTGGATAGAATTTCCCAGCTTTTTTGCGTGTTTCAGC
U6-29-EcR	ATATTAGATATCTCTTAGTCGACTCTACCAAT
OsU3D-NBF	TATATTACCATGGCAGTGACTGGTCTCATT
OsU3D-F0	CCATGGCAGTGACTGGTCTCATTTTTTTTTTCGTTTTGCATTGAGTTTTCTCCGTCGC
OsU3D-NBR	ATTTATCCATGGTCTCAGCCACGGATCATCTGCACAACTCTT
Sp-gR-BBF	ATAATAACGTCTCTTGGCGTGAGACCAACCCAGT
Sp-gR-BsR	AATATTACGTCTCTAAAAGCACCGACTCGGTGCCACTT
TU3D-NBF	ATTACTTACCATGGACATACCTGCAACATT
TU3D-F0	TACCATGGACATACCTGCAACATTTTTTTTTTGTCCTTCTGTTTTTTTAGTCAGTCTC
TU3D-NBR	ATTATATTCCATGGTACCTGCTCGTGCTTCTTGGTGCC
Sp-gR-BBF3	ATAATAACGTCTCTAAGCGTGAGACCAACCCAGT
DT1F0	TGGACGGCCATGGCGGTTCTCGTTTTAGAGCTAGAAATAGC
Ter1Pro2-R	GTCAGGCTGCAGTAGTTTGGATTAATATTGGTTTATCTCATCGGAACTGC
Ter1Pro2-F	GCAGTTCCGATGAGATAAACCAATATTAATCCAAACTACTGCAGCCTGAC
DT2R0	AACTGCCATTGTATAACTTTGCCAATCTCTTAGTCGACTCTAC
DT1F	AATAATGGTCTCTATTGGACGGCCATGGCGGTTCTCGTT
DT2R	ATTATTGGTCTCTAAACTGCCATTGTATAACTTTGC
DT2F0	TGGCAAAGTTATACAATGGCAGTTTTAGAGCTAGAAATAGC
Ter2Pro3-R	GTGGAACTAAAACAATGACCGTCTCATCATATGTCCTCATTCCCTGTC
Ter2Pro3-F	GACAGGGAATGAGGACATATGATGAGACGGTCATTGTTTTAGTTCCAC
DT3R0	AACGATCTAGATTTCCCGGTAACAATCACTACTTCGTCTCTAACCAT
DT2F	AATAATGGTCTCTATTGGCAAAGTTATACAATGGCAGTT
DT3R	ATTATTGGTCTCTAAACGATCTAGATTTCCCGGTAAC
DT3F0	TGTTACCGGGAAATCTAGATCGTTTTAGAGCTAGAAATAGC
Ter3Pro1-R	GATGTATTGTGCGGAAGGCAAGTCGAACGGACCAATCACTTTGTCTTAGC
Ter3Pro1-F	GCTAAGACAAAGTGATTGGTCCGTTCGACTTGCCTTCCGCACAATACATC
DT4R0	AACCATCTTCCATATCTCTTCTCAATCACTACTTCGACTCTAGCTGTAT
DT3F	AATAATGGTCTCTATTGTTACCGGGAAATCTAGATCGTT
DT4R	ATTATTGGTCTCTAAACCATCTTCCATATCTCTTCT
MT-F	GTTTTAGAGCTAGAAATAGCAAGTT
MTer1Pro2-R0	GAACTCCGTGTGGTTTGGATTCATGAGCAGCAAGCAGTATCGATCACACC
MTer1Pro2-F0	GGTGTGATCGATACTGCTTGCTGCTCATGAATCCAAACCACACGGAGTTC
MT2-R	GGCATGCTCCTCCTGGTCACGCTTCTTGGTGCC
MTer2Pro3-R0	GATGTATTGTGCGGAAGGCAAGTCGTGTTCGGCTGTCTTGTCACAGAG
MTer2Pro3-F0	CTCTGTGACAAGACAGCCGAACACGACTTGCCTTCCGCACAATACATC
MT3-R	TATCACTACTTCGACTCTAGCTGTAT
MTer3Pro4-R0	AACTTGGTGACCTGGATGAATTACTTATTGGTTTATCTCATCGGAACTGC
MTer3Pro4-F0	GCAGTTCCGATGAGATAAACCAATAAGTAATTCATCCAGGTCACCAAGTT
MT4-R	CGCCACGGATCATCTGCACAACTCTT
**Primers for construction of pGreen-like binary vectors**	
pSa-ori-XbF	ATTATTTCTAGATCCCCATCCAACAGCCCGC

pSa-ori-XbR	ATTATTCTAGACCATGAATCCAGAAGCCCGAGAGGT
Marker-EcF	ATTTAGAATTCGAGGCGGTTTGCGTATTGGCTAGAG
Marker-SpR	ATTTAACTAGTGACAACTTAATAACACATTGCGGAC
**Primers for construction of the plant CRISPR/Cas9 vector set**	
oXX-F	TGTATGGCCGCGGCCGCACCGGTAGGCCTCCATACAA
oXX-R	TGTATGGAGGCCTACCGGTGCGGCCGCGGCCATACAA
**Primers for construction of one-gRNA-expressing vectors for gene targeting**	
Oligo-01F/ZT1	ATTGGCTTCGTGCCAACCAACGA
Oligo-R/ZT1	AAACTCGTTGGTTGGCACGAAGC
Oligo-11F/ZT1	GGCGGCTTCGTGCCAACCAACGA
Oligo-21F/ZT1	AGCGGCTTCGTGCCAACCAACGA
**Primers for construction of two-gRNA-expressing vectors for gene targeting**	
MT1-BsF/ZH	AATAATGGTCTCTGGCGGCTTCGTGCCAACCAACGA
MT1-F0/ZH	GGCTTCGTGCCAACCAACGAGTTTTAGAGCTAGAAATAGC
MT2-R0/ZH	GGCATGCTCCTCCTGGTCACGCTTCTTGGTGCC
MT2-BsR/ZH	ATTATTGGTCTCTAAACGGCATGCTCCTCCTGGTCA
DT1A-BsF/TC	AATAATGGTCTCTATTGAATATCTCTCTATCTCCTCGTT
DT1A-F0/TC	TGAATATCTCTCTATCTCCTCGTTTTAGAGCTAGAAATAGC
DT2-R0/ETC2	AACATTCGATGCTACTCACTTCCAATCTCTTAGTCGACTCTAC
DT2-BsR/ETC2	ATTATTGGTCTCTAAACATTCGATGCTACTCACTTC
DT1B-BsF/TC	AATAATGGTCTCTATTGAGAGGAGATAGAGAGATATGTT
DT1B-F0/TC	TGAGAGGAGATAGAGAGATATGTTTTAGAGCTAGAAATAGC
DT1-BsF/CHLI1	AATAATGGTCTCTATTG-CCCCCATTTGCTTCAGGCCGTT
DT1-F0/CHLI1	CCCCCATTTGCTTCAGGCC-GTTTTAGAGCTAGAAATAGC
DT2-R0/CHLI2	TGTCTCTGTTATGAATGTCCAATCTCTTAGTCGACTCTAC
DT2-BsR/CHLI2	ATTATTGGTCTCTAAACTGTCTCTGTTATGAATGTC
**Primers for analysis on target gene mutations**	
ZT-IDF0	AAGGACGGCCATGGCGATTTCGCAT
ZT-IDR0	CCGATGATCTTCTGATAGATGGATC
ZT-IDF	TATCGTGCTGGGCTACATCTTGGTC
ZT-IDR	CGGAGTGCCTGGAGTTGACGGACAT
TRY-IDF	ATGTACAGACTTGTCGGTGATAGGT
TRY-IDR	GGACGGTGAGGCTTGGTATGTTTGT
CPC-IDF	GGTCTAACTTACCGAGCTGTCAATG
CPC-IDR	CAAAATAGTAATTCAAGGACAGGTACAT
CPC-off-IDF	GTTTCGTGTCTTCAGATTAGTTCGATGT
CPC-off-IDR	TCATTGACAGCTCGGTAAGTTAGAC
ETC2-IDF	CAGTAGTTATGGATAATACCAACCGTCT
ETC2-IDR	ATCAGCTTTGATTTGTTACTCTCGCCAT
TRY-IDF0	ATGTACAGACTTGTCGGTGATAGGT
TRY-IDR0	GTCTCATGGATTCGTTGTATAGCGT
TRY-SeqF	CATGTATATAAATGCTTGGCTGGCTCAA
CPC-IDF0	GGTCTAACTTACCGAGCTGTCAATG

CPC-IDR0	GAGCTACCTCGTTGACCCATATCGT
CPC-SeqF	TAATTTATGTCAGAACTCACTTTGGCTA
ETC2-IDF0	CCTCACATCTTTTCTATATCTTGCATTC
ETC2-IDR0	TTCCTGCTATTAAATCCCACCTGAC
ETC2-SeqF	GTTCACTCGATCCCGATATGACTCT
**Primers for counterselection of nontransgenic T2 plants**	
Hyg-IDF1	CCCGATTCCGGAAGTGCTTGACATT
Hyg-IDR1	CGCTCCAGTCAATGACCGCTGTTAT
Hyg-IDF2	CAAAGATCGTTATGTTTATCGGCACT
Hyg-IDR2	AAGAAGATGTTGGCGACCTCGTATT
zCAS9-IDF	CGGCCTCGATATTGGGACTAACTCT
zCAS9-IDR	CTTATCTGTGGAGTCCACGAGCTTC
