# Supplementary material for: A CRISPR/Cas9 toolkit for multiplex genome editing in plants
Source: BMC Plant Biol. 2014 Nov 29;14:327. doi: 10.1186/s12870-014-0327-y (PMC4262988; doi:10.1186/s12870-014-0327-y)
Supplement: Additional file 2: Methods S1. — Vector construction. [file 12870_2014_327_MOESM2_ESM.pdf]

Additional file 2

Methods S1

Vector construction

Table of contents

Construction of gRNA expression cassettes and gRNA modules 2

Construction of pGreen-like binary vectors 3

Construction of pGreen-derived CRISPR/Cas binary vectors 4

Construction of pCAMBIA-derived CRISPR/Cas binary vectors........................... 5

Construction of one-gRNA-expressing vectors for gene targeting....................... 5

Construction of vectors each expressing two gRNAs 5

References 6

Construction of gRNA expression cassettes and gRNA modules

All of the PCR fragments for cloning purposes were amplified with proof-reading DNA polymerase Phusion (NEB) or KOD plus (Toyobo), and verified by sequencing. All primers used in this report are listed in Additional file 1: Table S1.

AtU6-26, *AtU6-1*, and *AtU6-29* snRNA genes amplified from Arabidopsis genomic DNA using primer pairs U6-26-HiF/-HiR, U6-1-HiF/-HiR, and U6-29-HiF/-HiR, respectively, were cloned into pCBC, a blunt-end PCR cloning vector, resulting in pCBC-U626/U61/U629. *OsU3* and *TaU3* genes were cloned into pCBC from rice and wheat genomic DNA using primer pairs OsU3-HiF/-HiR and TaU3-HiF/-HiR, respectively, resulting in pCBC-OsU3/TaU3. SpR-gRNA fusion gene was amplified from pPZP200 [1] with four primers Sp-gR-BsF/-R01/-R02/-DrR. The pCBC-U626 backbone including the *AtU6-26* promoter and terminator region but excluding *U6* snRNA sequence was amplified with primer pairs U6-26-hDrF/-EcR. The SpR-gRNA PCR fragment was digested with *Dra*I and the pCBC-U626 PCR fragment was digested with *Eco*RV, and then the two fragments were ligated, resulting in pCBC-U626p-gRS. In a similar manner, pCBC-U61p-gRS and pCBC-U629p-gRS were generated with primer pairs U6-1-hDrF/-EcR and U6-29-hDrF/-EcR, respectively. The pCBC-OsU3 backbone including the *OsU3* promoter and terminator region but excluding the *U3* snRNA sequence was amplified with three primers OsU3D-NBF/-F0/-NBR, the PCR fragment was digested with *Nco*I and then ligated to allow for re-cyclization, resulting in pCBC-OsU3Del. The SpR-gRNA fragment amplified from pCBC-U626p-gRS with primer pair Sp-gR-BBF/-BsR was digested with *Bsm*BI and then ligated with *Bsa*I-digested pCBC-OsU3Del, resulting in pCBC-OsU3p-gRS. The pCBC-TaU3 backbone including the *TaU3* promoter and terminator region but excluding the *U3* snRNA sequence was amplified with three primers TaU3D-NBF/-F0/-NBR, then the PCR fragment was digested with *Nco*I, and finally ligated to allow for re-cyclization, resulting in pCBC-TaU3Del. The SpR-gRNA fragment amplified from pCBC-U626p-gRS with primer pair Sp-gR-BBF3/-BsR was digested with *Bsm*BI and then ligated with *Bfu*AI-digested pCBC-TaU3Del, resulting in pCBC-TaU3p-gRS.

Two PCR fragments amplified from pCBC-U626p-gRS with primer pair DT1F0/Ter1Pro2-R and from pCBC-U629p with primer pair Ter1Pro2-F/DT2R0 were assembled into one PCR fragment by fusion PCR with primer pair DT1F/-T2R, the fragment was then cloned into pCBC, resulting in pCBC-DT1T2. In a similar manner, pCBC-DT2T3 and pCBC-DT3T4 were generated using three primer pairs: DT2F0/Ter2Pro3-R, Ter2Pro3-F/DT3R0, and DT2F/-T3R, and three primer pairs: DT3F0/Ter3Pro1-R, Ter3Pro1-F/DT4R0, and DT3F/-T4R, respectively. The pCBC-DT1T2/-DT2T3/-DT3T4 carry Target_1-gRNA-U6_26t -U6_29p-Target_2, Target_2-gRNA-U6_29t-U6_1p-Target_3, and Target_3-gRNA-U6_1t-U6_26p-Target_4 constructs, respectively.

To express in monocot plants, pCBC-MT1T2/-MT2T3/-MT3T4 carrying Target_1-gRNA-OsU3t -TaU3p-Target_2, Target_2-gRNA-TaU3t-U6-26p-Target_3, Target_3-gRNA-U6-26t-OsU3p-Target_4 constructs, respectively, were also generated by Fusion PCR with primers MT-F/MTer1Pro2-R0/-F0/MT2-R, MT-F/MTer2Pro3-R0/-F0/MT3-R, and MT-F/MTer3Pro4-R0/-F0/MT4-R, respectively.

Construction of pGreen-like binary vectors

The pSa-ori PCR fragment amplified from pCH32 (Hamilton, 1997) with primer pair pSa-ori-XbF/-XbR was digested with *Xba*I and ligated with *Nhe*I digested pENTR1A (Invitrogen), resulting in pGLP. A synthetic LB-RB fragment digested with *Afl*II and *Pst*I was inserted into the *Afl*II-*Pst*I sites of pGLP, resulting in pGL. *Hyg* and *Bar* expression cassettes amplified from pLC-Hyg and pLC-Bar [2], respectively, with primer pair Marker-EcF/SpR were digested with *Eco*RI and *Spe*I and inserted into the *Eco*RI-*Spe*I sites of pGL, resulting in pHGL and pBGL. The *Hin*dIII-*Eco*RI fragment for p2x35S-tNos from pXSN [3] was inserted into the *Hin*dIII-*Eco*RI sites of the pHGL, resulting in pHSN-ccdB. The *Hin*dIII-*Eco*RI fragment for *ubi1p* from pXUN [3] was inserted into the *Hin*dIII-*Eco*RI sites of the pHGL, resulting in pHUN-ccdB. The *Hin*dIII-*Sac*I fragment for pUbi-tNos from pXUN [3] was inserted into the *Hin*dIII-*Sac*I sites of the pBGL, resulting in pBUN-ccdB.

Construction of pGreen-derived CRISPR/Cas binary vectors

To generate the CRISPR/Cas vector set, an insert annealed with oXX-F/-R oligos was ligated with *Xcm*I digested pHSN-ccdB, pHUN-ccdB and pBUN-ccdB, respectively, resulting in pHSN000, pHUN000 and pBUN000. The pCBC-U626p-gRS was digested with *Hin*dIII and ligated with *Hin*dIII-digested pHSN000 and pBUN000, respectively, resulting in pHSN001 and pBUN001. The pJDS246 [4] was digested with *Not*I and *Pme*I and ligated with *Not*I and *Stu*I digested pBUN001, resulting in pBUN201. An intermediate vector carrying multiple cloning sites *Not*I-*Age*I-*Eco*RI-*Stu*I was constructed, and then the *Age*I-*Eco*RI fragment of pX330 [5] was inserted between the *Age*I and *Eco*RI sites of the intermediate vector, resulting in pCBC-Cas9-3. The pCBC-Cas9-3 was digested with *Not*I and *Stu*I, and ligated with *Not*I and *Stu*I digested pBUN001, resulting in pBUN301. In a similar manner to the constructionof the pHSN/BUN301, pHSN/BUN401 were generated by using *Not*I and *Stu*I digested pUC57-zCas9. In pUC57-zCas9, the DNA sequence of Cas9 was codon-optimized with maize favored codons and synthesized and cloned into pUC57 by GenScript (Nanjing). The pBUN401 were digested with *Hin*dIII and ligated to allow for re-cyclization, resulting in pBUN400. The pCBC-OsU3p-gRS and pCBC-TaU3p-gRS were digested with *Hin*dIII and ligated with *Hin*dIII digested pBUN400, resulting in pBUN411/421. The *Eco*RI-*Spe*I fragment of *Hyg* expression cassette in the pHSN401 was replaced with the *Eco*RI-*Spe*I fragment of *Bar* from the pBUN401, resulting in pBSN401. *Kan* expression cassette amplified from pLC-Kan [2], with primer pair Marker-EcF/SpR was digested with *Eco*RI and *Spe*I and inserted between the *Eco*RI and *Spe*I sites of pHSN401 to replace the *Hyg* expression cassette with *Kan*, resulting in pKSN401. The *Not*I-*Stu*I fragment of pUC57-zCas9 was inserted between the *Not*I and *Stu*I sites of pHUN000, resulting in pHUN400. The *Hin*dIII frament of pCBC-OsU3-gRS was inserted into the *Hin*dIII site of the pHUN400, resulting in pHUN411.

Construction of pCAMBIA-derived CRISPR/Cas binary vectors

A 635-bp pea rbcS E9 terminator (E9t) was amplified by PCR, digested with *Xho*I and *Spe*I, inserted between the *Xho*I and *Spe*I sites of the pHSN-ccdB. The *Hin*dIII-*Eco*RI fragment of 2x35Sp-MCS-E9t was then inserted between the *Hin*dIII and *Eco*RI sites of pMDC99 (derived from pCAMBIA1300), pMDC100 (derived from pCAMBIA2300), and pMDC123 (derived from pCAMBIA3300) [6], resulting in pHSE/pKSE/pBSE. The 403-bp *Bsa*I-*Nhe*I fragment of the pVS1 region of pHSE/pKSE/pBSE was replaced with the 403-bp PCR fragment, thus one point mutation was introduced, leading to the *Bsa*I site disrupted and resulting in pHSEm/pKSEm/pBSEm. The *Xba*I-*Sac*I fragment of *zCas9* from pUC57-zCas9 was inserted between the *Xba*I and *Sac*I sites of the 2x35Sp-MCS-E9t of the pH/K/BSEm, resulting in pHSE400/pKSE400/pBSE400. The *Hin*dIII fragment of the gRNA expression cassette from pCBC-U626p-gRS was inserted into the *Hin*dIII site of the pH/K/BSE400, resulting in pHSE401/pKSE401/pBSE401. The *Hin*dIII-*Xba*I fragment of 2x35S promoter of pHSE400/pBSE400 was replaced with a *Hin*dIII-*Spe*I fragment of *Ubi1* promoter, resulting in pHUE400/pBUE400. The *Hin*dIII fragment of the gRNA expression cassette from the pCBC-OsU3p-gRS was inserted into the *Hin*dIII site of the pHUE400/pBUE400, resulting in pHUE411/pBUE411.

Construction of one-gRNA-expressing vectors for gene targeting

Oligo-01F/ZT1 and Oligo-R/ZT1 were annealed and inserted between two *Bsa*I sites of pBUN201/301/401, resulting in pBUN201/301/401-ZT1. Oligo-11F/ZT1 and Oligo-R/ZT1 were annealed and inserted between two *Bsa*I sites of pBUN411, resulting in pBUN411-ZT1. Oligo-21F/ZT1 and Oligo-R/ZT1 were annealed and inserted between two *Bsa*I sites of pBUN421, resulting in pBUN421-ZT1.

Construction of two-gRNA-expressing vectors for gene targeting

The PCR fragment amplified from pCBC-MT1T2 with four primers MT1-BsF,

-F0/ZH and MT2-R0, -BsR/ZH was inserted between the *Bsa*I sites of pBUE411 by Golden Gate cloning, resulting in pBUE-2gR-ZH. The PCR fragment amplified from pCBC-DT1T2 with four primers DT1A-BsF, -F0/TC and DT2-R0, -BsR/ETC2 was inserted between the *Bsa*I sites of pHSN401 by Golden Gate cloning, resulting in p2gR-TRI-A. In a similar manner, p2gR-TRI-B was generated using four primers DT1B-BsF, -F0/TC and DT2-R0, -BsR/ETC2. The PCR fragment amplified from pCBC-DT1T2 with four primers DT1-BsF, -F0/CHLI1 and DT2-R0, -BsR/CHLI2 was inserted between the *Bsa*I sites of pHSE401 by Golden Gate cloning, resulting in pHSE-2gR-CHLI.

References

1. Hajdukiewicz P, Svab Z, Maliga P: **The small, versatile pPZP family of Agrobacterium binary vectors for plant transformation**. *Plant Mol Biol* 1994, 25:989-994.
2. Chen QJ, Xie M, Ma XX, Dong L, Chen J, Wang XC: **MISSA is a highly efficient in vivo DNA assembly method for plant multiple-gene transformation**. *Plant Physiol* 2010, 153:41-51.
3. Chen S, Songkumarn P, Liu J, Wang GL: **A versatile zero background T-vector system for gene cloning and functional genomics**. *Plant Physiol* 2009, 150:1111-1121.
4. Hwang WY, Fu Y, Reyon D, Maeder ML, Tsai SQ, Sander JD, Peterson RT, Yeh JR, Joung JK: **Efficient genome editing in zebrafish using a CRISPR-Cas system.** *Nat Biotechnol* 2013, **31:**227-229.
5. Cong L, Ran FA, Cox D, Lin S, Barretto R, Habib N, Hsu PD, Wu X, Jiang W, Marraffini LA, Zhang F: **Multiplex genome engineering using CRISPR/Cas systems.** *Science* 2013, **339:**819-823.
6. Curtis MD, Grossniklaus U: **A gateway cloning vector set for high-throughput functional analysis of genes in planta**. *Plant Physiol* 2003, 133:462-469.
