# Supplementary material for: A CRISPR/Cas9 toolkit for multiplex genome editing in plants
Source: BMC Plant Biol. 2014 Nov 29;14:327. doi: 10.1186/s12870-014-0327-y (PMC4262988; doi:10.1186/s12870-014-0327-y)
Supplement: Additional file 3: Methods S2. — Golden Gate cloning method for the assembly of one or two gRNAs. [file 12870_2014_327_MOESM3_ESM.pdf]

Additional file 3

Methods S2
Golden Gate cloning method for the assembly of one or two gRNAs

Table of contents

Simplified protocol 2
Table S2-1 Nomenclature of inserts/oligos and PCR products/primers 3
Sequence of maize codon-optimized Cas9 (zCas9) 4
Sequence of SpR-gRNA-Scaffold 5
Sequence of U6-26p-driven gRNA expression cassette without target 6
Sequence of DT1T2-PCR with Targets 1 and 2 for dicots 7
Sequence of one gRNA expression cassette for dicots 8
Sequence of two gRNA expression cassettes for dicots 9
Sequence of OsU3p-driven gRNA expression cassette without target 10
Sequence of MT1T2-PCR with Targets 1 and 2 for monocots 11
Sequence of one gRNA expression cassette for monocots 12
Sequence of two gRNA expression cassettes for monocots 13

Simplified protocol

1. Manually search for 23-bp target sites (5'-N_{20}NGG-3') within exons of genomic DNA sequences of genes of interest, and then evaluate target specificities on the website of potential off-target finder (http://www.rgenome.net/cas-offinder/). Users can also search for target sites on the website of genome-wide prediction of plant CRISPR/Cas9 target sites (http://www.genome.arizona.edu/crispr/CRISPRsearch.html).
2. Design primers:
    a) Find names of inserts/oligos (for one gRNA) or PCR fragments/primers (for two gRNAs) according to plant species (monocots or dicots) and gRNA numbers in Table S2-1.
    b) Find the sequences of the oligos/primers according to the names.
    c) Replace 19-nt N in the forward primers with your 19-nt target sequences in front of PAM (NGG), and 19-nt N in the reverse primers with reverse complement sequences of your 19-nt target sequences in front of PAM (NGG).
3. Carry out PCR reactions according to information provided under the sequences of the PCR fragments. As an example, the reaction mixture and reaction conditions are as follows for construction of pHSE401-2gR-CHLI:

Component	Volume	Cycling conditions
10× KOD plus Buffer	5 µl	1. One cycle: 94 °C, 2 min.<br>2. 30 cycles: 94 °C, 15 sec; 60 °C, 30 sec; 68 °C, 1 min.<br>3. One cycle: 68 °C, 5 min
MgSO4 (25mM)	3 µl	
dNTPs (2mM, Toyobo)	4 µl	
KOD plus (Toyobo)	1 µl	
pCBC-DT1T2 (diluted to 200 times)	1 µl	
DT1-BsF (20 µM)	1 µl	
DT1-F0 (1 µM)	1 µl	
DT2-R0 (1 µM)	1 µl	
DT2-BsR (20 µM)	1 µl	
ddH_2O	32 µl	
Total volume	50 µl	

4. Set up Golden Gate reactions as follows:

Component	Volume	Reaction conditions
Purified PCR framents (~100 ng/µl)	2 µl	5 hours at 37°C<br>5 min at 50°C<br>10 min at 80°C<br><br>NOTE: It is essential to use a High Concentration (HC) Ligase (2 million units/ml, NEB)
pHSE401 or others (~100 ng/µl)	2 µl	
10× T4 DNA Ligase Buffer (NEB)	1.5 µl	
10× BSA	1.5 µl	
*Bsa*I (NEB)	1 µl	
T4 DNA Ligase (HC, NEB)	1 µl	
ddH_2O	6 µl	
Total volume	15 µl	

5. Transform *E.coli* competent cells with 5 µl of reaction mixture, and select positive clones on kanamycin LB agar plates.
6. Identify correct clones by colony PCR and verify them by sequencing.

Table S2-1 Nomenclature of inserts/oligos and PCR products/primers

For dicots		
Insert or PCR product	Oligos or Primers	Number of gRNAs
01-insert	Oligo-01-F<br>Oligo-R	1
DT1T2-PCR	DT1-BsF/DT1-F0<br>DT2-R0/DT2-BsR	2
**For monocots**		
Insert or PCR product	Oligos or Primers	Number of gRNAs
11-insert	Oligo-11-F<br>Oligo-R	1
MT1T2-PCR	MT1-BsF/MT1-F0<br>MT2-R0/MT2-BsR	2

Notes:

1. Dilute the F0/R0 primers to 20 times of the F/R primers.

Sequence of maize codon-optimized Cas9 (zCas9)

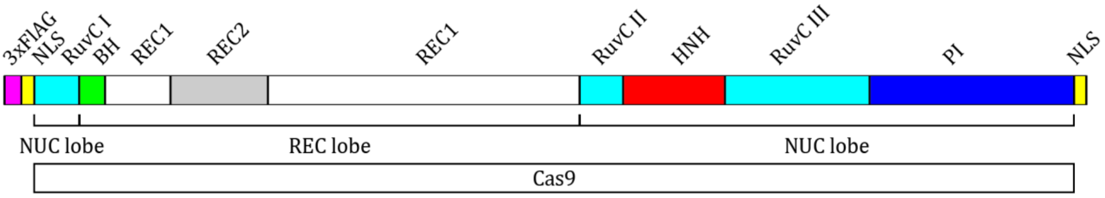

ATGGATTACAAGGACCACGACGGGGATTACAAGGACCACGACATTGATTACAAGGATGATGATGACAAGATGGCTCCGAAGAAGAAGAGGAAGGTTGGC
ATCCACGGGGTGCCAGCTGCTGACAAGAAGTACTCGATCGGCCTCGATATTGGGACTAACTCTGTTGGCTGGGCCGTGATCACCGACGAGTACAAGGTGC
CCTCAAAGAAGTTCAAGGTCCTGGGCAACACCGATCGGCATTCCATCAAGAAGAATCTCATTGGCGCTCTCCTGTTCGACAGCGGCGAGACGGCTGAGG
CTACGCGGCTCAAGCGCACCGCCCGCAGGCGGTACACGCGCAGGAAGAATCGCATCTGCTACCTGCAGGAGATTTTCTCCAACGAGATGGCGAAGGTTG
ACGATTCTTTCTTCCACAGGCTGGAGGAGTCATTCCTCGTGGAGGAGGATAAGAAGCACGAGCGGCATCCAATCTTCGGCAACATTGTCGACGAGGTTGC
CTACCACGAGAAGTACCCTACGATCTACCATCTGCGGAAGAAGCTCGTGGACTCCACAGATAAGGCGGACCTCCGCCTGATCTACCTCGCTCTGGCCCACA
TGATTAAGTTCAGGGGCCATTTCCTGATCGAGGGGGATCTCAACCCGGACAATAGCGATGTTGACAAGCTGTTCATCCAGCTCGTGCAGACGTACAACCA
GCTCTTCGAGGAGAACCCCATTAATGCGTCAGGCGTCGACGCGAAGGCTATCCTGTCCGCTAGGCTCTCGAAGTCTCGGCGCCTCGAGAACCTGATCGCC
CAGCTGCCGGGCGAGAAGAAGAACGGCCTGTTCGGGAATCTCATTGCGCTCAGCCTGGGGCTCACGCCCAACTTCAAGTCGAATTTCGATCTCGCTGAG
GACGCCAAGCTGCAGCTCTCCAAGGACACATACGACGATGACCTGGATAACCTCCTGGCCCAGATCGGCGATCAGTACGCGGACCTGTTCCTCGCTGCCA
AGAATCTGTCGGACGCCATCCTCCTGTCTGATATTCTCAGGGTGAACACCGAGATTACGAAGGCTCCGCTCTCAGCCTCCATGATCAAGCGCTACGACGAG
CACCATCAGGATCTGACCCTCCTGAAGGCGCTGGTCAGGCAGCAGCTCCCCGAGAAGTACAAGGAGATCTTCTTCGATCAGTCGAAGAACGGCTACGCTG
GGTACATTGACGGCGGGGCCTCTCAGGAGGAGTTCTACAAGTTCATCAAGCCGATTCTGGAGAAGATGGACGGCACGGAGGAGCTGCTGGTGAAGCTC
AATCGCGAGGACCTCCTGAGGAAGCAGCGGACATTCGATAACGGCAGCATCCCACACCAGATTCATCTCGGGGAGCTGCACGCTATCCTGAGGAGGCAG
GAGGACTTCTACCCTTTCCTCAAGGATAACCGCGAGAAGATCGAGAAGATTCTGACTTTCAGGATCCCGTACTACGTCGGCCCACTCGCTAGGGGCAACTC
CCGCTTCGCTTGGATGACCCGCAAGTCAGAGGAGACGATCACGCCGTGGAACTTCGAGGAGGTGGTCGACAAGGGCGCTAGCGCTCAGTCGTTCATCGA
GAGGATGACGAATTTCGACAAGAACCTGCCAAATGAGAAGGTGCTCCCTAAGCACTCGCTCCTGTACGAGTACTTCACAGTCTACAACGAGCTGACTAAG
GTGAAGTATGTGACCGAGGGCATGAGGAAGCCGGCTTTCCTGTCTGGGGAGCAGAAGAAGGCCATCGTGGACCTCCTGTTCAAGACCAACCGGAAGGT
CACGGTTAAGCAGCTCAAGGAGGACTACTTCAAGAAGATTGAGTGCTTCGATTCGGTCGAGATCTCTGGCGTTGAGGACCGCTTCAACGCCTCCCTGGGG
ACCTACCACGATCTCCTGAAGATCATTAAGGATAAGGACTTCCTGGACAACGAGGAGAATGAGGATATCCTCGAGGACATTGTGCTGACACTCACTCTGTT
CGAGGACCGGGAGATGATCGAGGAGCGCCTGAAGACTTACGCCCATCTCTTCGATGACAAGGTCATGAAGCAGCTCAAGAGGAGGAGGTACACCGGCT
GGGGGAGGCTGAGCAGGAAGCTCATCAACGGCATTCGGGACAAGCAGTCCGGGAAGACGATCCTCGACTTCCTGAAGAGCGATGGCTTCGCGAACCGC
AATTTCATGCAGCTGATTCACGATGACAGCCTCACATTCAAGGAGGATATCCAGAAGGCTCAGGTGAGCGGCCAGGGGGACTCGCTGCACGAGCATATCG
CGAACCTCGCTGGCTCGCCAGCTATCAAGAAGGGGATTCTGCAGACCGTGAAGGTTGTGGACGAGCTGGTGAAGGTCATGGGCAGGCACAAGCCTGAG
AACATCGTCATTGAGATGGCCCGGGAGAATCAGACCACGCAGAAGGGCCAGAAGAACTCACGCGAGAGGATGAAGAGGATCGAGGAGGGCATTAAGG
AGCTGGGGTCCCAGATCCTCAAGGAGCACCCGGTGGAGAACACGCAGCTGCAGAATGAGAAGCTCTACCTGTACTACCTCCAGAATGGCCGCGATATGTA
TGTGGACCAGGAGCTGGATATTAACAGGCTCAGCGATTACGACGTCGATCATATCGTTCCACAGTCATTCCTGAAGGATGACTCCATTGACAACAAGGTCC
TCACCAGGTCGGACAAGAACCGGGGCAAGTCTGATAATGTTCCTTCAGAGGAGGTCGTTAAGAAGATGAAGAACTACTGGCGCCAGCTCCTGAATGCCA
AGCTGATCACGCAGCGGAAGTTCGATAACCTCACAAAGGCTGAGAGGGGCGGGCTCTCTGAGCTGGACAAGGCGGGCTTCATCAAGAGGCAGCTGGTC
GAGACACGGCAGATCACTAAGCACGTTGCGCAGATTCTCGACTCACGGATGAACACTAAGTACGATGAGAATGACAAGCTGATCCGCGAGGTGAAGGTC
ATCACCCTGAAGTCAAAGCTCGTCTCCGACTTCAGGAAGGATTTCCAGTTCTACAAGGTTCGGGAGATCAACAATTACCACCATGCCCATGACGCGTACCT
GAACGCGGTGGTCGGCACAGCTCTGATCAAGAAGTACCCAAAGCTCGAGAGCGAGTTCGTGTACGGGGACTACAAGGTTTACGATGTGAGGAAGATGAT
CGCCAAGTCGGAGCAGGAGATTGGCAAGGCTACCGCCAAGTACTTCTTCTACTCTAACATTATGAATTTCTTCAAGACAGAGATCACTCTGGCCAATGGCG
AGATCCGGAAGCGCCCCCTCATCGAGACGAACGGCGAGACGGGGGAGATCGTGTGGGACAAGGGCAGGGATTTCGCGACCGTCAGGAAGGTTCTCTCC
ATGCCACAAGTGAATATCGTCAAGAAGACAGAGGTCCAGACTGGCGGGTTCTCTAAGGAGTCAATTCTGCCTAAGCGGAACAGCGACAAGCTCATCGCCC
GCAAGAAGGACTGGGATCCGAAGAAGTACGGCGGGTTCGACAGCCCCACTGTGGCCTACTCGGTCCTGGTTGTGGCGAAGGTTGAGAAGGGCAAGTCC
AAGAAGCTCAAGAGCGTGAAGGAGCTGCTGGGGATCACGATTATGGAGCGCTCCAGCTTCGAGAAGAACCCGATCGATTTCCTGGAGGCGAAGGGCTA
CAAGGAGGTGAAGAAGGACCTGATCATTAAGCTCCCCAAGTACTCACTCTTCGAGCTGGAGAACGGCAGGAAGCGGATGCTGGCTTCCGCTGGCGAGCT
GCAGAAGGGGAACGAGCTGGCTCTGCCGTCCAAGTATGTGAACTTCCTCTACCTGGCCTCCCACTACGAGAAGCTCAAGGGCAGCCCCGAGGACAACGA
GCAGAAGCAGCTGTTCGTCGAGCAGCACAAGCATTACCTCGACGAGATCATTGAGCAGATTTCCGAGTTCTCCAAGCGCGTGATCCTGGCCGACGCGAAT
CTGGATAAGGTCCTCTCCGCGTACAACAAGCACCGCGACAAGCCAATCAGGGAGCAGGCTGAGAATATCATTCATCTCTTCACCCTGACGAACCTCGGCG
CCCCTGCTGCTTTCAAGTACTTCGACACAACTATCGATCGCAAGAGGTACACAAGCACTAAGGAGGTCCTGGACGCGACCCTCATCCACCAGTCGATTACC
GGCCTCTACGAGACGCGCATCGACCTGTCTCAGCTCGGGGGCGACAAGCGGCCAGCGGCGACGAAGAAGGCGGGGCAGGCGAAGAAGAAGTGA

Sequence of SpR-gRNA-Scaffold

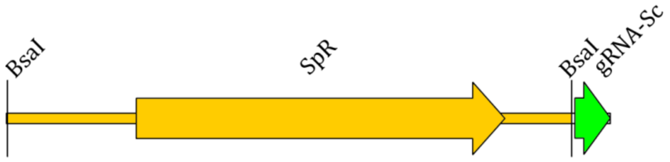

(BsaI)-(SpR)-(BsaI)-(gRNA-Sc)

GAGACCAACCCAGTGGACATAAGCCTGTTCGGTTCGTAAGCTGTAATGCAAGTAGCGTATGCGCT
CACGCAACTGGTCCAGAACCTTGACCGAACGCAGCGGTGGTAACGGCGCAGTGGCGGTTTTCATGGCTTG
TTATGACTGTTTTTTTGGGGTACAGTCTATGCCTCGGGCATCCAAGCAGCAAGCGCGTTACGCCGTGGGTCG
ATGTTTGATGTTATGGAGCAGCAACGATGTTACGCAGCAGGGCAGTCGCCCTAAAACAAAGTTAAACATCAT
GGGGGAAGCGGTGATCGCCGAAGTATCGACTCAACTATCAGAGGTAGTTGGCGTCATCGAGCGCCATCTCG
AACCGACGTTGCTGGCCGTACATTTGTACGGCTCCGCAGTGGATGGCGGCCTGAAGCCACACAGTGATATT
GATTTGCTGGTTACGGTGACCGTAAGGCTTGATGAAACAACGCGGCGAGCTTTGATCAACGACCTTTTGGA
AACTTCGGCTTCCCCTGGAGAGAGCGAGATTCTCCGCGCTGTAGAAGTCACCATTGTTGTGCACGACGACAT
CATTCCGTGGCGTTATCCAGCTAAGCGCGAACTGCAATTTGGAGAATGGCAGCGCAATGACATTCTTGCAGG
TATCTTCGAGCCAGCCACGATCGACATTGATCTGGCTATCTTGCTGACAAAAGCAAGAGAACATAGCGTTGC
CTTGGTAGGTCCAGCGGCGGAGGAACTCTTTGATCCGGTTCCTGAACAGGATCTATTTGAGGCGCTAAATGA
AACCTTAACGCTATGGAACTCGCCGCCCGACTGGGCTGGCGATGAGCGAAATGTAGTGCTTACGTTGTCCCG
CATTTGGTACAGCGCAGTAACCGGCAAAATCGCGCCGAAGGATGTCGCTGCCGACTGGGCAATGGAGCGC
CTGCCGGCCCAGTATCAGCCCGTCATACTTGAAGCTAGACAGGCTTATCTTGGACAAGAAGAAGATCGCTTG
GCCTCGCGCGCAGATCAGTTGGAAGAATTTGTCCACTACGTGAAAGGCGAGATCACCAAGGTAGTCGGCAA
ATAATGTCTAGCTAGAAATTCGTTCAAGCCGACGCCGCTTCGCGGCGCGGCTTAACTCAAGCGTTAGATGCA
CTAAGCACATAATTGCTCACAGCCAAACTATCAGGTCAAGTCTGCTTTTATTATTTTTAAGCGTGCATAATAAG
CC**GGTCTC**GGTTTTAGAGCTAGAAATAGCAAGTTAAAATAAGGCTAGTCCGTTATCAACTTGAAAA
AGTGGCACCGAGTCGGTGC

Notes:

1. SpR, spectinomycin-resistance gene; gRNA-Sc, gRNA scaffold.
2. Enlarged and boxed letters indicate *Bsa*I sites.

Sequence of U6-26p-driven gRNA expression cassette without target

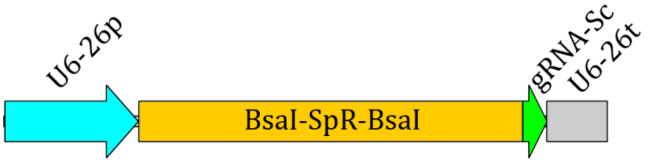

(U6-26p)-(BsaI-SpR-BsaI)-(gRNA-Sc)-(U6-26t)

CGACTTGCCTTCCGCACAATACATCATTTCTTCTTAGCTTTTTTTCTTCTTCTTCGTTCATACAGTTTTTTTTTGTTTATCAGCTTAC
ATTTTCTTGAACCGTAGCTTTCGTTTTCTTCTTTTTAACTTTCCATTCGGAGTTTTTGTATCTTGTTTCATAGTTTGTCCCAGGATTA
GAATGATTAGGCATCGAACCTTCAAGAATTTGATTGAATAAAACATCTTCATTCTTAAGATATGAAGATAATCTTCAAAAGGCCCC
TGGGAATCTGAAAGAAGAGAAGCAGGCCCATTTATATGGGAAAGAACAATAGTATTTCTTATATAGGCCCATTTAAGTTGAAAAC
AATCTTCAAAAGTCCCACATCGCTTAGATAAGAAAACGAAGCTGAGTTTATATACAGCTAGAGTCGAAGTAGTGATTGGGA
GACCAACCCAGTGGACATAAGCCTGTTCGGTTCGTAAGCTGTAATGCAAGTAGCGTATGCGCTCACGCAACTGGTCCAG
AACCTTGACCGAACGCAGCGGTGGTAACGGCGCAGTGGCGGTTTTCATGGCTTGTTATGACTGTTTTTTTGGGGTACAGTCTAT
GCCTCGGGCATCCAAGCAGCAAGCGCGTTACGCCGTGGGTCGATGTTTGATGTTATGGAGCAGCAACGATGTTACGCAGCAGG
GCAGTCGCCCTAAAACAAAGTTAAACATCATGGGGGAAGCGGTGATCGCCGAAGTATCGACTCAACTATCAGAGGTAGTTGGC
GTCATCGAGCGCCATCTCGAACCGACGTTGCTGGCCGTACATTTGTACGGCTCCGCAGTGGATGGCGGCCTGAAGCCACACAG
TGATATTGATTTGCTGGTTACGGTGACCGTAAGGCTTGATGAAACAACGCGGCGAGCTTTGATCAACGACCTTTTGGAAACTTC
GGCTTCCCCTGGAGAGAGCGAGATTCTCCGCGCTGTAGAAGTCACCATTGTTGTGCACGACGACATCATTCCGTGGCGTTATCC
AGCTAAGCGCGAACTGCAATTTGGAGAATGGCAGCGCAATGACATTCTTGCAGGTATCTTCGAGCCAGCCACGATCGACATTGA
TCTGGCTATCTTGCTGACAAAAGCAAGAGAACATAGCGTTGCCTTGGTAGGTCCAGCGGCGGAGGAACTCTTTGATCCGGTTC
CTGAACAGGATCTATTTGAGGCGCTAAATGAAACCTTAACGCTATGGAACTCGCCGCCCGACTGGGCTGGCGATGAGCGAAAT
GTAGTGCTTACGTTGTCCCGCATTTGGTACAGCGCAGTAACCGGCAAAATCGCGCCGAAGGATGTCGCTGCCGACTGGGCAAT
GGAGCGCCTGCCGGCCCAGTATCAGCCCGTCATACTTGAAGCTAGACAGGCTTATCTTGGACAAGAAGAAGATCGCTTGGCCT
CGCGCGCAGATCAGTTGGAAGAATTTGTCCACTACGTGAAAGGCGAGATCACCAAGGTAGTCGGCAAATAATGTCTAGCTAGA
AATTCGTTCAAGCCGACGCCGCTTCGCGGCGCGGCTTAACTCAAGCGTTAGATGCACTAAGCACATAATTGCTCACAGCCAAAC
TATCAGGTCAAGTCTGCTTTTATTATTTTTAAGCGTGCATAATAAGCCGGTCTCGGTTTTAGAGCTAGAAATAGCAAGTT
AAAATAAGGCTAGTCCGTTATCAACTTGAAAAAGTGGCACCGAGTCGGTGCTTTTTTTTGCAAAATTTTCCAGATCGATTTCTTC
TTCCTCTGTTCTTCGGCGTTCAATTTCTGGGGTTTTCTCTTCGTTTTCTGTAACTGAAACCTAAAATTTGACCTAAAAAAAATCTCA
AATAATATGATTCAGTGGTTTTGTACTTTTCAGTTAGTTGAGTTTTGCAGTTCCGATGAGATAAACCAATA

Notes:

1. SpR, spectinomycin-resistance gene; gRNA-Sc, gRNA scaffold.
2. Enlarged and boxed letters indicate *Bsa*I sites.

Sequence of DT1T2-PCR with Targets 1 and 2 for dicots

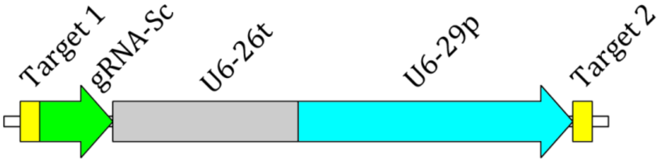

(Target-1)-(gRNA-Sc)-(U6-26t)-(U6-29p)-(Target-2)

ATATATGGTCTCGATTGNNNNNNNNNNNNNNNNNNNGTTTTAGAGCTAGAAATAGCAAGTTAAAATAAG
GCTAGTCCGTTATCAACTTGAAAAAGTGGCACCGAGTCGGTGCTTTTTTTTGCAAAATTTTCCAGATCGATTT
CTTCTTCCTCTGTTCTTCGGCGTTCAATTTCTGGGGTTTTCTCTTCGTTTTCTGTAACTGAAACCTAAAATTTG
ACCTAAAAAAAATCTCAAATAATATGATTCAGTGGTTTTGTACTTTTCAGTTAGTTGAGTTTTGCAGTTCCGAT
GAGATAAACCAATATTAATCCAAACTACTGCAGCCTGACAGACAAATGAGGATGCAAACAATTTTAAAGTTT
ATCTAACGCTAGCTGTTTTGTTTCTTCTCTCTGGTGCACCAACGACGGCGTTTTCTCAATCATAAAGAGGCT
TGTTTTACTTAAGGCCAATAATGTTGATGGATCGAAAGAAGAGGGCTTTTAATAAACGAGCCCGTTTAAG
CTGTAAACGATGTCAAAAACATCCCACATCGTTCAGTTGAAAATAGAAGCTCTGTTTATATATTGGTAGAG
TCGACTAAGAGATTGNNNNNNNNNNNNNNNNNNNGTTTAGAGACCAATAAT

Primers:

DT1-BsF: ATATATGGTCTCGATTGNNNNNNNNNNNNNNNNNNNGTT

DT1-F0: TGNNNNNNNNNNNNNNNNNNNGTTTTAGAGCTAGAAATAGC

DT2-R0: AACNNNNNNNNNNNNNNNNNNNCAATCTCTTAGTCGACTCTAC

DT2-BsR: ATTATTGGTCTCGAAACNNNNNNNNNNNNNNNNNNNCAA

Template: pCBC-DT1T2

Length: 626-bp

Notes:

1. The 19-nt N in primers represent any 19-nt target sequence (forward primers) or reverse complement sequence of any 19-nt target sequence (reverse primers) in front of PAM (NGG).
2. For the assembly of two gRNA expression cassettes, use DT1-BsF/DT1-F0/DT2-R0/DT2-BsR four-primer mixture with DT1-F0/DT2-R0 diluted to 20 times of DT1-BsF or DT2-BsR, resulting in DT1T2-PCR.

Sequence of one gRNA expression cassette for dicots

23-bp insert + pHSN401 et al.

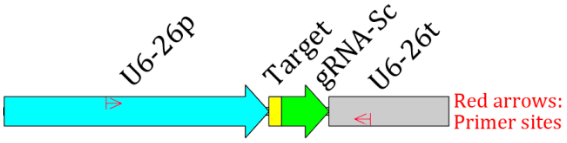

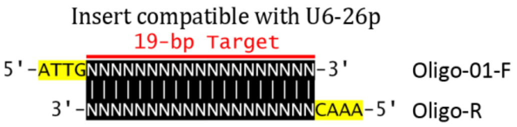

(U6-26p)-(Target-1)-(gRNA-Sc)-(U6-26t)

CGACTTGCCTTCCGCACAATACATCATTTCTTCTTAGCTTTTTTTCTTCTTCTTCGTTCATACAGTTTTTTTTTGT
TTATCAGCTTACATTTTCTTGAACCGTAGCTTTCGTTTTCTTCTTTTTAACTTTCCATTCGGAGTTTTTGTATCTT
GTTTCATAGTT**TGTCCCAGGATTAGAATGATTAGGC**ATCGAACCTTCAAGAATTTGATTGAATAAAACATCTT
CATTCTTAAGATATGAAGATAATCTTCAAAAGGCCCCTGGGAATCTGAAAGAAGAGAAGCAGGCCCATTTATA
TGGGAAAGAACAATAGTATTTCTTATATAGGCCCATTTAAGTTGAAAACAATCTTCAAAAGTCCCACATCGCTT
AGATAAGAAAACGAAGCTGAGTTTATATACAGCTAGAGTCGAAGTAGTGATTGNNNNNNNNNNNNNNNN
NNNGTTTTAGAGCTAGAAATAGCAAGTTAAAATAAGGCTAGTCCGTTATCAACTTGAAAAAGTGGCACCGA
GTCGGTGCTTTTTTTTGCAAAATTTTCCAGATCGATTTCTTCTTCCTCT**GTTCTTCGGCGTTCAATTTCTGGGG**
TTTTCTCTTCGTTTTCTGTAACTGAAACCTAAAATTTGACCTAAAAAAAATCTCAAATAATATGATTCAGTGGTT
TTGTACTTTTCAGTTAGTTGAGTTTTGCAGTTCCGATGAGATAAACCAATA

Notes:

1. Underlined letters come from binary vectors, while the others come from PCR fragments.
2. Red letters indicate primer sites.
3. Primer sequences are as follows:

Colony PCR primers (5'→3'):

U6-26p-F: TGTCCCAGGATTAGAATGATTAGGC

U6-26t-R: CCCCAGAAATTGAACGCCGAAGAAC

(U6-26p-F + U6-26t-R = 423 bp)

Sequencing primers (5'→3'):

U6-26p-F: TGTCCCAGGATTAGAATGATTAGGC

Sequence of two gRNA expression cassettes for dicots

DT1DT2-PCR + pHSN401 et al.

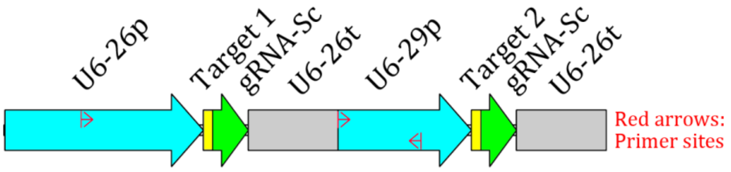

(U6-26p)-(Target-1)-(gRNA-Sc)-(U6-26t)-(U6-29p)-(Target-2)-(gRNA-Sc)-(U6-26t)

CGACTTGCCTTCCGCACAATACATCATTTCTTCTTAGCTTTTTTTCTTCTTCTTCGTTCATACAGTTTTTTTTTGT
TTATCAGCTTACATTTTCTTGAACCGTAGCTTTCGTTTTCTTCTTTTTAACTTTCCATTCGGAGTTTTTGTATCTT
GTTTCATAGTT**TGTCCCAGGATTAGAATGATTAGGC**ATCGAACCTTCAAGAATTTGATTGAATAAAACATCTT
CATTCTTAAGATATGAAGATAATCTTCAAAAGGCCCCTGGGAATCTGAAAGAAGAGAAGCAGGCCCATTTATA
TGGGAAAGAACAATAGTATTTCTTATATAGGCCCATTTAAGTTGAAAACAATCTTCAAAAGTCCCACATCGCTT
AGATAAGAAAACGAAGCTGAGTTTATATACAGCTAGAGTCGAAGTAGTGATTGNNNNNNNNNNNNNNNN
NNNGTTTTAGAGCTAGAAATAGCAAGTTAAAATAAGGCTAGTCCGTTATCAACTTGAAAAAGTGGCACCGA
GTCGGTGCTTTTTTTTGCAAAATTTTCCAGATCGATTTCTTCTTCCTCTGTTCTTCGGCGTTCAATTTCTGGGG
TTTTCTCTTCGTTTTCTGTAACTGAAACCTAAAATTTGACCTAAAAAAAATCTCAAATAATATGATTCAGTGGTT
TTGTACTTTTCAGTTAGTTGAGTTTTGCAGTTCCGATGAGATAAACCAATA**TTAATCCAAACTACTGCAGCCT**
GACAGACAAATGAGGATGCAAACAATTTTAAAGTTTATCTAACGCTAGCTGTTTTGTTTCTTCTCTCTGGTG
CACCAACGACGGCGTTTTCTCAATCATAAAGAGGCTTGTTTTACTTAAGGCCAATAAT**GTTGATGGATCGA**
AAGAAGAGGGCTTTTAATAAACGAGCCCGTTTAAGCTGTAAACGATGTCAAAAACATCCCACATCGTTCA
GTTGAAAATAGAAGCTCTGTTTATATATTGGTAGAGTCGACTAAGAGATTGNNNNNNNNNNNNNNNNN
NNGTTTTAGAGCTAGAAATAGCAAGTTAAAATAAGGCTAGTCCGTTATCAACTTGAAAAAGTGGCACCGAGT
CGGTGCTTTTTTTTGCAAAATTTTCCAGATCGATTTCTTCTTCCTCTGTTCTTCGGCGTTCAATTTCTGGGGTT
TTCTCTTCGTTTTCTGTAACTGAAACCTAAAATTTGACCTAAAAAAAATCTCAAATAATATGATTCAGTGGTTTT
GTACTTTTCAGTTAGTTGAGTTTTGCAGTTCCGATGAGATAAACCAATA

Notes:

1. Underlined letters come from binary vectors, while the others come from PCR fragments.
2. Red letters indicate primer sites.
3. Primer sequences are as follows:

Colony PCR primers (5'→3'):

U6-26p-F: TGTCCCAGGATTAGAATGATTAGGC

U6-29p-R: AGCCCTCTTCTTTCGATCCATCAAC

(U6-26p-F + U6-29p-R = 726 bp)

Sequencing primers (5'→3'):

U6-26p-F: TGTCCCAGGATTAGAATGATTAGGC

U6-29p-F: TTAATCCAAACTACTGCAGCCTGAC

Sequence of OsU3p-driven gRNA expression cassette without target

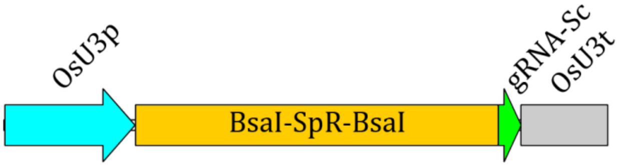

(OsU3p)-(BsaI-SpR-BsaI)-(gRNA-Sc)-(OsU3t)

AGTAATTCATCCAGGTCTCCAAGTTCTAGGATTTTCAGAACTGCAACTTATTTTATCAAGGAATCTTTAAACATACGAACAGATCA
CTTAAAGTTCTTCTGAAGCAACTTAAAGTTATCAGGCATGCATGGATCTTGGAGGAATCAGATGTGCAGTCAGGGACCATAGCA
CAAGACAGGCGTCTTCTACTGGTGCTACCAGCAAATGCTGGAAGCCGGGAACACTGGGTACGTTGGAAACCACGTGATGTGA
AGAAGTAAGATAAACTGTAGGAGAAAAGCATTTCGTAGTGGGCCATGAAGCCTTTCAGGACATGTATTGCAGTATGGGCCGGC
CCATTACGCAATTGGACGACAACAAAGACTAGTATTAGTACCACCTCGGCTATCCACATAGATCAAAGCTGATTTAAAAGAGTTG
TGCAGATGATCCGTGGCGT**GAGACC**AACCCAGTGGACATAAGCCTGTTCGGTTCGTAAGCTGTAATGCAAGTAGCGT
ATGCGCTCACGCAACTGGTCCAGAACCTTGACCGAACGCAGCGGTGGTAACGGCGCAGTGGCGGTTTTCATGGCTTGTTATGA
CTGTTTTTTTGGGGTACAGTCTATGCCTCGGGCATCCAAGCAGCAAGCGCGTTACGCCGTGGGTCGATGTTTGATGTTATGGAG
CAGCAACGATGTTACGCAGCAGGGCAGTCGCCCTAAAACAAAGTTAAACATCATGGGGGAAGCGGTGATCGCCGAAGTATCG
ACTCAACTATCAGAGGTAGTTGGCGTCATCGAGCGCCATCTCGAACCGACGTTGCTGGCCGTACATTTGTACGGCTCCGCAGTG
GATGGCGGCCTGAAGCCACACAGTGATATTGATTTGCTGGTTACGGTGACCGTAAGGCTTGATGAAACAACGCGGCGAGCTTT
GATCAACGACCTTTTGGAAACTTCGGCTTCCCCTGGAGAGAGCGAGATTCTCCGCGCTGTAGAAGTCACCATTGTTGTGCACG
ACGACATCATTCCGTGGCGTTATCCAGCTAAGCGCGAACTGCAATTTGGAGAATGGCAGCGCAATGACATTCTTGCAGGTATCT
TCGAGCCAGCCACGATCGACATTGATCTGGCTATCTTGCTGACAAAAGCAAGAGAACATAGCGTTGCCTTGGTAGGTCCAGCG
GCGGAGGAACTCTTTGATCCGGTTCCTGAACAGGATCTATTTGAGGCGCTAAATGAAACCTTAACGCTATGGAACTCGCCGCCC
GACTGGGCTGGCGATGAGCGAAATGTAGTGCTTACGTTGTCCCGCATTTGGTACAGCGCAGTAACCGGCAAAATCGCGCCGAA
GGATGTCGCTGCCGACTGGGCAATGGAGCGCCTGCCGGCCCAGTATCAGCCCGTCATACTTGAAGCTAGACAGGCTTATCTTG
GACAAGAAGAAGATCGCTTGGCCTCGCGCGCAGATCAGTTGGAAGAATTTGTCCACTACGTGAAAGGCGAGATCACCAAGGT
AGTCGGCAAATAATGTCTAGCTAGAAATTCGTTCAAGCCGACGCCGCTTCGCGGCGCGGCTTAACTCAAGCGTTAGATGCACTA
AGCACATAATTGCTCACAGCCAAACTATCAGGTCAAGTCTGCTTTTATTATTTTTAAGCGTGCATAATAAGCC**GGTCTC**G
GTTTTAGAGCTAGAAATAGCAAGTTAAAATAAGGCTAGTCCGTTATCAACTTGAAAAAGTGGCACCGAGTCGGTGCTTTTTTTTT
TCGTTTTGCATTGAGTTTTCTCCGTCGCATGTTTGCAGTTTTATTTTCCGTTTTGCATTGAAATTTCTCCGTCTCATGTTTGCAGCG
TGTTCAAAAAGTACGCAGCTGTATTTCACTTATTTACGGCGCCACATTTTCATGCCGTTTGTGCCAACTATCCCGAGCTAGTGAAT
ACAGCTTGGCTTCACACAACACTGGTGACCCGCTGACCTGCTCGTACCTCGTACCGTCGTACGGCACAGCATTTGGAATTAAAG
GGTGTGATCGATACTGCTTGCTGCT

Notes:

1. The OsU3p contains a *Bsa*I site affected by Dcm methylation and therefore uncuttable when vectors are proliferated in commonly used *E. coli* strains (dcm+).
2. Enlarged and boxed letters indicate *Bsa*I sites.

Sequence of MT1T2-PCR with Targets 1 and 2 for monocots

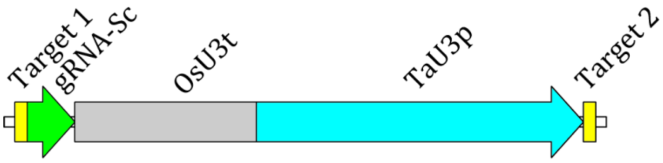

(Target-1)-(gRNA-Sc)-(OsU3t)-(TaU3p)-(Target-2)

ATATATGGTCTCTGGCGNNNNNNNNNNNNNNNNNNNGTTTTAGAGCTAGAAATAGCAAGTTAAAATAAG
GCTAGTCCGTTATCAACTTGAAAAAGTGGCACCGAGTCGGTGCTTTTTTTTTTCGTTTTGCATTGAGTTTTCT
CCGTCGCATGTTTGCAGTTTTATTTTCCGTTTTGCATTGAAATTTCTCCGTCTCATGTTTGCAGCGTGTTCAAA
AAGTACGCAGCTGTATTTCACTTATTTACGGCGCCACATTTTCATGCCGTTTGTGCCAACTATCCCGAGCTAGT
GAATACAGCTTGGCTTCACACAACACTGGTGACCCGCTGACCTGCTCGTACCTCGTACCGTCGTACGGCACA
GCATTTGGAATTAAAGGGTGTGATCGATACTGCTTGCTGCTCATGAATCCAAACCACACGGAGTTCAAATTCC
CACAGATTAAGGCTCGTCCGTCGCACAAGGTAATGTGTGAATATTATATCTGTCGTGCAAAATTGCCTGGCCT
GCACAATTGCTGTTATAGTTGGCGGCAGGGAGAGTTTTAACATTGACTAGCGTGCTGATAATTTGTGAGAAA
TAATAATTGACAAGTAGATACTGACATTTGAGAAGAGCTTCTGAACTGTTATTAGTAACAAAAATGGAAAGCT
GATGCACGGAAAAAGGAAAGAAAAAGCCATACTTTTTTTTAGGTAGGAAAAGAAAAAGCCATACGAGACT
GATGTCTCTCAGATGGGCCGGGATCTGTCTATCTAGCAGGCAGCAGCCCACCAACCTCACGGGCCAGCAATT
ACGAGTCCTTCTAAAAGCTCCCGCCGAGGGGCGCTGGCGCTGCTGTGCAGCAGCACGTCTAACATTAGTCC
CACCTCGCCAGTTTACAGGGAGCAGAACCAGCTTATAAGCGGAGGCGCGGCACCAAGAAGCGNNNNNNN
NNNNNNNNNNNNGTTTAGAGACCAATAAT

Primers:

MT1-BsF: ATATATGGTCTCTGGCGNNNNNNNNNNNNNNNNNNNGTT

MT1-F0: TGNNNNNNNNNNNNNNNNNNNGTTTTAGAGCTAGAAATAGC

MT2-R0: AACNNNNNNNNNNNNNNNNNNNCGCTTCTTGGTGCC

MT2-BsR: ATTATTGGTCTCTAAACNNNNNNNNNNNNNNNNNNNC

Template: pCBC-MT1T2

Length: 964-bp

Notes:

1. The 19-nt N in primers represent any 19-nt target sequence (forward primers) or reverse complement sequence of any 19-nt target sequence (reverse primers) in front of PAM (NGG).
2. For the assembly of two gRNA expression cassettes, use MT1-BsF/MT1-F0/MT2-R0/MT2-BsR four-primer mixture with MT1-F0/MT2-R0 diluted to 20 times of MT1-BsF or MT2-BsR, resulting in MT1T2-PCR.

Sequence of one gRNA expression cassette for monocots

23-bp insert + pBUN411 et al.

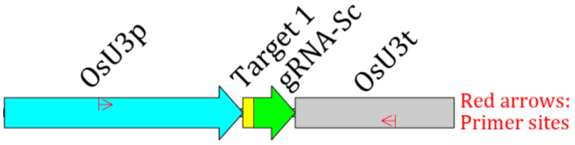

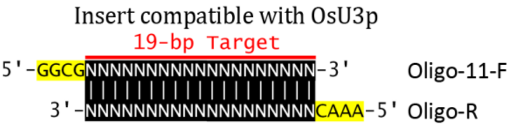

(OsU3p)-(Target-1)-(gRNA-Sc)-(OsU3t)

AGTAATTCATCCAGGTCTCCAAGTTCTAGGATTTTCAGAACTGCAACTTATTTTATCAAGGAATCTTTAAACATACGAACAGATCA
CTTAAAGTTCTTCTGAAGCAACTTAAAGTTATCAGGCATGCATGGATCTTGGAGGAATCAGATGTGCAGTCAGGGACCATAGCA
CAA**GACAGGCGTCTTCTACTGGTGCTAC**CAGCAAATGCTGGAAGCCGGGAACACTGGGTACGTTGGAAACCACGTGATGTGA
AGAAGTAAGATAAACTGTAGGAGAAAAGCATTTCGTAGTGGGCCATGAAGCCTTTCAGGACATGTATTGCAGTATGGGCCGGC
CCATTACGCAATTGGACGACAACAAAGACTAGTATTAGTACCACCTCGGCTATCCACATAGATCAAAGCTGATTTAAAAGAGTTG
TGCAGATGATCCGTGGCGNNNNNNNNNNNNNNNNNNNGTTTTAGAGCTAGAAATAGCAAGTTAAAATAAGGCTAGTCCGTT
ATCAACTTGAAAAAGTGGCACCGAGTCGGTGCTTTTTTTTTTCGTTTTGCATTGAGTTTTCTCCGTCGCATGTTTGCAGTTTTATT
TTCCGTTTTGCATTGAAATTTCTCCGTCTCATGTTTGCAGCGTGTTCAAAAAGTACGCAGCTGTATTTCACTTATTTACGGCGCCA
CATTTTCATGCCGTTTGT**GCCAACTATCCCGAGCTAGTGAATA**CAGCTTGGCTTCACACAACACTGGTGACCCGCTGACCTGCT
CGTACCTCGTACCGTCGTACGGCACAGCATTTGGAATTAAAGGGTGTGATCGATACTGCTTGCTGCT

Notes:

1. Underlined letters come from binary vectors, while the others come from PCR fragments.
2. The OsU3p from vectors contains a *Bsa*I site affected by Dcm methylation and therefore uncuttable when vectors are proliferated in commonly-used *E. coli* strains (dcm+).
3. Red letters indicate primer sites.
4. Primer sequences are as follows:

Colony PCR primers (5'→3'):

OsU3p-F3: GACAGGCGTCTTCTACTGGTGCTAC

OsU3t-R: TATTCACTAGCTCGGGATAGTTGGC

(OsU3p-F3 + OsU3t-R = 543 bp)

Sequencing primers (5'→3'):

OsU3p-F3: GACAGGCGTCTTCTACTGGTGCTAC

Sequence of two gRNA expression cassettes for monocots

MT1T2-PCR + pBUN411 et al.

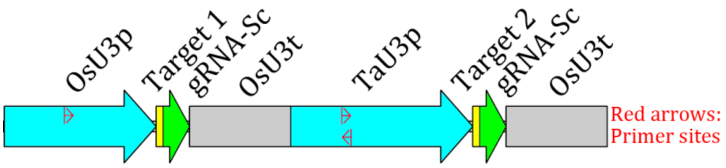

(OsU3p)-(Target-1)-(gRNA-Sc)-(OsU3t)-(TaU3p)-(Target-2)-(gRNA-Sc)-(OsU3t)

AGTAATTCATCCAGGTCTCCAAGTTCTAGGATTTTCAGAACTGCAACTTATTTTATCAAGGAATCTTTAAACATACGAACAGATCA
CTTAAAGTTCTTCTGAAGCAACTTAAAGTTATCAGGCATGCATGGATCTTGGAGGAATCAGATGTGCAGTCAGGGACCATAGCA
CAA**GACAGGCGTCTTCTACTGGTGCTAC**CAGCAAATGCTGGAAGCCGGGAACACTGGGTACGTTGGAAACCACGTGATGTGA
AGAAGTAAGATAAACTGTAGGAGAAAAGCATTTCGTAGTGGGCCATGAAGCCTTTCAGGACATGTATTGCAGTATGGGCCGGC
CCATTACGCAATTGGACGACAACAAAGACTAGTATTAGTACCACCTCGGCTATCCACATAGATCAAAGCTGATTTAAAAGAGTTG
TGCAGATGATCCGTGGCGNNNNNNNNNNNNNNNNNNNGTTTTAGAGCTAGAAATAGCAAGTTAAAATAAGG
CTAGTCCGTTATCAACTTGAAAAAGTGGCACCGAGTCGGTGCTTTTTTTTTTCGTTTTGCATTGAGTTTTCTC
CGTCGCATGTTTGCAGTTTTATTTTCCGTTTTGCATTGAAATTTCTCCGTCTCATGTTTGCAGCGTGTTCAAAA
AGTACGCAGCTGTATTTCACTTATTTACGGCGCCACATTTTCATGCCGTTTGTGCCAACTATCCCGAGCTAGTG
AATACAGCTTGGCTTCACACAACACTGGTGACCCGCTGACCTGCTCGTACCTCGTACCGTCGTACGGCACAG
CATTTGGAATTAAAGGGTGTGATCGATACTGCTTGCTGCTCATGAATCCAAACCACACGGAGTTCAAATTCCC
ACAGATTAAGGCTCGTCCGTCGCACAAGGTAATGTGTGAATATTATATCTGTCGTGCAAAATTGCCTGGCCTG
CACAATTGCTGTTATAGTTGGCGGCAGGGAGAGTTTTAACA**TT*GACTAGCGTGCTGATAATTTGTGAG***AAAT
AATAATTGACAAGTAGATACTGACATTTGAGAAGAGCTTCTGAACTGTTATTAGTAACAAAAATGGAAAGCTG
ATGCACGGAAAAAGGAAAGAAAAAGCCATACTTTTTTTTAGGTAGGAAAAGAAAAAGCCATACGAGACTGA
TGTCTCTCAGATGGGCCGGGATCTGTCTATCTAGCAGGCAGCAGCCCACCAACCTCACGGGCCAGCAATTAC
GAGTCCTTCTAAAAGCTCCCGCCGAGGGGCGCTGGCGCTGCTGTGCAGCAGCACGTCTAACATTAGTCCCA
CCTCGCCAGTTTACAGGGAGCAGAACCAGCTTATAAGCGGAGGCGCGGCACCAAGAAGCGNNNNNNNN
NNNNNNNNNNNGTTTTAGAGCTAGAAATAGCAAGTTAAAATAAGGCTAGTCCGTTATCAACTTGAAAAAGTGGCACCGAG
TCGGTGCTTTTTTTTTTCGTTTTGCATTGAGTTTTCTCCGTCGCATGTTTGCAGTTTTATTTTCCGTTTTGCATTGAAATTTCTCCG
TCTCATGTTTGCAGCGTGTTCAAAAAGTACGCAGCTGTATTTCACTTATTTACGGCGCCACATTTTCATGCCGTTTGTGCCAACTA
TCCCGAGCTAGTGAATACAGCTTGGCTTCACACAACACTGGTGACCCGCTGACCTGCTCGTACCTCGTACCGTCGTACGGCACA
GCATTTGGAATTAAAGGGTGTGATCGATACTGCTTGCTGCT

Notes:

1. Underlined letters come from binary vectors, while the others come from PCR fragments.
2. Red letters indicate primer sites.
3. Primer sequences are as follows:

Colony PCR primers (5'→3'):

OsU3p-F3: GACAGGCGTCTTCTACTGGTGCTAC

TaU3p-R: *CTCACAAATTATCAGCACGCTAGTC*

(OsU3p-F3 + TaU3p-R = 825 bp)

Sequencing primers (5'→3'):

OsU3p-F3: GACAGGCGTCTTCTACTGGTGCTAC

TaU3p-F2: TT*GACTAGCGTGCTGATAATTTGTG*
