# Supplementary material for: A CRISPR/Cas9 toolkit for multiplex genome editing in plants
Source: BMC Plant Biol. 2014 Nov 29;14:327. doi: 10.1186/s12870-014-0327-y (PMC4262988; doi:10.1186/s12870-014-0327-y)
Supplement: Additional file 4: Methods S3. — Golden Gate cloning method for the assembly of three or four gRNAs. [file 12870_2014_327_MOESM4_ESM.pdf]

Additional file 4

Methods S3

Golden Gate assembly method for the assembly of three or four gRNAs

Table of contents

Simplified protocol for the generation of CRISPR/Cas9 binary vectors 2
Nomenclature of PCR products and the primers for Golden Gate cloning 2
Sequence of DT1-PCR with Target 1 for dicots 4
Sequence of DT2T3-PCR2 with Targets 2 and 3 for dicots 5
Sequence of DT2-PCR with Target 2 for dicots 6
Sequence of DT3T4-PCR2 with Targets 3 and 4 for dicots 7
Sequence of three gRNA expression cassettes for dicots 8
Sequence of four gRNA expression cassettes for dicots 9
Sequence of MT1-PCR with Target 1 for monocots 10
Sequence of MT2T3-PCR2 with Targets 2 and 3 for monocots 11
Sequence of MT2-PCR with Target 2 for monocots 12
Sequence of MT3T4-PCR2 with Targets 3 and 4 for monocots 13
Sequence of three gRNA expression cassettes for monocots 14
Sequence of four gRNA expression cassettes for monocots 15

Simplified protocol

1. Manually search for 23-bp target sites (5'-N_{20}NGG-3') within exons of genomic DNA sequences of genes of interest, and then evaluate target specificities on the website of potential off-target finder (http://www.rgenome.net/cas-offinder/). Users can also search for target sites on the website of genome-wide prediction of plant CRISPR/Cas9 target sites (http://www.genome.arizona.edu/crispr/CRISPRsearch.html).
2. Design primers:
   a) Find names of PCR fragments and primers according to plant species (monocots or dicots) and gRNA numbers in Table S3-1.
   b) Find the sequences of the primers according to the names.
   c) Replace 19-nt N in the forward primers with your 19-nt target sequences in front of PAM (NGG), and 19-nt N in the reverse primers with reverse complement sequences of your 19-nt target sequences in front of PAM (NGG).
3. Carry out PCR reactions according to information provided under the sequences of the PCR fragments. Refer to Additional file 3: Methods S2.
4. Set up Golden Gate reactions. Refer to Additional file 3: Methods S2.
5. Transform *E.coli* competent cells, select positive clones on kanamycin LB agar plates.
6. Identify correct clones by colony PCR and verify them by sequencing.

Table S3-1 Nomenclature of PCR products and the primers for Golden Gate cloning

For dicots			
	DT1-PCR<br>DT1-BsF/DT1-F0<br>DT0-BsR2	DT2-PCR<br>DT2-BsF2/DT2-F0<br>DT0-BsR3	Number of gRNAs
DT2T3-PCR2<br>DT2-BsF2/DT2-F0<br>DT3-R0/DT3-BsR	+	-	3
DT3T4-PCR2<br>DT3-BsF3/DT3-F0<br>DT4-R0/DT4-BsR	+	+	4
**For monocots**			
	MT1-PCR<br>MT1-BsF/MT1-F0<br>MT0-BsR2	MT2-PCR<br>MT2-BsF2/MT2-F0<br>MT0-BsR3	Number of gRNAs
MT2T3-PCR2<br>MT2-BsF2/MT2-F0<br>MT3-R0/MT3-BsR	+	-	3
MT3T4-PCR2<br>MT3-BsF3/MT3-F0<br>MT4-R0/MT4-BsR	+	+	4

Notes:

1. The primer names are under the PCR names and highlighted.
2. "+" or "- " indicates whether or not the PCR fragment in the column is mixed with the PCR fragment in the row of Column 1 for Golden Gate cloning.
3. When using more than two PCR primers, dilute the F0/R0 primers to 20 times of the F/R primers.
4. BsF/BsR, forward/reverse primer with *Bsa*I site. The BsR2 and BsF2 have compatible *Bsa*I sites, so do the BsR3 and BsF3. The BsR2 and BsR3 are universal primers and can be used repeatedly. F0 represents no target sequence added to the primer.

Sequence of DT1-PCR with Target 1 for dicots

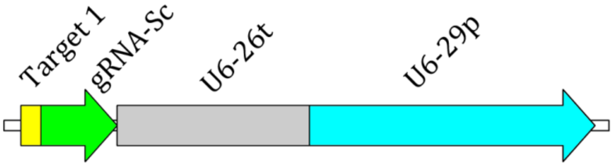

(Target-1)-(gRNA-Sc)-(U6-26t)-(U6-29p)

ATATATGGTCTCG**ATTG**NNNNNNNNNNNNNNNNNNNGTTTTAGAGCTAGAAATAGCAAGTTAAAAT
AAGGCTAGTCCGTTATCAACTTGAAAAAGTGGCACCGAGTCGGTGCTTTTTTTTGCAAAATTTTCCAGATCG
ATTTCTTCTTCCTCTGTTCTTCGGCGTTCAATTTCTGGGGTTTTCTCTTCGTTTTCTGTAACTGAAACCTAAAAT
TTGACCTAAAAAAAATCTCAAATAATATGATTCAGTGGTTTTGTACTTTTCAGTTAGTTGAGTTTTGCAGTTCC
GATGAGATAAACCAATATTAATCCAAACTACTGCAGCCTGACAGACAAATGAGGATGCAAACAATTTTAAAG
TTTATCTAACGCTAGCTGTTTTGTTTCTTCTCTCTGGTGCACCAACGACGGCGTTTTCTCAATCATAAAGAG
GCTTGTTTTACTTAAGGCCAATAATGTTGATGGATCGAAAGAAGAGGGCTTTTAATAAACGAGCCCGTTTA
AGCTGTAAACGATGTCAAAAACATCCCACATCGTTCAGTTGAAAATAGAAGCTCTGTTTATATATTGGTAG
AGTCGACTAAG**AGAT**TGAGACCAATAATAT

Primers:

DT1-BsF: ATATATGGTCTCG**ATTG**NNNNNNNNNNNNNNNNNNNGTT

DT1-F0: TGNNNNNNNNNNNNNNNNNNNGTTTTAGAGCTAGAAATAGC

DT0-BsR2: 5' ATATTATTGGTCTCA**ATCT**CTTAGTCGACTCTACCAAT

Template: pCBC-DT1T2

Length: 603-bp

Notes:

1. The 19-nt N in primers represent any 19-nt target sequence (forward primers) or reverse complement sequence of any 19-nt target sequence (reverse primers) in front of PAM (NGG).
2. Enlarged and boxed letters indicate 5' protruding ends produced by *Bsa*I digestion.
3. When using DT1-BsF/DT1-F0/DT0-BsR2 three-primer mixture, dilute DT1-F0 to 20 times of DT1-BsF or DT0-BsR2.

Sequence of DT2T3-PCR2 with Targets 2 and 3 for dicots

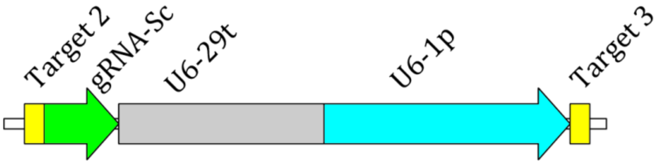

(Target-2)-(gRNA-Sc)-(U6-29t)-(U6-1p)-(Target-3)

ATATTATTGGTCTCA**AGAT**TGNNNNNNNNNNNNNNNNNNNGTTTTAGAGCTAGAAATAGCAAGTT
AAAATAAGGCTAGTCCGTTATCAACTTGAAAAAGTGGCACCGAGTCGGTGCTTTTTTTGGATAGAATTTCCC
AGCTTTTTTGCGTGTTTCAGCTCTCATGATCCTTGGCCAATGGGTGTAGTAAATTTTCTGCACATTCATTGG
ATGGAAAATAATGGTTTTAGCTTTAGGGAATAAGAAAAGTGTATAGGAAGGGGATTTTTGTACAATCACA
TTTGAATTAGGTCTTTGAAATGACAGGGAATGAGGACATATGATGAGACGGTCATTGTTTTAGTTCCACCA
CGATTATATTTGAAATTTACGTGAGTGTGAGTGAGACTTGCATAAGAAAATAAAATCTTTAGTTGGGAAA
AAATTCAATAATATAAATGGGCTTGAGAAGGAAGCGAGGGATAGGCCTTTTTCTAAAATAGGCCCATTTA
AGCTATTAACAATCTTCAAAAGTACCACAGCGCTTAGGTAAAGAAAGCAGCTGAGTTTATATATGGTTAG
AGACGAAGTAGTGATTGNNNNNNNNNNNNNNNNNNN**GTTT**CGAGACCAATAAT

Primers:

DT2-BsF2: 5' ATATTATTGGTCTCA**AGAT**TGNNNNNNNNNNNNNNNNNNNGTT 3'

DT2-F0: 5' TGNNNNNNNNNNNNNNNNNNNGTTTTAGAGCTAGAAATAGC 3'

DT3-R0: 5' AACNNNNNNNNNNNNNNNNNNNCAATCACTACTTCGTCTCTAACCAT 3'

DT3-BsR: 5' ATTATTGGTCTCG**AAAC**NNNNNNNNNNNNNNNNNNNC 3'

Template: pCBC-DT2T3

Length: 613-bp

Notes:

1. The 19-nt N in primers represent any 19-nt target sequence (forward primers) or reverse complement sequence of any 19-nt target sequence (reverse primers) in front of PAM (NGG).
2. Enlarged and boxed letters indicate 5' protruding ends produced by *Bsa*I digestion.
3. When using DT2-BsF2/DT2-F0/DT3-R0/DT3-BsR four-primer mixture, dilute DT2-F0 and DT3-R0 to 20 times of DT2-BsF2 or DT3-BsR.

Sequence of DT2-PCR with Target 2 for dicots

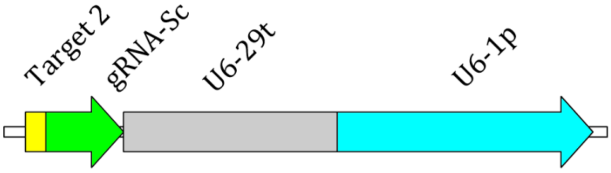

(Target-2)-(gRNA-Sc)-(U6-29t)-(U6-1p)

ATATTATTGGTCTCA**AGAT**TGNNNNNNNNNNNNNNNNNNNGTTTTAGAGCTAGAAATAGCAAGTT
AAAATAAGGCTAGTCCGTTATCAACTTGAAAAAGTGGCACCGAGTCGGTGCTTTTTTTGGATAGAATTTCCC
AGCTTTTTTGCGTGTTTCAGCTCTCATGATCCTTGGCCAATGGGTGTAGTAAATTTTCTGCACATTCATTGG
ATGGAAAATAATGGTTTTAGCTTTAGGGAATAAGAAAAGTGTATAGGAAGGGGATTTTTGTACAATCACA
TTTGAATTAGGTCTTTGAAATGACAGGGAATGAGGACATATGATGAGACGGTCATTGTTTTAGTTCCACCA
CGATTATATTTGAAATTTACGTGAGTGTGAGTGAGACTTGCATAAGAAAATAAAATCTTTAGTTGGGAAA
AAATTCAATAATATAAATGGGCTTGAGAAGGAAGCGAGGGATAGGCCTTTTTCTAAAATAGGCCCATTTA
AGCTATTAACAATCTTCAAAAGTACCACAGCGCTTAGGTAAAGAAAGCAGCTGAGTTTATATATGGTTAG
AGACGAAGTA**GTGA**TGAGACCAATAATAT

Primers:

DT2-BsF2: 5' ATATTATTGGTCTCA**AGAT**TGNNNNNNNNNNNNNNNNNNNGTT 3'

DT2-F0: 5' TGNNNNNNNNNNNNNNNNNNNGTTTTAGAGCTAGAAATAGC 3'

DT0-BsR3: ATATTATTGGTCTCA**TCAC**TACTTCGTCTCTAACCAT

Template: pCBC-DT2T3

Length: 589-bp

Notes:

1. The 19-nt N in primers represent any 19-nt target sequence (forward primers) or reverse complement sequence of any 19-nt target sequence (reverse primers) in front of PAM (NGG).
2. Enlarged and boxed letters indicate 5' protruding ends produced by *Bsa*I digestion.
3. When using DT2-BsF2/DT2-F0/DT0-BsR3 three-primer mixture, dilute DT2-F0 to 20 times of DT2-BsF2 or DT0-BsR3.

Sequence of DT3T4-PCR2 with Targets 3 and 4 for dicots

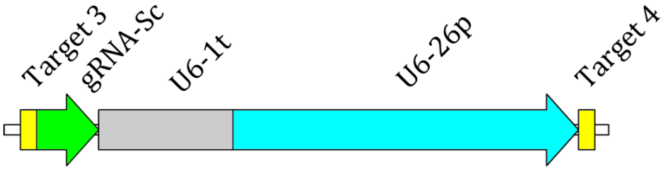

(Target-3)-(gRNA-Sc)-(U61-t)-(U6-26p)-(Target-4)

ATATTATTGGTCTCA**GTGA**TTGNNNNNNNNNNNNNNNNNNNGTTTTAGAGCTAGAAATAGCAAGT
TAAAATAAGGCTAGTCCGTTATCAACTTGAAAAAGTGGCACCGAGTCGGTGCTTTTTTTTGGCAAAAATTTTC
AGATTTTTTCTTCATCTGTAGATTTCTGGGTTTTTTTTTCCGTTTCGTGAATCATAAGTGAAGTTTTGGATGC
AAATCTGCGCGAAAAAAGTTGGACCTGCAATGAGCTTATTTAGATAGCTAAGACAAAGTGATTGGTCCGT
TCGACTTGCCTTCCGCACAATACATCATTTCTTCTTAGCTTTTTTTCTTCTTCTTCGTTCATACAGTTTTTTTTTG
TTTATCAGCTTACATTTTCTTGAACCGTAGCTTTCGTTTTCTTCTTTTTAACTTTCCATTCGGAGTTTTTGTATCT
TGTTTCATAGTTTGTCCCAGGATTAGAATGATTAGGCATCGAACCTTCAAGAATTTGATTGAATAAAACATCTT
CATTCTTAAGATATGAAGATAATCTTCAAAAGGCCCCTGGGAATCTGAAAGAAGAGAAGCAGGCCCATTTATA
TGGGAAAGAACAATAGTATTTCTTATATAGGCCCATTTAAGTTGAAAACAATCTTCAAAAGTCCCACATCGCTT
AGATAAGAAAACGAAGCTGAGTTTATATACAGCTAGAGTCGAAGTAGTGATTGNNNNNNNNNNNNNNNN
NNN**GTTT**AGAGACCAATAAT

Primers:

DT3-BsF3: 5' ATATTATTGGTCTCA**GTGA**TTGNNNNNNNNNNNNNNNNNNNGTT

DT3-F0: 5' TGNNNNNNNNNNNNNNNNNNNGTTTTAGAGCTAGAAATAGC 3'

DT4-R0: 5' AACNNNNNNNNNNNNNNNNNNNCAATCACTACTTCGACTCTAGCTGTAT

DT4-BsR: 5' ATTATTGGTCTCT**AAAC**NNNNNNNNNNNNNNNNNNN 3'

Template: pCBC-DT3T4

Length: 743-bp

Notes:

1. The 19-nt N in primers represent any 19-nt target sequence (forward primers) or reverse complement sequence of any 19-nt target sequence (reverse primers) in front of PAM (NGG).
2. Enlarged and boxed letters indicate 5' protruding ends produced by *Bsa*I digestion.
3. When using DT3-BsF3/DT3-F0/DT4-R0/DT4-BsR four-primer mixture, dilute DT3-F0 and DT4-R0 to 20 times of DT3-BsF3 or DT4-BsR.

Sequence of three gRNA expression cassettes for dicots

DT1-PCR + DT2T3-PCR2 + pHSN401 et al.

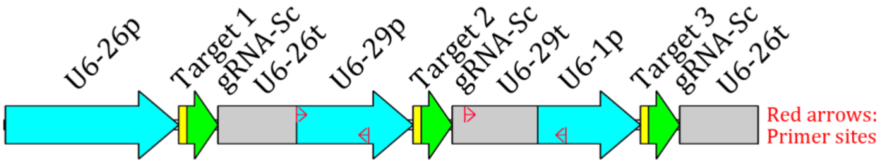

(U6-26p)-(Target-1)-(gRNA-Sc)-(U6-26t)-(U6-29p)-(Target-2)-(gRNA-Sc)-(U6-29t)-
(U6-1p)-(Target-3)-(gRNA-Sc)-(U6-26t)

CGACTTGCCTTCCGCACAATACATCATTTCTTCTTAGCTTTTTTTCTTCTTCTTCGTTCATACAGTTTTTTTTTGTTTATCAGCTTAC
ATTTTCTTGAACCGTAGCTTTCGTTTTCTTCTTTTTAACTTTCCATTCGGAGTTTTTGTATCTTGTTTCATAGTTTGTCCCAGGATTA
GAATGATTAGGCATCGAACCTTCAAGAATTTGATTGAATAAAACATCTTCATTCTTAAGATATGAAGATAATCTTCAAAAGGCCCC
TGGGAATCTGAAAGAAGAGAAGCAGGCCCATTTATATGGGAAAGAACAATAGTATTTCTTATATAGGCCCATTTAAGTTGAAAAC
AATCTTCAAAAGTCCCACATCGCTTAGATAAGAAAACGAAGCTGAGTTTATATACAGCTAGAGTCGAAGTAGTGATTGNN
NNNNNNNNNNNNNNNNNGTTTTAGAGCTAGAAATAGCAAGTTAAAATAAGGCTAGTCCGTTATCAACTTGAAAAAGTGGCA
CCGAGTCGGTGCTTTTTTTTGCAAAATTTCCAGATCGATTTCTTCTTCCTCTGTTCTTCGGCGTTCAATTTCTGGGGTTTTCTCTT
CGTTTTCTGTAACTGAAACCTAAAATTTGACCTAAAAAAAATCTCAAATAATATGATTCAGTGGTTTTGTACTTTTCAGTTAGTTGA
GTTTTGCAGTTCCGATGAGATAAACCAATA**TTAATCCAAACTACTGCAGCCTGAC**AGACAAATGAGGATGCAAACAATTTTAAA
GTTTATCTAACGCTAGCTGTTTTGTTTCTTCTCTCTGGTGCACCAACGACGGCGTTTTCTCAATCATAAAGAGGCTTGTTTTACT
TAAGGCCAATAAT**GTTGATGGATCGAAAGAAGAGGGCT**TTTAATAAACGAGCCCGTTTAAGCTGTAAACGATGTCAAAAAC
ATCCCACATCGTTCAGTTGAAAATAGAAGCTCTGTTTATATATTGGTAGAGTCGACTAAGAGATTGNNNNNNNNNNN
NNNNNNNNGTTTTAGAGCTAGAAATAGCAAGTTAAAATAAGGCTAGTCCGTTATCAACTTGAAAAAGTGGCACCGAGTCGGT
GCTTTTTTTGGATAGAATTTCCCAGCTTTTTTG**CGTGTTTCAGCTCTCATGATCCTTG**GCCAATGGGTGTAGTAAATTTTCTGC
ACATTCATTGGATGGAAAATAATGGTTTTAGCTTTAGGGAATAAGAAAAGTGTATAGGAAGGGGATTTTTGTACAATCACAT
TTGAATTAGGTCTTTGAAATGACAGGGAATGAGGACATATGATGAGACGGTCATTGTTTTAGTTCCACCACGATTATATTTGA
AATTTA**CGTGAGTGTGAGTGAGACTTGCATA**AGAAAATAAAATCTTTAGTTGGGAAAAAATTCAATAATATAAATGGGCTTG
AGAAGGAAGCGAGGGATAGGCCTTTTTCTAAAATAGGCCCATTTAAGCTATTAACAATCTTCAAAAGTACCACAGCGCTTAG
GTAAAGAAAGCAGCTGAGTTTATATATGGTTAGAGACGAAGTAGTGATTGNNNNNNNNNNNNNNNNNNNGTTTTA
GAGCTAGAAATAGCAAGTTAAAATAAGGCTAGTCCGTTATCAACTTGAAAAAGTGGCACCGAGTCGGTGCTTTTTTTTGCAAAA
TTTTCCAGATCGATTTCTTCTTCCTCTGTTCTTCGGCGTTCAATTTCTGGGGTTTTCTCTTCGTTTTCTGTAACTGAAACCTAAAAT
TTGACCTAAAAAAAATCTCAAATAATATGATTCAGTGGTTTTGTACTTTTCAGTTAGTTGAGTTTTGCAGTTCCGATGAGATAAAC
CAATA

Notes:

1. Underlined letters come from binary vectors, while the others come from PCR fragments.
2. Red letters indicate primer sites.
3. Enlarged and boxed letters indicate 5' protruding ends produced by *Bsa*I digestion.
4. Primer sequences are as follows:

Colony PCR primers (5'→3')
U6-29p-F: TTAATCCAAACTACTGCAGCCTGAC
U6-1p-R: TATGCAAGTCTCACTCACACTCACG
(U6-29p-F + U6-1p-R = 659 bp)

Sequencing primers (5'→3'):
U6-29p-F: TTAATCCAAACTACTGCAGCCTGAC
U6-29p-R: AGCCCTCTTCTTTCGATCCATCAAC
U6-29t-F: CGTGTTTCAGCTCTCATGATCCTTG

Sequence of four gRNA expression cassettes for dicots

DT1-PCR + DT2-PCR + DT3T4-PCR2 + pHSN401 et al.

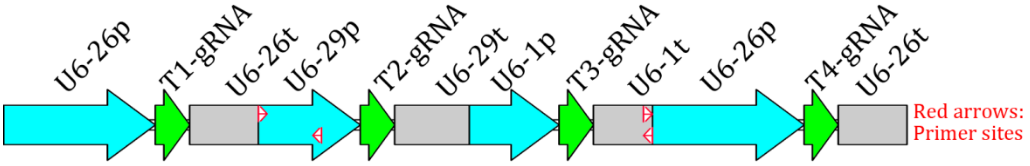

(U6-26p)-(Target-1)-(gRNA-Sc)-(U6-26t)-(U6-29p)-(Target-2)-(gRNA-Sc)-(U6-29t)-
(U6-1p)-(Target-3)- (gRNA-Sc)-(U6-1t)-(U6-26p)-(Target-4)-(gRNA-Sc)-(U6-26t)

CGACTTGCCTTCCGCACAATACATCATTTCTTCTTAGCTTTTTTTCTTCTTCTTCGTTCATACAGTTTTTTTTTGTTTATCAGCTTAC
ATTTTCTTGAACCGTAGCTTTCGTTTTCTTCTTTTTAACTTTCCATTCGGAGTTTTTGTATCTTGTTTCATAGTTTGTCCCAGGATTA
GAATGATTAGGCATCGAACCTTCAAGAATTTGATTGAATAAAACATCTTCATTCTTAAGATATGAAGATAATCTTCAAAAGGCCCC
TGGGAATCTGAAAGAAGAGAAGCAGGCCCATTTATATGGGAAAGAACAATAGTATTTCTTATATAGGCCCATTTAAGTTGAAAAC
AATCTTCAAAAGTCCCACATCGCTTAGATAAGAAAACGAAGCTGAGTTTATATACAGCTAGAGTCGAAGTAGTG**ATTG**NN
NNNNNNNNNNNNNNNNNNGTTTTAGAGCTAGAAATAGCAAGTTAAAATAAGGCTAGTCCGTTATCAACTTGAAAAAGTGGCA
CCGAGTCGGTGCTTTTTTTTGCAAAATTTTCCAGATCGATTTCTTCTTCCTCTGTTCTTCGGCGTTCAATTTCTGGGGTTTTCTCTT
CGTTTTCTGTAACTGAAACCTAAAATTTGACCTAAAAAAAAATCTCAAATAATATGATTCAGTGGTTTTGTACTTTTCAGTTAGTTGA
GTTTTGCAGTTCCGATGAGATAAACCAATA**TTAATCCAAACTACTGCAGCCTGAC**AGACAAATGAGGATGCAAACAATTTTAAA
GTTTATCTAACGCTAGCTGTTTTGTTTCTTCTCTCTGGTGCACCAACGACGGCGTTTTCTCAATCATAAAGAGGCTTGTTTTACT
TAAGGCCAATAAT**GTTGATGGATCGAAAGAAGAGGGCT**TTTAATAAACGAGCCCGTTTAAGCTGTAAACGATGTCAAAAAC
ATCCCACATCGTTCAGTTGAAAATAGAAGCTCTGTTTATATATTGGTAGAGTCGACTAAG**AGAT**TGNNNNNNNNNNN
NNNNNNNNGTTTTAGAGCTAGAAATAGCAAGTTAAAATAAGGCTAGTCCGTTATCAACTTGAAAAAGTGGCACCGAGTCGGT
GCTTTTTTTGGATAGAATTTCCCAGCTTTTTTGCGTGTTTCAGCTCTCATGATCCTTGGCCAATGGGTGTAGTAAATTTTCTGCA
CATTCATTGGATGGAAAATAATGGTTTTAGCTTTAGGGAATAAGAAAAGTGTATAGGAAGGGGATTTTTGTACAATCACATT
TGAATTAGGTCTTTGAAATGACAGGGAATGAGGACATATGATGAGACGGTCATTGTTTTAGTTCCACCACGATTATATTTGA
AATTTA**CGTGAGTGTGAGTGAGACTTGCATA**AGAAAATAAAATCTTTAGTTGGGAAAAAATTCAATAATATAAATGGGCTTG
AGAAGGAAGCGAGGGATAGGCCTTTTTCTAAAATAGGCCCATTTAAGCTATTAACAATCTTCAAAAGTACCACAGCGCTTAG
GTAAAGAAAGCAGCTGAGTTTATATATGGTTAGAGACGAAGTA**GTGA**TTGNNNNNNNNNNNNNNNNNNNGTTTT
AGAGCTAGAAATAGCAAGTTAAAATAAGGCTAGTCCGTTATCAACTTGAAAAAGTGGCACCGAGTCGGTGCTTTTTTTTGGCAA
AAATTTTCAGATTTTTTCTTCATCTGTAGATTTCTGGGTTTTTTTTTCCGTTTCGTGAATCATAAGTGAAGTTTTGGATGCAAAT
CTGCGCGAAAAAAGTTGGACCTGCAATGAGCTTATTTAGATA**GCTAAGACAAAGTGATTGGTCCGTT**CGACTTGCCTTCCGC
ACAATACATCATTTCTTCTTAGCTTTTTTTCTTCTTCTTCGTTCATACAGTTTTTTTTTGTTTATCAGCTTACATTTTCTTGAACCGTA
GCTTTCGTTTTCTTCTTTTTAACTTTCCATTCGGAGTTTTTGTATCTTGTTTCATAGTTTGTCCCAGGATTAGAATGATTAGGCATC
GAACCTTCAAGAATTTGATTGAATAAAACATCTTCATTCTTAAGATATGAAGATAATCTTCAAAAGGCCCCTGGGAATCTGAAAG
AAGAGAAGCAGGCCCATTTATATGGGAAAGAACAATAGTATTTCTTATATAGGCCCATTTAAGTTGAAAACAATCTTCAAAAGTC
CCACATCGCTTAGATAAGAAAACGAAGCTGAGTTTATATACAGCTAGAGTCGAAGTAGTGATTGNNNNNNNNNNNNNNNNN
NN**GTTT**TAGAGCTAGAAATAGCAAGTTAAAATAAGGCTAGTCCGTTATCAACTTGAAAAAGTGGCACCGAGTCGGTGCT
TTTTTTTGCAAAATTTTCCAGATCGATTTCTTCTTCCTCTGTTCTTCGGCGTTCAATTTCTGGGGTTTTCTCTTCGTTTTCTGTAACT
GAAACCTAAAATTTGACCTAAAAAAAAATCTCAAATAATATGATTCAGTGGTTTTGTACTTTTCAGTTAGTTGAGTTTTGCAGTTCC
GATGAGATAAACCAATA

Notes:

1. Underlined letters come from binary vectors, while the others come from PCR fragments.
2. Red letters indicate primer sites.
3. Enlarged and boxed letters indicate 5' protruding ends produced by *Bsa*I digestion.
4. Primer sequences are as follows:

Colony PCR primers (5'→3'):
U6-29p-F: TTAATCCAAACTACTGCAGCCTGAC'
U6-1t-R: AACGGACCAATCACTTTGTCTTAGC'
(U6-29p-F + U6-1t-R = 1.1 kb)

Sequencing primers (5'→3'):
U6-29p-F: TTAATCCAAACTACTGCAGCCTGAC
U6-29p-R: AGCCCTCTTCTTTCGATCCATCAAC
U6-1t-F: GCTAAGACAAAGTGATTGGTCCGTT
U6-1t-R: AACGGACCAATCACTTTGTCTTAGC

Sequence of MT1-PCR with Target 1 for monocots

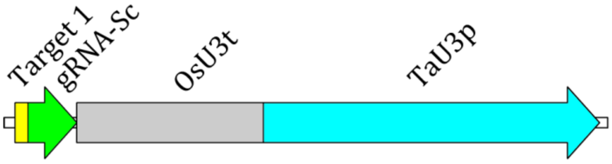

(Target-1)-(gRNA-Sc)-(OsU3t)-(TaU3p)

ATATATGGTCTCTGGCGNNNNNNNNNNNNNNNNNNNGTTTTAGAGCTAGAAATAGCAAGTTAAAA
TAAGGCTAGTCCGTTATCAACTTGAAAAAGTGGCACCGAGTCGGTGCTTTTTTTTTTCGTTTTGCATTGAGTT
TTCTCCGTCGCATGTTTGCAGTTTTATTTTCCGTTTTGCATTGAAATTTCTCCGTCTCATGTTTGCAGCGTGTTC
AAAAAGTACGCAGCTGTATTTCACTTATTTACGGCGCCACATTTTCATGCCGTTTGTGCCAACTATCCCGAGCT
AGTGAATACAGCTTGGCTTCACACAACACTGGTGACCCGCTGACCTGCTCGTACCTCGTACCGTCGTACGGC
ACAGCATTTGGAATTAAAGGGTGTGATCGATACTGCTTGCTGCTCATGAATCCAAACCACACGGAGTTCAAA
TTCCCACAGATTAAGGCTCGTCCGTCGCACAAGGTAATGTGTGAATATTATATCTGTCGTGCAAAATTGCCTG
GCCTGCACAATTGCTGTTATAGTTGGCGGCAGGGAGAGTTTTAACATTGACTAGCGTGCTGATAATTTGTGA
GAAATAATAATTGACAAGTAGATACTGACATTTGAGAAGAGCTTCTGAACTGTTATTAGTAACAAAAATGGAA
AGCTGATGCACGGAAAAAGGAAAGAAAAAGCCATACTTTTTTTTAGGTAGGAAAAGAAAAAGCCATACGA
GACTGATGTCTCTCAGATGGGCCGGGATCTGTCTATCTAGCAGGCAGCAGCCCACCAACCTCACGGGCCAG
CAATTACGAGTCCTTCTAAAAGCTCCCGCCGAGGGGCGCTGGCGCTGCTGTGCAGCAGCACGTCTAACATTA
GTCCCACCTCGCCAGTTTACAGGGAGCAGAACCAGCTTATAAGCGGAGGCGCGGCACCAAGAAGC
AGAGACCAATAAT

Primers:

MT1-BsF: ATATATGGTCTCTGGCGNNNNNNNNNNNNNNNNNNNGTT

MT1-F0: TGNNNNNNNNNNNNNNNNNNNGTTTTAGAGCTAGAAATAGC

MT0-BsR2: ATTATTGGTCTCTGCTTCTTGGTGCCGC

Template: pCBC-MT1T2

Length: 940-bp

Notes:

1. The 19-nt N in primers represent any 19-nt target sequence (forward primers) or reverse complement sequence of any 19-nt target sequence (reverse primers) in front of PAM (NGG).
2. Enlarged and boxed letters indicate 5' protruding ends produced by *Bsa*I digestion.
3. When using MT1-BsF/MT1-F0/MT0-BsR2 three-primer mixture, dilute MT1-F0 to 20 times of MT1-BsF or MT0-BsR2.

Sequence of MT2T3-PCR2 with Targets 2 and 3 for monocots

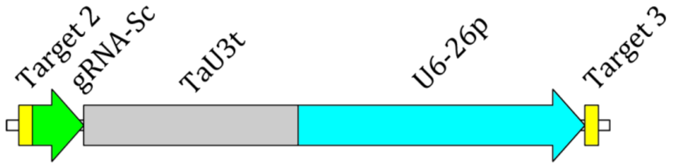

(Target-2)-(gRNA-Sc)-(TaU3t)-(U6-26p)-(Target-3)

ATATATGGTCTCT**AAGC**GNNNNNNNNNNNNNNNNNNNGTTTTAGAGCTAGAAATAGCAAGTTAAA
ATAAGGCTAGTCCGTTATCAACTTGAAAAAGTGGCACCGAGTCGGTGCTTTTTTTTTTGTCCTTCTGTTTTTTT
AGTCAGTCTCTTTTTTCAGAAGTACAACATCTTTTTTTTGTCCTTCTGTTTTTTTAGTCAGTCTCTTTTTTCAGA
AGTACAACATCTTTTTTTTGTCCTTCTGTTTTTTTAGTCAGTCTCTTTTTTCAGAAGTACAACATCTTTTTTTTG
TCCTTCTGTTTTTTTAGTCAGTCTCTTTTTTCAGAAGTACTCTATGATGTATCGTTCTGGGAAATGTCTGTCTGT
CTACAACCCCATAATCTATATTTGCAATCACACATCTAATATTCTCTGTGACAAGACAGCCGAACACGACTTGC
CTTCCGCACAATACATCATTTCTTCTTAGCTTTTTTTCTTCTTCTTCGTTCATACAGTTTTTTTTTGTTTATCAGC
TTACATTTTCTTGAACCGTAGCTTTCGTTTTCTTCTTTTTAACTTTCCATTCGGAGTTTTTGTATCTTGTTTCATA
GTTTGTCCCAGGATTAGAATGATTAGGCATCGAACCTTCAAGAATTTGATTGAATAAAACATCTTCATTCTTAA
GATATGAAGATAATCTTCAAAAGGCCCCTGGGAATCTGAAAGAAGAGAAGCAGGCCCATTTATATGGGAAA
GAACAATAGTATTTCTTATATAGGCCCATTTAAGTTGAAAACAATCTTCAAAAGTCCCACATCGCTTAGATAAG
AAAACGAAGCTGAGTTTATATACAGCTAGAGTCGAAGTAGTGATTGNNNNNNNNNNNNNNNNNNN**G**
TTTAGAGACCAATAAT

Primers:

MT2-BsF2: ATATATGGTCTCT**AAGC**GNNNNNNNNNNNNNNNNNNNGTT

MT2-F0: TGNNNNNNNNNNNNNNNNNNNGTTTTAGAGCTAGAAATAGC

MT3-R0: AACNNNNNNNNNNNNNNNNNNNCAATCACTACTTCGACTCTAGC

MT3-BsR: ATTATTGGTCTCT**AAAC**NNNNNNNNNNNNNNNNNNNC

Template: pCBC-MT2T3

Length: 891-bp

Notes:

1. The 19-nt N in primers represent any 19-nt target sequence (forward primers) or reverse complement sequence of any 19-nt target sequence (reverse primers) in front of PAM (NGG).
2. Enlarged and boxed letters indicate 5' protruding ends produced by *Bsa*I digestion.
3. When using MT2-BsF2/MT2-F0/MT3-R0/MT3-BsR four-primer mixture, dilute MT2-F0 and MT3-R0 to 20 times of MT2-BsF2 or MT3-BsR.

Sequence of MT2-PCR with Target 2 for monocots

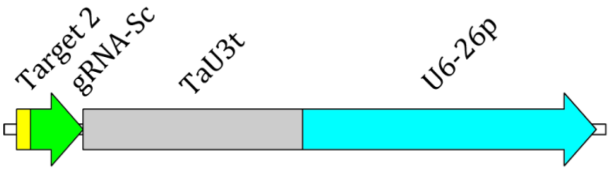

(Target-2)-(gRNA-Sc)-(TaU3t)-(U6-26p)

ATATATGGTCTCTAAGCGNNNNNNNNNNNNNNNNNNNNGTTTTAGAGCTAGAAATAGCAAGTTAAA
ATAAGGCTAGTCCGTTATCAACTTGAAAAAGTGGCACCGAGTCGGTGCTTTTTTTTTTGTCCTTCTGTTTTTTT
AGTCAGTCTCTTTTTTCAGAAGTACAACATCTTTTTTTTGTCCTTCTGTTTTTTTAGTCAGTCTCTTTTTTCAGA
AGTACAACATCTTTTTTTTGTCCTTCTGTTTTTTTAGTCAGTCTCTTTTTTCAGAAGTACAACATCTTTTTTTTG
TCCTTCTGTTTTTTTAGTCAGTCTCTTTTTTCAGAAGTACTCTATGATGTATCGTTCTGGGAAATGTCTGTCTGT
CTACAACCCCATAATCTATATTTGCAATCACACATCTAATATTCTCTGTGACAAGACAGCCGAACACGACTTGC
CTTCCGCACAATACATCATTTCTTCTTAGCTTTTTTTCTTCTTCTTCGTTCATACAGTTTTTTTTTGTTTATCAGC
TTACATTTTCTTGAACCGTAGCTTTCGTTTTCTTCTTTTTAACTTTCCATTCGGAGTTTTTGTATCTTGTTTCATA
GTTTGTCCCAGGATTAGAATGATTAGGCATCGAACCTTCAAGAATTTGATTGAATAAAACATCTTCATTCTTAA
GATATGAAGATAATCTTCAAAAGGCCCCTGGGAATCTGAAAGAAGAGAAGCAGGCCCATTTATATGGGAAA
GAACAATAGTATTTCTTATATAGGCCCATTTAAGTTGAAAACAATCTTCAAAAGTCCCACATCGCTTAGATAAG
AAAACGAAGCTGAGTTTATATACAGCTAGAGTCGAAGTAGTGATTGAGAGACCAATAAT

Primers:

MT2-BsF2: ATATATGGTCTCTAAGCGNNNNNNNNNNNNNNNNNNNNGTT

MT2-F0: TGNNNNNNNNNNNNNNNNNNNNGTTTTAGAGCTAGAAATAGC

MT0-BsR3: ATTATTGGTCTCTCAATCACTACTTCGACTCTAGC

Template: pCBC-MT2T3

Length: 868-bp

Notes:

1. The 19-nt N in primers represent any 19-nt target sequence (forward primers) or reverse complement sequence of any 19-nt target sequence (reverse primers) in front of PAM (NGG).
2. Enlarged and boxed letters indicate 5' protruding ends produced by *Bsa*I digestion.
3. When using MT2-BsF2/MT2-F0/MT0-BsR3 three-primer mixture, dilute MT2-F0 to 20 times of MT2-BsF2 or MT0-BsR3.

Sequence of MT3T4-PCR2 with Targets 3 and 4 for monocots

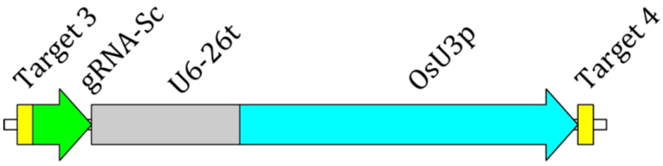

(Target-3)-(gRNA-Sc)-(U6-26t)-(OsU3p)-(Target-4)

ATATATGGTCTCTATTGNNNNNNNNNNNNNNNNNNNGTTTTAGAGCTAGAAATAGCAAGTTAAAAT
AAGGCTAGTCCGTTATCAACTTGAAAAAGTGGCACCGAGTCGGTGCTTTTTTTTGCAAAATTTTCCAGATCG
ATTTCTTCTTCCTCTGTTCTTCGGCGTTCAATTTCTGGGGTTTTCTCTTCGTTTTCTGTAACTGAAACCTAAAAT
TTGACCTAAAAAAAATCTCAAATAATATGATTCAGTGGTTTTGTACTTTTCAGTTAGTTGAGTTTTGCAGTTCC
GATGAGATAAACCAATAAGTAATTCATCCAGGTCACCAAGTTCTAGGATTTTCAGAACTGCAACTTATTTTATC
AAGGAATCTTTAAACATACGAACAGATCACTTAAAGTTCTTCTGAAGCAACTTAAAGTTATCAGGCATGCATG
GATCTTGGAGGAATCAGATGTGCAGTCAGGGACCATAGCACAAGACAGGCGTCTTCTACTGGTGCTACCAG
CAAATGCTGGAAGCCGGGAACACTGGGTACGTTGGAAACCACGTGATGTGAAGAAGTAAGATAAACTGTAG
GAGAAAAGCATTTCGTAGTGGGCCATGAAGCCTTTCAGGACATGTATTGCAGTATGGGCCGGCCCATTACGC
AATTGGACGACAACAAAGACTAGTATTAGTACCACCTCGGCTATCCACATAGATCAAAGCTGATTTAAAAGAG
TTGTGCAGATGATCCGTGGCGNNNNNNNNNNNNNNNNNNNGTTTAGAGACCAATAAT

Primers:

MT3-BsF3: ATATATGGTCTCTATTGNNNNNNNNNNNNNNNNNNNGTT

MT3-F0: TGNNNNNNNNNNNNNNNNNNNGTTTTAGAGCTAGAAATAGC

MT4-R0: AACNNNNNNNNNNNNNNNNNNNCGCCACGGATCATCTGCACAACT 3'

MT4-BsR: ATTATTGGTCTCTAAACNNNNNNNNNNNNNNNNNNNC

Template: pCBC-MT3T4

Length: 778-bp

Notes:

1. The 19-nt N in primers represent any 19-nt target sequence (forward primers) or reverse complement sequence of any 19-nt target sequence (reverse primers) in front of PAM (NGG).
2. Enlarged and boxed letters indicate 5' protruding ends produced by *Bsa*I digestion.
3. When using MT3-BsF3/MT3-F0/MT4-R0/MT4-BsR four-primer mixture, dilute MT3-F0 and MT4-R0 to 20 times of MT3-BsF3 or MT4-BsR.

Sequence of three gRNA expression cassettes for monocots

MT1-PCR + MT2T3-PCR2 + pBUN411 et al.

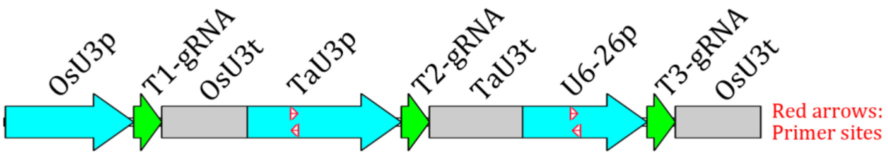

(OsU3p)-(Target-1)-(gRNA-Sc)-(OsU3t)-(TaU3p)-(Target-2)-(gRNA-Sc)-(TaU3t)-
(U6-26p)-(Target-3)-(gRNA-Sc)-(OsU3t)

AGTAATTCATCCAGGTCTCCAAGTTCTAGGATTTTCAGAACTGCAACTTATTTTATCAAGGAATCTTTAAACATACGAACAGATCACTTAAAGTTCTTCTGAA
GCAACTTAAAGTTATCAGGCATGCATGGATCTTGGAGGAATCAGATGTGCAGTCAGGGACCATAGCACAAGACAGGCGTCTTCTACTGGTGCTACCAGCA
AATGCTGGAAGCCGGGAACACTGGGTACGTTGGAAACCACGTGATGTGAAGAAGTAAGATAAACTGTAGGAGAAAAGCATTTCGTAGTGGGCCATGAAG
CCTTTCAGGACATGTATTGCAGTATGGGCCGGCCCATTACGCAATTGGACGACAACAAAGACTAGTATTAGTACCACCTCGGCTATCCACATAGATCAAAGC
TGATTTAAAAGAGTTGTGCAGATGATCCGT**GGCG**NNNNNNNNNNNNNNNNNNNGTTTTAGAGCTAGAAATAGCAAGTTAAAATAAGGCTAGTC
CGTTATCAACTTGAAAAAGTGGCACCGAGTCGGTGCTTTTTTTTTTCGTTTTGCATTGAGTTTTCTCCGTCGCATGTTTGCAGTTTTATTTTCCGTTTTGCATT
GAAATTTCTCCGTCTCATGTTTGCAGCGTGTTCAAAAAGTACGCAGCTGTATTTCACTTATTTACGGCGCCACATTTTCATGCCGTTTGTGCCAACTATCCCG
AGCTAGTGAATACAGCTTGGCTTCACACAACACTGGTGACCCGCTGACCTGCTCGTACCTCGTACCGTCGTACGGCACAGCATTTGGAATTAAAGGGTGTG
ATCGATACTGCTTGCTGCTCATGAATCCAAACCACACGGAGTTCAAATTCCCACAGATTAAGGCTCGTCCGTCGCACAAGGTAATGTGTGAATATTATATCTG
TCGTGCAAAATTGCCTGGCCTGCACAATTGCTGTTATAGTTGGCGGCAGGGAGAGTTTTAACA***TTGACTAGCGTGCTGATAATTTGTG****AG*AAATAATAATT
GACAAGTAGATACTGACATTTGAGAAGAGCTTCTGAACTGTTATTAGTAACAAAAATGGAAAGCTGATGCACGGAAAAAGGAAAGAAAAAGCCATACTTT
TTTTTAGGTAGGAAAAGAAAAAGCCATACGAGACTGATGTCTCTCAGATGGGCCGGGATCTGTCTATCTAGCAGGCAGCAGCCCACCAACCTCACGGGCC
AGCAATTACGAGTCCTTCTAAAAGCTCCCGCCGAGGGGCGCTGGCGCTGCTGTGCAGCAGCACGTCTAACATTAGTCCCACCTCGCCAGTTTACAGGGAG
CAGAACCAGCTTATAAGCGGAGGCGCGGCACCAAG**AAGC**GNNNNNNNNNNNNNNNNNNNGTTTTAGAGCTAGAAATAGCAAGTTAAAATAA
GGCTAGTCCGTTATCAACTTGAAAAAGTGGCACCGAGTCGGTGCTTTTTTTTTTGTCCTTCTGTTTTTTTAGTCAGTCTCTTTTTTCAGAAGTACAACATCTT
TTTTTTGTCCTTCTGTTTTTTTAGTCAGTCTCTTTTTTCAGAAGTACAACATCTTTTTTTTGTCCTTCTGTTTTTTTAGTCAGTCTCTTTTTTCAGAAGTACAAC
ATCTTTTTTTTGTCCTTCTGTTTTTTTAGTCAGTCTCTTTTTTCAGAAGTACTCTATGATGTATCGTTCTGGGAAATGTCTGTCTGTCTACAACCCCATAATCTAT
ATTTGCAATCACACATCTAATATTCTCTGTGACAAGACAGCCGAACACGACTTGCCTTCCGCACAATACATCATTTCTTCTTAGCTTTTTTTCTTCTTCTTCGTT
CATACAGTTTTTTTTTGTTTATCAGCTTACATTTTCTTGAACCGTAGCTTTCGTTTTCTTCTTTTTAACTTTCCATTCGGAGTTTTTGTATCTTGTTTCATAGTT**T**
GTCCCAGGATTAGAATGATTAGGCATCGAACCTTCAAGAATTTGATTGAATAAAACATCTTCATTCTTAAGATATGAAGATAATCTTCAAAAGGCCCCTGGG
AATCTGAAAGAAGAGAAGCAGGCCCATTTATATGGGAAAGAACAATAGTATTTCTTATATAGGCCCATTTAAGTTGAAAACAATCTTCAAAAGTCCCACATC
GCTTAGATAAGAAAACGAAGCTGAGTTTATATACAGCTAGAGTCGAAGTAGTGATTGNNNNNNNNNNNNNNNNNNN**GTTT**TAGAGCTAGAAATA
GCAAGTTAAAATAAGGCTAGTCCGTTATCAACTTGAAAAAGTGGCACCGAGTCGGTGCTTTTTTTTTTCGTTTTGCATTGAGTTTTCTCCGTCGCATGTTTG
CAGTTTTATTTTCCGTTTTGCATTGAAATTTCTCCGTCTCATGTTTGCAGCGTGTTCAAAAAGTACGCAGCTGTATTTCACTTATTTACGGCGCCACATTTTCA
TGCCGTTTGTGCCAACTATCCCGAGCTAGTGAATACAGCTTGGCTTCACACAACACTGGTGACCCGCTGACCTGCTCGTACCTCGTACCGTCGTACGGCAC
AGCATTTGGAATTAAAGGGTGTGATCGATACTGCTTGCTGCT

Notes:

1. Underlined letters come from binary vectors, while the others come from PCR fragments.
2. Red letters indicate primer sites.
3. Enlarged and boxed letters indicate 5' protruding ends produced by *Bsa*I digestion.
4. Primer sequences are as follows:

Colony PCR primers (5'→3'):

TaU3p-F2: TTGACTAGCGTGCTGATAATTTGTG

U6-26p-R2: *AGGTTCGATGCCTAATCATTCTAATCCT*

(TaU3p-F2 + U6-26p-R2 = 987 bp)

Sequencing primers (5'→3'):

TaU3p-F2: T*TGACTAGCGTGCTGATAATTTGTG*

TaU3p-R: *CTCACAAATTATCAGCACGCTAGTC*

U6-26p-F: TGTCC*CAGGATTAGAATGATTAGGC*

Sequence of four gRNA expression cassettes for monocots

MT1-PCR + MT2-PCR + MT3T4-PCR2 + pBUN411 et al.

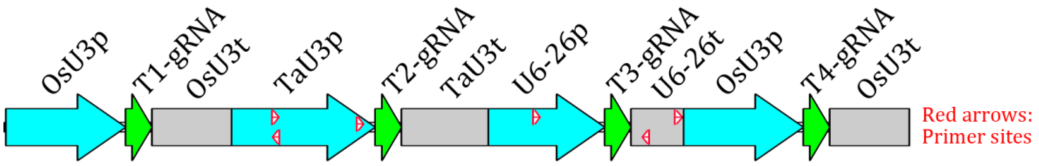

(OsU3p)-(Target-1)-(gRNA-Sc)-(OsU3t)-(TaU3p)-(Target-2)-(gRNA-Sc)-(TaU3t)-
(U6-26p)-(Target-3)-(gRNA-Sc)-(U6-26t)-(OsU3p)-(Target-4)-(gRNA-Sc)-(OsU3t)

AGTAATTCATCCAGGTCTCCAAGTTCTAGGATTTTCAGAACTGCAACTTATTTTATCAAGGAATCTTTAAACATACGAACAGATCACTTAAAGTTCTTCTGAA
GCAACTTAAAGTTATCAGGCATGCATGGATCTTGGAGGAATCAGATGTGCAGTCAGGGACCATAGCACAAGACAGGCGTCTTCTACTGGTGCTACCAGCA
AATGCTGGAAGCCGGGAACACTGGGTACGTTGGAAACCACGTGATGTGAAGAAGTAAGATAAACTGTAGGAGAAAAGCATTTCGTAGTGGGCCATGAAG
CCTTTCAGGACATGTATTGCAGTATGGGCCGGCCCATTACGCAATTGGACGACAACAAAGACTAGTATTAGTACCACCTCGGCTATCCACATAGATCAAAGC
TGATTTAAAAGAGTTGTGCAGATGATCCGT**GGCG**NNNNNNNNNNNNNNNNNNNGTTTTAGAGCTAGAAATAGCAAGTTAAAATAAGGCTAGTC
CGTTATCAACTTGAAAAAGTGGCACCGAGTCGGTGCTTTTTTTTTTCGTTTTGCATTGAGTTTTCTCCGTCGCATGTTTGCAGTTTTATTTTCCGTTTTGCATT
GAAATTTCTCCGTCTCATGTTTGCAGCGTGTTCAAAAAGTACGCAGCTGTATTTCACTTATTTACGGCGCCACATTTTCATGCCGTTTGTGCCAACTATCCCG
AGCTAGTGAATACAGCTTGGCTTCACACAACACTGGTGACCCGCTGACCTGCTCGTACCTCGTACCGTCGTACGGCACAGCATTTGGAATTAAAGGGTGTG
ATCGATACTGCTTGCTGCTCATGAATCCAAACCACACGGAGTTCAAATTCCCACAGATTAAGGCTCGTCCGTCGCACAAGGTAATGTGTGAATATTATATCTG
TCGTGCAAAATTGCCTGGCCTGCACAATTGCTGTTATAGTTGGCGGCAGGGAGAGTTTTAACA*TTGACTAGCGTGCTGATAATTTGTGAG*AAATAATAATT
GACAAGTAGATACTGACATTTGAGAAGAGCTTCTGAACTGTTATTAGTAACAAAAATGGAAAGCTGATGCACGGAAAAAGGAAAGAAAAAGCCATACTTT
TTTTTAGGTAGGAAAAGAAAAAGCCATACGAGACTGATGTCTCTCAGATGGGCCGGGATCTGTCTATCTAGCAGGCAGCAGCCCACCAACCTCACGGGCC
AGCAATTACGAGTCCTTCTAAAAGCTCCCGCCGAGGGGCGCTGGCGCTGCTGTGCAGCAGCACGTCTAACA**TTAGTCCCACCTCGCCAGTTTACAG**GGA
GCAGAACCAGCTTATAAGCGGAGGCGCGGCACCAAG**AAGC**GNNNNNNNNNNNNNNNNNNNGTTTTAGAGCTAGAAATAGCAAGTTAAAATA
AGGCTAGTCCGTTATCAACTTGAAAAAGTGGCACCGAGTCGGTGCTTTTTTTTTTGTCCTTCTGTTTTTTTAGTCAGTCTCTTTTTTCAGAAGTACAACATCT
TTTTTTTGTCCTTCTGTTTTTTTAGTCAGTCTCTTTTTTCAGAAGTACAACATCTTTTTTTTGTCCTTCTGTTTTTTTAGTCAGTCTCTTTTTTCAGAAGTACAA
CATCTTTTTTTTGTCCTTCTGTTTTTTTAGTCAGTCTCTTTTTTCAGAAGTACTCTATGATGTATCGTTCTGGGAAATGTCTGTCTGTCTACAACCCCATAATCTA
TATTTGCAATCACACATCTAATATTCTCTGTGACAAGACAGCCGAACACGACTTGCCTTCCGCACAATACATCATTTCTTCTTAGCTTTTTTTCTTCTTCTTCGT
TCATACAGTTTTTTTTTGTTTATCAGCTTACATTTTCTTGAACCGTAGCTTTCGTTTTCTTCTTTTTAACTTTCCATTCGGAGTTTTTGTATCTTGTTTCATAGTT
TGTCCCAGGATTAGAATGATTAGGCATCGAACCTTCAAGAATTTGATTGAATAAAACATCTTCATTCTTAAGATATGAAGATAATCTTCAAAAGGCCCCTGG
GAATCTGAAAGAAGAGAAGCAGGCCCATTTATATGGGAAAGAACAATAGTATTTCTTATATAGGCCCATTTAAGTTGAAAACAATCTTCAAAAGTCCCACAT
CGCTTAGATAAGAAAACGAAGCTGAGTTTATATACAGCTAGAGTCGAAGTAGTG**ATTG**NNNNNNNNNNNNNNNNNNNGTTTTAGAGCTAGAAAT
AGCAAGTTAAAATAAGGCTAGTCCGTTATCAACTTGAAAAAGTGGCACCGAGTCGGTGCTTTTTTTTGCAAAATTTTCCAGATCGATTTCTTCTTCCTCT**GTT**
CTTCGGCGTTCAATTTCTGGGGTTTTCTCTTCGTTTTCTGTAACTGAAACCTAAAATTTGACCTAAAAAAAATCTCAAATAATATGATTCAGTGGTTTTGTACT
TTTCAGTTAGTT**GAGTTTTGCAGTTCCGATGAGATAAACC**AATAAGTAATTCATCCAGGTCACCAAGTTCTAGGATTTTCAGAACTGCAACTTATTTTATCAA
GGAATCTTTAAACATACGAACAGATCACTTAAAGTTCTTCTGAAGCAACTTAAAGTTATCAGGCATGCATGGATCTTGGAGGAATCAGATGTGCAGTCAGG
GACCATAGCACAAGACAGGCGTCTTCTACTGGTGCTACCAGCAAATGCTGGAAGCCGGGAACACTGGGTACGTTGGAAACCACGTGATGTGAAGAAGTA
AGATAAACTGTAGGAGAAAAGCATTTCGTAGTGGGCCATGAAGCCTTTCAGGACATGTATTGCAGTATGGGCCGGCCCATTACGCAATTGGACGACAACA
AAGACTAGTATTAGTACCACCTCGGCTATCCACATAGATCAAAGCTGATTTAAAAGAGTTGTGCAGATGATCCGTGGCGNNNNNNNNNNNNNNNNNNN
GTTTTAGAGCTAGAAATAGCAAGTTAAAATAAGGCTAGTCCGTTATCAACTTGAAAAAGTGGCACCGAGTCGGTGCTTTTTTTTTTCGTTTTGCATTG
AGTTTTCTCCGTCGCATGTTTGCAGTTTTATTTTCCGTTTTGCATTGAAATTTCTCCGTCTCATGTTTGCAGCGTGTTCAAAAAGTACGCAGCTGTATTTCACT
TATTTACGGCGCCACATTTTCATGCCGTTTGTGCCAACTATCCCGAGCTAGTGAATACAGCTTGGCTTCACACAACACTGGTGACCCGCTGACCTGCTCGTA
CCTCGTACCGTCGTACGGCACAGCATTTGGAATTAAAGGGTGTGATCGATACTGCTTGCTGCT

Notes:

1. Underlined letters come from binary vectors, while the others come from PCR fragments.
2. Red letters indicate primer sites.
3. Enlarged and boxed letters indicate 5' protruding ends produced by *Bsa*I digestion.
4. Primer sequences are as follows:

Colony PCR primers (5'→3'):

TaU3p-F: TTAGTCCCACCTCGCCAGTTTACAG

U6-26t-R: CCCCAGAAATTGAACGCCGAAGAAC

(TaU3p-F + U6-26t-R = 1.2 kb)

Sequencing primers (5'→3'):

TaU3p-F2: TT*GACTAGCGTGCTGATAATTTGTG*

TaU3p-R: *CTCACAAATTATCAGCACGCTAGTC*

U6-26p-F: TGTCCCAGGATTAGAATGATTAGGC

U6-26t-F2: GAGTTTTGCAGTTCCGATGAGATAAACC
