# Supplementary material for: A CRISPR/Cas9 toolkit for multiplex genome editing in plants
Source: BMC Plant Biol. 2014 Nov 29;14:327. doi: 10.1186/s12870-014-0327-y (PMC4262988; doi:10.1186/s12870-014-0327-y)
Supplement: Additional file 5: Methods S4. — Gibson Assembly method for the assembly of three or four gRNAs. [file 12870_2014_327_MOESM5_ESM.pdf]

Additional file 5

Methods S4

Gibson Assembly method for the assembly of three or four gRNAs

Table of contents

Simplified protocol 2
Table S4-1 Nomenclature of PCR products and primers for Gibson Assembly 3
Sequence of DT1T2-PCR0 with Targets 1 and 2 for dicots 4
Sequence of DT2T3-PCR/PCR0 with Targets 2 and 3 for dicots 5
Sequence of DT3T4-PCR with Targets 3 and 4 for dicots 6
Sequence of DT1-T3-PCR with Targets 1, 2 and 3 for dicots 7
Sequence of DT1-T4-PCR with Targets 1, 2, 3 and 4 for dicots 8
Sequence of three gRNA expression cassettes for dicots 9
Sequence of four gRNA expression cassettes for dicots 10
Sequence of MT1T2-PCR0 with Targets 1 and 2 for monocots 11
Sequence of MT2T3-PCR/PCR0 with Targets 2 and 3 for monocots 12
Sequence of MT3T4-PCR with Targets 3 and 4 for monocots 13
Sequence of MT1-T3-PCR with Targets 1, 2 and 3 for monocots 14
Sequence of MT1-T4-PCR with Targets 1, 2, 3 and 4 for monocots 15
Sequence of three gRNA expression cassettes for monocots 16
Sequence of four gRNA expression cassettes for monocots 17

Simplified protocol

1. Manually search for 23-bp target sites (5'-N_{20}NGG-3') within exons of genomic DNA sequences of genes of interest, and then evaluate target specificities on the website of potential off-target finder (http://www.rgenome.net/cas-offinder/). Users can also search for target sites on the website of genome-wide prediction of plant CRISPR/Cas9 target sites (http://www.genome.arizona.edu/crispr/CRISPRsearch.html).
2. Design primers:
   a) Find names of PCR fragments and primers according to plant species (monocots or dicots) and gRNA numbers in Table S4-1.
   b) Find the sequences of the primers according to the names.
   c) Replace 19-nt N in the forward primers with your 19-nt target sequences in front of PAM (NGG), and 19-nt N in the reverse primers with reverse complement sequences of your 19-nt target sequences in front of PAM (NGG).
3. Carry out PCR reactions according to information provided under the sequences of the PCR fragments. Refer to Additional file 3: Methods S2.
4. Set up Gibson Assembly reactions according to the manufacturer's protocol (NEB). As an example, the reaction mixture and reaction conditions are as follows for construction of a vector carrying four gRNAs:

**Component**	**Volume**	**Assembly conditions**
Purified DT1T2-PCR (~100 ng/μl)	2 μl	50 °C, 1 h
Purified DT2T3-PCR (~100 ng/μl)	2 μl	
Purified DT3T4-PCR (~100 ng/μl)	2 μl	
Gibson Assembly Master Mix (2×)	10 μl	
ddH_2O	4 μl	
Total volume	20 μl	

5. The fragment of corrected size (1.9-kb) was gel purified and used as PCR template for the second round of PCR amplification.
6. The second round of PCR products (DT1-T4-PCR) were purified and mixed with any of the binary vectors described in this report to set up Golden Gate reaction. Refer to Additional file 3: Methods S2.
7. Transform *E.coli* competent cells, select positive clones on kanamycin LB agar plates.
8. Identify correct clones by colony PCR and verify them by sequencing.

Table S4-1 Nomenclature of PCR products and primers for Gibson Assembly

For dicots					
The 1st Round of PCR			Gibson Assembly	The 2nd Round of PCR	Number of gRNAs
	DT1T2-PCR0<br>DT1-BsF/DT1-F0<br>DT2-R	DT2T3-PCR0<br>DT2-F/DT2-F0<br>DT3-R			
DT2T3-PCR<br>DT2-F/DT2-F0<br>DT3-R0/DT3-BsR	+	-	DT1-T3-GA	DT1-T3-PCR<br>DT1-BsF<br>DT3-BsR	3
DT3T4-PCR<br>DT3-F/DT3-F0<br>DT4-R0/DT4-BsR	+	+	DT1-T4-GA	DT1-T4-PCR<br>DT1-BsF<br>DT4-BsR	4
**For monocots**					
The 1st Round of PCR			Gibson Assembly	The 2nd Round of PCR	Number of gRNAs
	MT1T2-PCR0<br>MT1-BsF/MT1-F0<br>MT2-R0	MT2T3-PCR0<br>MT2-F/MT2-F0<br>MT3-R0			
MT2T3-PCR<br>MT2-F/MT2-F0<br>MT3-R0/MT3-BsR	+	-	MT1-T3-GA	MT1-T3-PCR<br>MT1-BsF<br>MT3-BsR	3
MT3T4-PCR<br>MT3-F/MT3-F0<br>MT4-R0/MT4-BsR	+	+	MT1-T4-GA	MT1-T4-PCR<br>MT1-BsF<br>MT4-BsR	4

Notes:

1. The primer names are under the PCR names and highlighted.
2. "+" or "-" indicates whether or not the PCR fragment in the column is mixed with the PCR fragment in the row of Column 1 for Gibson Assembly.
3. When using more than two PCR primers, dilute the F0/R0 primers to 20 times of the F/R primers.

Sequence of DT1T2-PCR0 with Targets 1 and 2 for dicots

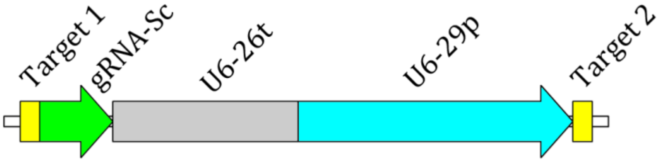

(Target-1)-(gRNA-Sc)-(U6-26t)-(U6-29p)-(Target-2)

ATATATGGTCTCGATTGNNNNNNNNNNNNNNNNNNNGTTTTAGAGCTAGAAATAGCAAGTTAAAATAAG
GCTAGTCCGTTATCAACTTGAAAAAGTGGCACCGAGTCGGTGCTTTTTTTTGCAAAATTTTCCAGATCGATTT
CTTCTTCCTCTGTTCTTCGGCGTTCAATTTCTGGGGTTTTCTCTTCGTTTTCTGTAACTGAAACCTAAAATTTG
ACCTAAAAAAAATCTCAAATAATATGATTCAGTGGTTTTGTACTTTTCAGTTAGTTGAGTTTTGCAGTTCCGAT
GAGATAAACCAATATTAATCCAAACTACTGCAGCCTGACAGACAAATGAGGATGCAAACAATTTTAAAGTTT
ATCTAACGCTAGCTGTTTTGTTTCTTCTCTCTGGTGCACCAACGACGGCGTTTTCTCAATCATAAAGAGGCT
TGTTTTACTTAAGGCCAATAATGTTGATGGATCGAAAGAAGAGGGCTTTTAATAAACGAGCCCGTTTAAG
CTGTAAACGATGTCAAAAACATCCCACATCGTTCAGTTGAAAATAGAAGCTCTGTTTATATATTGGTAGAG
TCGACTAAGAGATTGNNNNNNNNNNNNNNNNNNNGTT

Primers:

DT1-BsF: ATATATGGTCTCGATTGNNNNNNNNNNNNNNNNNNNGTT

DT1-F0: TGNNNNNNNNNNNNNNNNNNNGTTTTAGAGCTAGAAATAGC

DT2-R: AACNNNNNNNNNNNNNNNNNNNCAATCTCTTAGTCGACTCTAC

Template: pCBC-DT1T2

Length: 612-bp

Notes:

1. The 19-nt N in primers represent any 19-nt target sequence (forward primers) or reverse complement sequence of any 19-nt target sequence (reverse primers) in front of PAM (NGG).
2. Use DT1-BsF/DT1-F0/DT2-R three-primer mixture with DT1-F0 diluted to 20 times of DT1-BsF or DT2-R, resulting in DT1T2-PCR0.

Sequence of DT2T3-PCR/PCR0 with Targets 2 and 3 for dicots

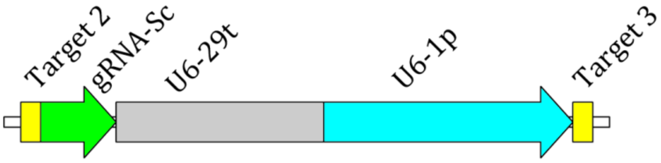

(Target-2)-(gRNA-Sc)-(U6-29t)-(U6-1p)-(Target-3)

GTAGAGTCGACTAAGAGATTGNNNNNNNNNNNNNNNNNNNGTTTTAGAGCTAGAAATAGCAAGTTAAA
ATAAGGCTAGTCCGTTATCAACTTGAAAAAGTGGCACCGAGTCGGTGCTTTTTTTGGATAGAATTTCCCAGC
TTTTTTGCGTGTTTCAGCTCTCATGATCCTTGGCCAATGGGTGTAGTAAATTTTCTGCACATTCATTGGATG
GAAAATAATGGTTTTAGCTTTAGGGAATAAGAAAAGTGTATAGGAAGGGGATTTTTGTACAATCACATTT
GAATTAGGTCTTTGAAATGACAGGGAATGAGGACATATGATGAGACGGTCATTGTTTTAGTTCCACCACG
ATTATATTTGAAATTTACGTGAGTGTGAGTGAGACTTGCATAAGAAAATAAAATCTTTAGTTGGGAAAAA
ATTCAATAATATAAATGGGCTTGAGAAGGAAGCGAGGGATAGGCCTTTTTCTAAAATAGGCCCATTTAAG
CTATTAACAATCTTCAAAAGTACCACAGCGCTTAGGTAAAGAAAGCAGCTGAGTTTATATATGGTTAGAG
ACGAAGTAGTGATTGNNNNNNNNNNNNNNNNNNNGTT

Primers:

DT2-F: GTAGAGTCGACTAAGAGATTGNNNNNNNNNNNNNNNNNNNGTT

DT2-F0: 5' TGNNNNNNNNNNNNNNNNNNNGTTTTAGAGCTAGAAATAGC 3'

DT3-R0/R: 5' AACNNNNNNNNNNNNNNNNNNNCAATCACTACTTCGTCTCTAACC 3'

DT3-BsR: 5' ATTATTGGTCTCGAAACNNNNNNNNNNNNNNNNNNNC 3'

Template: pCBC-DT2T3

Length: 613-bp (DT2T3-PCR) or 599-bp (DT2T3-PCR0)

Notes:

1. The 19-nt N in primers represent any 19-nt target sequence (forward primers) or reverse complement sequence of any 19-nt target sequence (reverse primers) in front of PAM (NGG).
2. For the assembly of three gRNA expression cassettes, use DT2-F/DT2-F0/DT3-R0/DT3-BsR four-primer mixture with DT2-F0/DT3-R0 diluted to 20 times of DT2-F or DT3-BsR, resulting in DT2T3-PCR.
3. For the assembly of more than three gRNA expression cassettes, use DT2-F/DT2-F0/DT3-R three-primer mixture with DT2-F0 diluted to 20 times of DT2-F or DT3-R, resulting in DT2T3-PCR0.

Sequence of DT3T4-PCR with Targets 3 and 4 for dicots

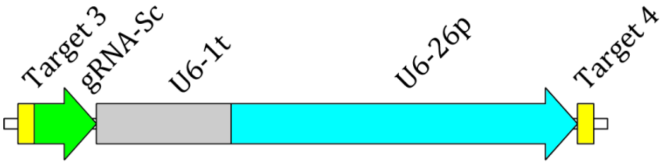

(Target-3)-(gRNA-Sc)-(U61-t)-(U6-26p)-(Target-4)

GGTTAGAGACGAAGTAGTGATTGNNNNNNNNNNNNNNNNNNNGTTTTAGAGCTAGAAATAGCAAGTTA
AAATAAGGCTAGTCCGTTATCAACTTGAAAAAGTGGCACCGAGTCGGTGCTTTTTTTTGGCAAAAATTTTCA
GATTTTTTCTTCATCTGTAGATTTCTGGGTTTTTTTTTCCGTTTCGTGAATCATAAGTGAAGTTTTGGATGCA
AATCTGCGCGAAAAAAGTTGGACCTGCAATGAGCTTATTTAGATAGCTAAGACAAAGTGATTGGTCCGTT
CGACTTGCCTTCCGCACAATACATCATTTCTTCTTAGCTTTTTTTCTTCTTCTTCGTTCATACAGTTTTTTTTTGT
TTATCAGCTTACATTTTCTTGAACCGTAGCTTTCGTTTTCTTCTTTTTAACTTTCCATTCGGAGTTTTTGTATCTT
GTTTCATAGTTTGTCCCAGGATTAGAATGATTAGGCATCGAACCTTCAAGAATTTGATTGAATAAAACATCTTC
ATTCTTAAGATATGAAGATAATCTTCAAAAGGCCCCTGGGAATCTGAAAGAAGAGAAGCAGGCCCATTTATAT
GGGAAAGAACAATAGTATTTCTTATATAGGCCCATTTAAGTTGAAAACAATCTTCAAAAGTCCCACATCGCTT
AGATAAGAAAACGAAGCTGAGTTTATATACAGCTAGAGTCGAAGTAGTGATTGNNNNNNNNNNNNNNNN
NNNGTTTCGAGACCAATAAT

Primers:

DT3-F: GGTTAGAGACGAAGTAGTGATTGNNNNNNNNNNNNNNNNNNNGTT

DT3-F0: TGNNNNNNNNNNNNNNNNNNNGTTTTAGAGCTAGAAATAGC

DT4-R0: AACNNNNNNNNNNNNNNNNNNNCAATCACTACTTCGACTCTAGC

DT4-BsR: ATTATTGGTCTCGAAACNNNNNNNNNNNNNNNNNNNC

Template: pCBC-DT3T4

Length: 746-bp

Notes:

1. The 19-nt N in primers represent any 19-nt target sequence (forward primers) or reverse complement sequence of any 19-nt target sequence (reverse primers) in front of PAM (NGG).
2. For the assembly of four gRNA expression cassettes, use DT3-F/DT3-F0/DT4-R0/DT4-BsR four-primer mixture with DT3-F0/DT4-R0 diluted to 20 times of DT3-F or DT4-BsR.

Sequence of DT1-T3-PCR with Targets 1, 2 and 3 for dicots

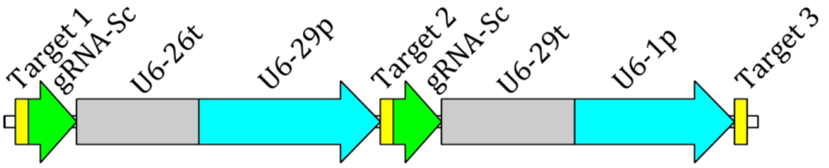

(Target-1)-(gRNA-Sc)-(U6-26t)-(U6-29p)-(Target-2)-(gRNA-Sc)-(U6-29t)-(U6-1p)-(Target-3)

ATATATGGTCTCGATTGNNNNNNNNNNNNNNNNNNNGTTTTAGAGCTAGAAATAGCAAGTTAAAATAAG
GCTAGTCCGTTATCAACTTGAAAAAGTGGCACCGAGTCGGTGCTTTTTTTTGCAAAATTTTCCAGATCGATTT
CTTCTTCCTCTGTTCTTCGGCGTTCAATTTCTGGGGTTTTCTCTTCGTTTTCTGTAACTGAAACCTAAAATTTG
ACCTAAAAAAAATCTCAAATAATATGATTCAGTGGTTTTGTACTTTTCAGTTAGTTGAGTTTTGCAGTTCCGAT
GAGATAAACCAATATTAATCCAAACTACTGCAGCCTGACAGACAAATGAGGATGCAAACAATTTTAAAGTTT
ATCTAACGCTAGCTGTTTTGTTTCTTCTCTCTGGTGCACCAACGACGGCGTTTTCTCAATCATAAAGAGGCT
TGTTTTACTTAAGGCCAATAATGTTGATGGATCGAAAGAAGAGGGCTTTTAATAAACGAGCCCGTTTAAG
CTGTAAACGATGTCAAAAACATCCCACATCGTTCAGTTGAAAATAGAAGCTCTGTTTATATATTGGTAGAG
TCGACTAAGAGATTGNNNNNNNNNNNNNNNNNNNGTTTTAGAGCTAGAAATAGCAAGTTAAAATAAGG
CTAGTCCGTTATCAACTTGAAAAAGTGGCACCGAGTCGGTGCTTTTTTTGGATAGAATTTCCCAGCTTTTTTG
CGTGTTTCAGCTCTCATGATCCTTGGCCAATGGGTGTAGTAAATTTTCTGCACATTCATTGGATGGAAAAT
AATGGTTTTAGCTTTAGGGAATAAGAAAAGTGTATAGGAAGGGGATTTTTGTACAATCACATTTGAATTA
GGTCTTTGAAATGACAGGGAATGAGGACATATGATGAGACGGTCATTGTTTTAGTTCCACCACGATTATA
TTTGAAATTTACGTGAGTGTGAGTGAGACTTGCATAAGAAAATAAAATCTTTAGTTGGGAAAAAATTCAAT
AATATAAATGGGCTTGAGAAGGAAGCGAGGGATAGGCCTTTTTCTAAAATAGGCCCATTTAAGCTATTAA
CAATCTTCAAAAGTACCACAGCGCTTAGGTAAAGAAAGCAGCTGAGTTTATATATGGTTAGAGACGAAGT
AGTGATTGNNNNNNNNNNNNNNNNNNNGTTTCGAGACCAATAAT

Primers:

DT1-BsF: ATATATGGTCTCGATTGNNNNNNNNNNNNNNNNNNNGTT

DT3-BsR: ATTATTGGTCTCGAAACNNNNNNNNNNNNNNNNNNNCAA

Template:

Gibson Assembly product of the DT1T2-PCR and DT2T3-PCR fragments was used as template for PCR amplification with DT1-BsF/DT3-BsR primers, resulting in DT1-T3-PCR.

Length: 1.2-kb

Sequence of DT1-T4-PCR with Targets 1, 2, 3 and 4 for dicots

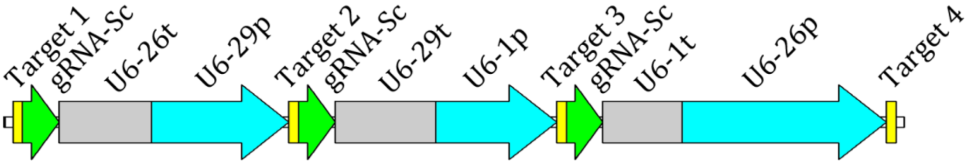

(Target-1)-(gRNA-Sc)-(U6-26t)-(U6-29p)-(Target-2)-(gRNA-Sc)-(U6-29t)- (U6-1p)-(Target-3)-(gRNA-Sc)-(U6-1t)-(U6-26p)-(Target-4)

ATATATGGTCTCGATTGNNNNNNNNNNNNNNNNNNNGTTTTAGAGCTAGAAATAGCAAGTTAAAATAAG
GCTAGTCCGTTATCAACTTGAAAAAGTGGCACCGAGTCGGTGCTTTTTTTTGCAAAATTTTCCAGATCGATTT
CTTCTTCCTCTGTTCTTCGGCGTTCAATTTCTGGGGTTTTCTCTTCGTTTTCTGTAACTGAAACCTAAAATTTG
ACCTAAAAAAAATCTCAAATAATATGATTCAGTGGTTTTGTACTTTTCAGTTAGTTGAGTTTTGCAGTTCCGAT
GAGATAAACCAATATTAATCCAAACTACTGCAGCCTGACAGACAAATGAGGATGCAAACAATTTTAAAGTTT
ATCTAACGCTAGCTGTTTTGTTTCTTCTCTCTGGTGCACCAACGACGGCGTTTTCTCAATCATAAAGAGGCT
TGTTTTACTTAAGGCCAATAATGTTGATGGATCGAAAGAAGAGGGCTTTTAATAAACGAGCCCGTTTAAG
CTGTAAACGATGTCAAAAACATCCCACATCGTTCAGTTGAAAATAGAAGCTCTGTTTATATATTGGTAGAG
TCGACTAAGAGATTGNNNNNNNNNNNNNNNNNNNGTTTTAGAGCTAGAAATAGCAAGTTAAAATAAGG
CTAGTCCGTTATCAACTTGAAAAAGTGGCACCGAGTCGGTGCTTTTTTTGGATAGAATTTCCCAGCTTTTTTG
CGTGTTTCAGCTCTCATGATCCTTGGCCAATGGGTGTAGTAAATTTTCTGCACATTCATTGGATGGAAAAT
AATGGTTTTAGCTTTAGGGAATAAGAAAAGTGTATAGGAAGGGGATTTTTGTACAATCACATTTGAATTA
GGTCTTTGAAATGACAGGGAATGAGGACATATGATGAGACGGTCATTGTTTTAGTTCCACCACGATTATA
TTTGAAATTTACGTGAGTGTGAGTGAGACTTGCATAAGAAAATAAAATCTTTAGTTGGGAAAAAATTCAAT
AATATAAATGGGCTTGAGAAGGAAGCGAGGGATAGGCCTTTTTCTAAAATAGGCCCATTTAAGCTATTAA
CAATCTTCAAAAGTACCACAGCGCTTAGGTAAAGAAAGCAGCTGAGTTTATATATGGTTAGAGACGAAGT
AGTGATTGNNNNNNNNNNNNNNNNNNNGTTTTAGAGCTAGAAATAGCAAGTTAAAATAAGGCTAGTCC
GTTATCAACTTGAAAAAGTGGCACCGAGTCGGTGCTTTTTTTTGGCAAAAATTTTCAGATTTTTTCTTCATCT
GTAGATTTCTGGGTTTTTTTTTCCGTTTCGTGAATCATAAGTGAAGTTTTGGATGCAAATCTGCGCGAAAA
AAGTTGGACCTGCAATGAGCTTATTTAGATAGCTAAGACAAAGTGATTGGTCCGTTCGACTTGCCTTCCGC
ACAATACATCATTTCTTCTTAGCTTTTTTTCTTCTTCTTCGTTCATACAGTTTTTTTTTGTTTATCAGCTTACATTT
TCTTGAACCGTAGCTTTCGTTTTCTTCTTTTTAACTTTCCATTCGGAGTTTTTGTATCTTGTTTCATAGTTTGTC
CCAGGATTAGAATGATTAGGCATCGAACCTTCAAGAATTTGATTGAATAAAACATCTTCATTCTTAAGATATGA
AGATAATCTTCAAAAGGCCCCTGGGAATCTGAAAGAAGAGAAGCAGGCCCATTTATATGGGAAAGAACAAT
AGTATTTCTTATATAGGCCCATTTAAGTTGAAAACAATCTTCAAAAGTCCCACATCGCTTAGATAAGAAAACGA
AGCTGAGTTTATATACAGCTAGAGTCGAAGTAGTGATTGNNNNNNNNNNNNNNNNNNNGTTTCGAGACC
AATAAT

Primers:

DT1-BsF: ATATATGGTCTCGATTGNNNNNNNNNNNNNNNNNNNGTT

DT4-BsR: ATTATTGGTCTCGAAACNNNNNNNNNNNNNNNNNNNCAA

Template:

Gibson Assembly product of the DT1T2-PCR, DT2T3-PCR and DT3T4-PCR fragments was used as template for the second round of PCR amplification with DT1-BsF/DT4-BsR primers, resulting in DT1-T4-PCR.

Length: 1.9-kb

Sequence of three gRNA expression cassettes for dicots

DT1-T3-PCR + pHSN401 et al.

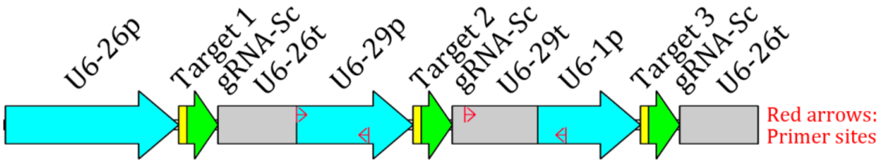

(U6-26p)-(Target-1)-(gRNA-Sc)-(U6-26t)-(U6-29p)-(Target-2)-(gRNA-Sc)-(U6-29t)-
(U6-1p)-(Target-3)-(gRNA-Sc)-(U6-26t)

CGACTTGCCTTCCGCACAATACATCATTTCTTCTTAGCTTTTTTTCTTCTTCTTCGTTCATACAGTTTTTTTTTGT
TTATCAGCTTACATTTTCTTGAACCGTAGCTTTCGTTTTCTTCTTTTTAACTTTCCATTCGGAGTTTTTGTATCTT
GTTTCATAGTTTGTCCCAGGATTAGAATGATTAGGCATCGAACCTTCAAGAATTTGATTGAATAAAACATCTTC
ATTCTTAAGATATGAAGATAATCTTCAAAAGGCCCCTGGGAATCTGAAAGAAGAGAAGCAGGCCCATTTATAT
GGGAAAGAACAATAGTATTTCTTATATAGGCCCATTTAAGTTGAAAACAATCTTCAAAAGTCCCACATCGCTT
AGATAAGAAAACGAAGCTGAGTTTATATACAGCTAGAGTCGAAGTAGTGATTGNNNNNNNNNNNNNNNN
NNNGTTTTAGAGCTAGAAATAGCAAGTTAAAATAAGGCTAGTCCGTTATCAACTTGAAAAAGTGGCACCGA
GTCGGTGCTTTTTTTTGCAAAATTTTCCAGATCGATTTCTTCTTCCTCTGTTCTTCGGCGTTCAATTTCTGGGG
TTTTCTCTTCGTTTTCTGTAACTGAAACCTAAAATTTGACCTAAAAAAAATCTCAAATAATATGATTCAGTGGTT
TTGTACTTTTCAGTTAGTTGAGTTTTGCAGTTCCGATGAGATAAACCAATA**TTAATCCAAACTACTGCAGCCT**
GACAGACAAATGAGGATGCAAACAATTTTAAAGTTTATCTAACGCTAGCTGTTTTGTTTCTTCTCTCTGGTG
CACCAACGACGGCGTTTTCTCAATCATAAAGAGGCTTGTTTTACTTAAGGCCAATAAT**GTTGATGGATCGA**
AAGAAGAGGGCTTTTAATAAACGAGCCCGTTTAAGCTGTAAACGATGTCAAAAACATCCCACATCGTTCA
GTTGAAAATAGAAGCTCTGTTTATATATTGGTAGAGTCGACTAAGAGATTGNNNNNNNNNNNNNNNNN
NNGTTTTAGAGCTAGAAATAGCAAGTTAAAATAAGGCTAGTCCGTTATCAACTTGAAAAAGTGGCACCGAGT
CGGTGCTTTTTTTGGATAGAATTTCCCAGCTTTTTTG**CGTGTTTCAGCTCTCATGATCCTTG**GCCAATGGGT
GTAGTAAATTTTCTGCACATTCATTGGATGGAAAATAATGGTTTTAGCTTTAGGGAATAAGAAAAGTGTAT
AGGAAGGGGATTTTTGTACAATCACATTTGAATTAGGTCTTTGAAATGACAGGGAATGAGGACATATGAT
GAGACGGTCATTGTTTTAGTTCCACCACGATTATATTTGAAATTTA**CGTGAGTGTGAGTGAGACTTGCATA**
AGAAAATAAAATCTTTAGTTGGGAAAAAATTCAATAATATAAATGGGCTTGAGAAGGAAGCGAGGGATA
GGCCTTTTTCTAAAATAGGCCCATTTAAGCTATTAACAATCTTCAAAAGTACCACAGCGCTTAGGTAAAGA
AAGCAGCTGAGTTTATATATGGTTAGAGACGAAGTAGTGATTGNNNNNNNNNNNNNNNNNNNGTTTT
AGAGCTAGAAATAGCAAGTTAAAATAAGGCTAGTCCGTTATCAACTTGAAAAAGTGGCACCGAGTCGGTGC
TTTTTTTTGCAAAATTTTCCAGATCGATTTCTTCTTCCTCTGTTCTTCGGCGTTCAATTTCTGGGGTTTTCTCTT
CGTTTTCTGTAACTGAAACCTAAAATTTGACCTAAAAAAAATCTCAAATAATATGATTCAGTGGTTTTGTACTT
TTCAGTTAGTTGAGTTTTGCAGTTCCGATGAGATAAACCAATA

Notes:

1. Underlined letters come from binary vectors, while the others come from PCR fragments.
2. Red letters indicate primer sites.
3. Primer sequences are as follows:

Colony PCR primers (5'→3')
U6-29p-F: TTAATCCAAACTACTGCAGCCTGAC
U6-1p-R: TATGCAAGTCTCACTCACACTCACG
(U6-29p-F + U6-1p-R = 659 bp)

Sequencing primers (5'→3'):
U6-29p-F: TTAATCCAAACTACTGCAGCCTGAC
U6-29p-R: AGCCCTCTTCTTTCGATCCATCAAC
U6-29t-F: CGTGTTTCAGCTCTCATGATCCTTG

Sequence of four gRNA expression cassettes for dicots

DT1-T4-PCR + pHSN401 et al.

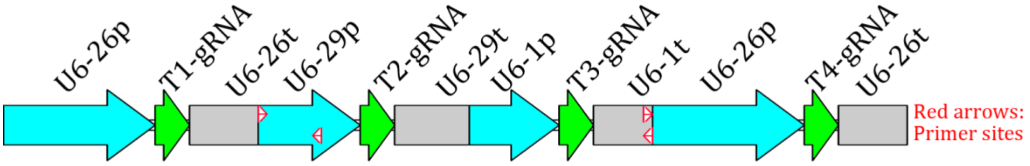

(U6-26p)-(Target-1)-(gRNA-Sc)-(U6-26t)-(U6-29p)-(Target-2)-(gRNA-Sc)-(U6-29t)-(U6-1p)-(Target-3)- (gRNA-Sc)-(U6-1t)-(U6-26p)-(Target-4)-(gRNA-Sc)-(U6-26t)

CGACTTGCCTTCCGCACAATACATCATTTCTTCTTAGCTTTTTTTCTTCTTCTTCGTTCATACAGTTTTTTTTTGTTTATCAGCTTAC
ATTTTCTTGAACCGTAGCTTTCGTTTTCTTCTTTTTAACTTTCCATTCGGAGTTTTTGTATCTTGTTTCATAGTTTGTCCCAGGATTA
GAATGATTAGGCATCGAACCTTCAAGAATTTGATTGAATAAAACATCTTCATTCTTAAGATATGAAGATAATCTTCAAAAGGCCCC
TGGGAATCTGAAAGAAGAGAAGCAGGCCCATTTATATGGGAAAGAACAATAGTATTTCTTATATAGGCCCATTTAAGTTGAAAAC
AATCTTCAAAAGTCCCACATCGCTTAGATAAGAAAACGAAGCTGAGTTTATATACAGCTAGAGTCGAAGTAGTGATTGNNNNNN
NNNNNNNNNNNNNGTTTTAGAGCTAGAAATAGCAAGTTAAAATAAGGCTAGTCCGTTATCAACTTGAAAAAGTGGCACCGAG
TCGGTGCTTTTTTTTGCAAAATTTCCAGATCGATTTCTTCTTCCTCTGTTCTTCGGCGTTCAATTTCTGGGGTTTTCTCTTCGTTT
TCTGTAACTGAAACCTAAAATTTGACCTAAAAAAAATCTCAAATAATATGATTCAGTGGTTTTGTACTTTTCAGTTAGTTGAGTTTT
GCAGTTCCGATGAGATAAACCAATA**TTAATCCAAACTACTGCAGCCTGAC**AGACAAATGAGGATGCAAACAATTTTAAAGTTT
ATCTAACGCTAGCTGTTTTGTTTCTTCTCTCTGGTGCACCAACGACGGCGTTTTCTCAATCATAAAGAGGCTTGTTTTACTTAA
GGCCAATAAT**GTTGATGGATCGAAAGAAGAGGGCT**TTTAATAAACGAGCCCGTTTAAGCTGTAAACGATGTCAAAAACATC
CCACATCGTTCAGTTGAAAATAGAAGCTCTGTTTATATATTGGTAGAGTCGACTAAGAGATTGNNNNNNNNNNNNNNNNN
NNGTTTTAGAGCTAGAAATAGCAAGTTAAAATAAGGCTAGTCCGTTATCAACTTGAAAAAGTGGCACCGAGTCGGTGCTTTTTT
TGGATAGAATTTCCCAGCTTTTTTGCGTGTTTCAGCTCTCATGATCCTTGGCCAATGGGTGTAGTAAATTTTCTGCACATTCAT
TGGATGGAAAATAATGGTTTTAGCTTTAGGGAATAAGAAAAGTGTATAGGAAGGGGATTTTTGTACAATCACATTTGAATTA
GGTCTTTGAAATGACAGGGAATGAGGACATATGATGAGACGGTCATTGTTTTAGTTCCACCACGATTATATTTGAAATTTACG
TGAGTGTGAGTGAGACTTGCATAAGAAAATAAAATCTTTAGTTGGGAAAAAAATTCAATAATATAAATGGGCTTGAGAAGGA
AGCGAGGGATAGGCCTTTTTCTAAAATAGGCCCATTTAAGCTATTAACAATCTTCAAAAGTACCACAGCGCTTAGGTAAAGA
AAGCAGCTGAGTTTATATATGGTTAGAGACGAAGTAGTGATTGNNNNNNNNNNNNNNNNNNNGTTTTAGAGCTAGAAAT
AGCAAGTTAAAATAAGGCTAGTCCGTTATCAACTTGAAAAAGTGGCACCGAGTCGGTGCTTTTTTTTGGCAAAATTTTCAGAT
TTTTTCTTCATCTGTAGATTTCTGGGTTTTTTTTTCCGTTTCGTGAATCATAAGTGAAGTTTTGGATGCAAATCTGCGCGAAAA
AAGTTGGACCTGCAATGAGCTTATTTAGATA**GCTAAGACAAAGTGATTGGTCCGTT**CGACTTGCCTTCCGCACAATACATCAT
TTCTTCTTAGCTTTTTTTCTTCTTCTTCGTTCATACAGTTTTTTTTTGTTTATCAGCTTACATTTTCTTGAACCGTAGCTTTCGTTTTC
TTCTTTTTAACTTTCCATTCGGAGTTTTTGTATCTTGTTTCATAGTTTGTCCCAGGATTAGAATGATTAGGCATCGAACCTTCAAGA
ATTTGATTGAATAAAACATCTTCATTCTTAAGATATGAAGATAATCTTCAAAAGGCCCCTGGGAATCTGAAAGAAGAGAAGCAGG
CCCATTTATATGGGAAAGAACAATAGTATTTCTTATATAGGCCCATTTAAGTTGAAAACAATCTTCAAAAGTCCCACATCGCTTAGA
TAAGAAAACGAAGCTGAGTTTATATACAGCTAGAGTCGAAGTAGTGATTGNNNNNNNNNNNNNNNNNNNGTTTTAGAGCTA
GAAATAGCAAGTTAAAATAAGGCTAGTCCGTTATCAACTTGAAAAAGTGGCACCGAGTCGGTGCTTTTTTTTGCAAAATTTTCCA
GATCGATTTCTTCTTCCTCTGTTCTTCGGCGTTCAATTTCTGGGGTTTTCTCTTCGTTTTCTGTAACTGAAACCTAAAATTTGACCT
AAAAAAAATCTCAAATAATATGATTCAGTGGTTTTGTACTTTTCAGTTAGTTGAGTTTTGCAGTTCCGATGAGATAAACCAATA

Notes:

1. Underlined letters come from binary vectors, while the others come from PCR fragments.
2. Red letters indicate primer sites.
3. Primer sequences are as follows:

Colony PCR primers (5'→3'):
U6-29p-F: TTAATCCAAACTACTGCAGCCTGAC
U6-1t-R: AACGGACCAATCACTTTGTCTTAGC
(U6-29p-F + U6-1t-R = 1.1 kb)

Sequencing primers (5'→3'):
U6-29p-F: TTAATCCAAACTACTGCAGCCTGAC
U6-29p-R: AGCCCTCTTCTTTCGATCCATCAAC
U6-1t-F: GCTAAGACAAAGTGATTGGTCCGTT
U6-1t-R: AACGGACCAATCACTTTGTCTTAGC

Sequence of MT1T2-PCR0 with Targets 1 and 2 for monocots

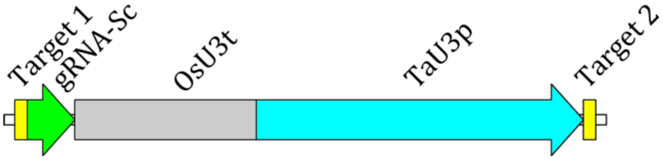

(Target-1)-(gRNA-Sc)-(OsU3t)-(TaU3p)-(Target-2)

ATATATGGTCTCTGGCGNNNNNNNNNNNNNNNNNNNGTTTTAGAGCTAGAAATAGCAAGTTAAAATAAG
GCTAGTCCGTTATCAACTTGAAAAAGTGGCACCGAGTCGGTGCTTTTTTTTTTCGTTTTGCATTGAGTTTTCT
CCGTCGCATGTTTGCAGTTTTATTTTCCGTTTTGCATTGAAATTTCTCCGTCTCATGTTTGCAGCGTGTTCAAA
AAGTACGCAGCTGTATTTCACTTATTTACGGCGCCACATTTTCATGCCGTTTGTGCCAACTATCCCGAGCTAGT
GAATACAGCTTGGCTTCACACAACACTGGTGACCCGCTGACCTGCTCGTACCTCGTACCGTCGTACGGCACA
GCATTTGGAATTAAAGGGTGTGATCGATACTGCTTGCTGCTCATGAATCCAAACCACACGGAGTTCAAATTCC
CACAGATTAAGGCTCGTCCGTCGCACAAGGTAATGTGTGAATATTATATCTGTCGTGCAAAATTGCCTGGCCT
GCACAATTGCTGTTATAGTTGGCGGCAGGGAGAGTTTTAACATTGACTAGCGTGCTGATAATTTGTGAGAAA
TAATAATTGACAAGTAGATACTGACATTTGAGAAGAGCTTCTGAACTGTTATTAGTAACAAAAATGGAAAGCT
GATGCACGGAAAAAGGAAAGAAAAAGCCATACTTTTTTTTAGGTAGGAAAAGAAAAAGCCATACGAGACT
GATGTCTCTCAGATGGGCCGGGATCTGTCTATCTAGCAGGCAGCAGCCCACCAACCTCACGGGCCAGCAATT
ACGAGTCCTTCTAAAAGCTCCCGCCGAGGGGCGCTGGCGCTGCTGTGCAGCAGCACGTCTAACATTAGTCC
CACCTCGCCAGTTTACAGGGAGCAGAACCAGCTTATAAGCGGAGGCGCGGCACCAAGAAGCGNNNNNNN
NNNNNNNNNNNNGTT

Primers:

MT1-BsF: ATATATGGTCTCTGGCGNNNNNNNNNNNNNNNNNNNGTT

MT1-F0: TGNNNNNNNNNNNNNNNNNNNGTTTTAGAGCTAGAAATAGC

MT2-R: AACNNNNNNNNNNNNNNNNNNNCGCTTCTTGGTGCC

Template: pCBC-MT1T2

Length: 950-bp

Notes:

1. The 19-nt N in primers represent any 19-nt target sequence (forward primers) or reverse complement sequence of any 19-nt target sequence (reverse primers) in front of PAM (NGG).
2. For the assembly of more than two gRNA expression cassettes, use MT1-BsF/MT1-F0/MT2-R three-primer mixture with MT1-F0 primers diluted to 20 times of MT1-BsF or MT2-R, resulting in MT1T2-PCR0.

Sequence of MT2T3-PCR/PCR0 with Targets 2 and 3 for monocots

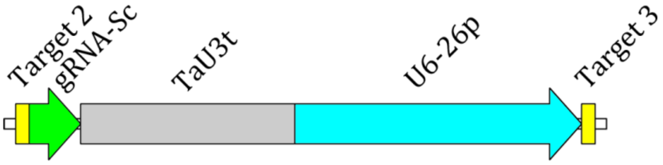

(Target-2)-(gRNA-Sc)-(TaU3t)-(U6-26p)-(Target-3)

GGCACCAAGAAGCGNNNNNNNNNNNNNNNNNNNGTTTTAGAGCTAGAAATAGCAAGTTAAAATAAGGC
TAGTCCGTTATCAACTTGAAAAAGTGGCACCGAGTCGGTGCTTTTTTTTTTGTCCTTCTGTTTTTTTAGTCAGT
CTCTTTTTTCAGAAGTACAACATCTTTTTTTTGTCCTTCTGTTTTTTTAGTCAGTCTCTTTTTTCAGAAGTACAA
CATCTTTTTTTTGTCCTTCTGTTTTTTTAGTCAGTCTCTTTTTTCAGAAGTACAACATCTTTTTTTTGTCCTTCTG
TTTTTTTAGTCAGTCTCTTTTTTCAGAAGTACTCTATGATGTATCGTTCTGGGAAATGTCTGTCTGTCTACAACC
CCATAATCTATATTTGCAATCACACATCTAATATTCTCTGTGACAAGACAGCCGAACACGACTTGCCTTCCGCA
CAATACATCATTTCTTCTTAGCTTTTTTTCTTCTTCTTCGTTCATACAGTTTTTTTTTGTTTATCAGCTTACATTTT
CTTGAACCGTAGCTTTCGTTTTCTTCTTTTTAACTTTCCATTCGGAGTTTTTGTATCTTGTTTCATAGTTTGTCC
CAGGATTAGAATGATTAGGCATCGAACCTTCAAGAATTTGATTGAATAAAACATCTTCATTCTTAAGATATGAA
GATAATCTTCAAAAGGCCCCTGGGAATCTGAAAGAAGAGAAGCAGGCCCATTTATATGGGAAAGAACAATA
GTATTTCTTATATAGGCCCATTTAAGTTGAAAACAATCTTCAAAAGTCCCACATCGCTTAGATAAGAAAACGAA
GCTGAGTTTATATACAGCTAGAGTCGAAGTAGTGATTGNNNNNNNNNNNNNNNNNNNGTT

Primers:

MT2-F: GGCACCAAGAAGCGNNNNNNNNNNNNNNNNNNNGTT

MT2-F0: TGNNNNNNNNNNNNNNNNNNNGTTTTAGAGCTAGAAATAGC

MT3-R0/R: AACNNNNNNNNNNNNNNNNNNNCAATCACTACTTCGACTCTAGC

MT3-BsR: ATTATTGGTCTCTAAACNNNNNNNNNNNNNNNNNNNC

Template: pCBC-MT2T3

Length: 887-bp (MT2T3-PCR) or 873-bp (MT2T3-PCR0)

Notes:

1. The 19-nt N in primers represent any 19-nt target sequence (forward primers) or reverse complement sequence of any 19-nt target sequence (reverse primers) in front of PAM (NGG).
2. For the assembly of three gRNA expression cassettes, use MT2-F/MT2-F0/MT3-R0/MT3-BsR four-primer mixture with MT2-F0/MT3-R0 diluted to 20 times of MT2-F or MT3-BsR, resulting in MT2T3-PCR.
3. For the assembly of more than three gRNA expression cassettes, use MT2-F/MT2-F0/MT3-R three-primer mixture with MT2-F0 diluted to 20 times of MT2-F or MT3-R, resulting in MT2T3-PCR0.

Sequence of MT3T4-PCR with Targets 3 and 4 for monocots

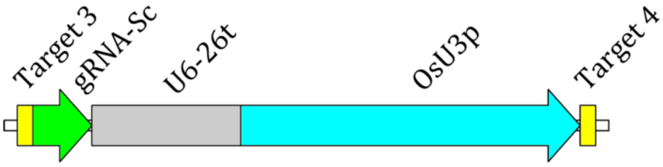

(Target-3)-(gRNA-Sc)-(U6-26t)-(OsU3p)-(Target-4)

GCTAGAGTCGAAGTAGTGATTGNNNNNNNNNNNNNNNNNNNGTTTTAGAGCTAGAAATAGCAAGTTAA
AATAAGGCTAGTCCGTTATCAACTTGAAAAAGTGGCACCGAGTCGGTGCTTTTTTTTGCAAAATTTTCCAGAT
CGATTTCTTCTTCCTCTGTTCTTCGGCGTTCAATTTCTGGGGTTTTCTCTTCGTTTTCTGTAACTGAAACCTAAA
ATTTGACCTAAAAAAAATCTCAAATAATATGATTCAGTGGTTTTGTACTTTTCAGTTAGTTGAGTTTTGCAGTT
CCGATGAGATAAACCAATAAGTAATTCATCCAGGTCACCAAGTTCTAGGATTTTCAGAACTGCAACTTATTTTA
TCAAGGAATCTTTAAACATACGAACAGATCACTTAAAGTTCTTCTGAAGCAACTTAAAGTTATCAGGCATGCA
TGGATCTTGGAGGAATCAGATGTGCAGTCAGGGACCATAGCACAAGACAGGCGTCTTCTACTGGTGCTACC
AGCAAATGCTGGAAGCCGGGAACACTGGGTACGTTGGAAACCACGTGATGTGAAGAAGTAAGATAAACTGT
AGGAGAAAAGCATTTCGTAGTGGGCCATGAAGCCTTTCAGGACATGTATTGCAGTATGGGCCGGCCCATTAC
GCAATTGGACGACAACAAAGACTAGTATTAGTACCACCTCGGCTATCCACATAGATCAAAGCTGATTTAAAAG
AGTTGTGCAGATGATCCGTGGCGNNNNNNNNNNNNNNNNNNNGTTTCGAGACCAATAAT

Primers:

MT3-F: GCTAGAGTCGAAGTAGTGATTGNNNNNNNNNNNNNNNNNNNGTT

MT3-F0: TGNNNNNNNNNNNNNNNNNNNGTTTTAGAGCTAGAAATAGC

MT4-R0: AACNNNNNNNNNNNNNNNNNNNCGCCACGGATCATCTGCACAACT 3'

MT4-BsR: ATTATTGGTCTCTAAACNNNNNNNNNNNNNNNNNNNC

Template: pCBC-MT3T4

Length: 783-bp

Notes:

1. The 19-nt N in primers represent any 19-nt target sequence (forward primers) or reverse complement sequence of any 19-nt target sequence (reverse primers) in front of PAM (NGG).
2. For the assembly of four gRNA expression cassettes, use MT3-F/MT3-F0/MT4-R0/MT4-BsR four-primer mixture with MT3-F0/MT4-R0 diluted to 20 times of MT3-F or MT4-BsR.
3. The *Bsa*I site on OsU3p has been mutated to prevent PCR products from being digested on undesirable site.

Sequence of MT1-T3-PCR with Targets 1, 2 and 3 for monocots

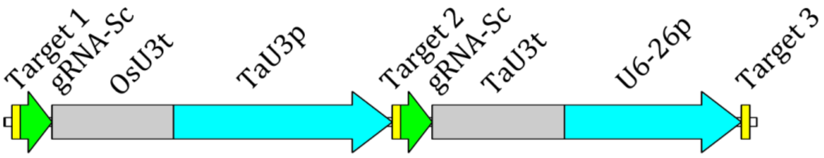

(Target-1)-(gRNA-Sc)-(OsU3t)-(TaU3p)-(Target-2)-(gRNA-Sc)-(TaU3t)-(U6-26p)-(Target-3)
GNNNNNNNNNNNNNNNNNNNGTTTTAGAGCTAGAAATAGCAAGTTAAAATAAGGCTAGTCCGTTATCAA
CTTGAAAAAGTGGCACCGAGTCGGTGCTTTTTTTTTTCGTTTTGCATTGAGTTTTCTCCGTCGCATGTTTGCA
GTTTTATTTTCCGTTTTGCATTGAAATTTCTCCGTCTCATGTTTGCAGCGTGTTCAAAAAGTACGCAGCTGTAT
TTCACTTATTTACGGCGCCACATTTTCATGCCGTTTGTGCCAACTATCCCGAGCTAGTGAATACAGCTTGGCTT
CACACAACACTGGTGACCCGCTGACCTGCTCGTACCTCGTACCGTCGTACGGCACAGCATTTGGAATTAAAG
GGTGTGATCGATACTGCTTGCTGCTCATGAATCCAAACCACACGGAGTTCAAATTCCCACAGATTAAGGCTC
GTCCGTCGCACAAGGTAATGTGTGAATATTATATCTGTCGTGCAAAATTGCCTGGCCTGCACAATTGCTGTTAT
AGTTGGCGGCAGGGAGAGTTTTAACATTGACTAGCGTGCTGATAATTTGTGAGAAATAATAATTGACAAGTA
GATACTGACATTTGAGAAGAGCTTCTGAACTGTTATTAGTAACAAAAATGGAAAGCTGATGCACGGAAAAAG
GAAAGAAAAAGCCATACTTTTTTTTAGGTAGGAAAAGAAAAAGCCATACGAGACTGATGTCTCTCAGATGG
GCCGGGATCTGTCTATCTAGCAGGCAGCAGCCCACCAACCTCACGGGCCAGCAATTACGAGTCCTTCTAAAA
GCTCCCGCCGAGGGGCGCTGGCGCTGCTGTGCAGCAGCACGTCTAACATTAGTCCCACCTCGCCAGTTTAC
AGGGAGCAGAACCAGCTTATAAGCGGAGGCGCGGCACCAAGAAGCGNNNNNNNNNNNNNNNNNNNG
TTTTAGAGCTAGAAATAGCAAGTTAAAATAAGGCTAGTCCGTTATCAACTTGAAAAAGTGGCACCGAGTCGG
TGCTTTTTTTTTTGTCCTTCTGTTTTTTTAGTCAGTCTCTTTTTTCAGAAGTACAACATCTTTTTTTTGTCCTTCT
GTTTTTTTAGTCAGTCTCTTTTTTCAGAAGTACAACATCTTTTTTTTGTCCTTCTGTTTTTTTAGTCAGTCTCTT
TTTTCAGAAGTACAACATCTTTTTTTTGTCCTTCTGTTTTTTTAGTCAGTCTCTTTTTTCAGAAGTACTCTATGA
TGTATCGTTCTGGGAAATGTCTGTCTGTCTACAACCCCATAATCTATATTTGCAATCACACATCTAATATTCTCTG
TGACAAGACAGCCGAACACGACTTGCCTTCCGCACAATACATCATTTCTTCTTAGCTTTTTTTCTTCTTCTTCG
TTCATACAGTTTTTTTTTGTTTATCAGCTTACATTTTCTTGAACCGTAGCTTTCGTTTTCTTCTTTTTAACTTTCC
ATTCGGAGTTTTTGTATCTTGTTTCATAGTTTGTCCCAGGATTAGAATGATTAGGCATCGAACCTTCAAGAATT
TGATTGAATAAAACATCTTCATTCTTAAGATATGAAGATAATCTTCAAAAGGCCCCTGGGAATCTGAAAGAAG
AGAAGCAGGCCCATTTATATGGGAAAGAACAATAGTATTTCTTATATAGGCCCATTTAAGTTGAAAACAATCTT
CAAAAGTCCCACATCGCTTAGATAAGAAAACGAAGCTGAGTTTATATACAGCTAGAGTCGAAGTAGTGATTG
NNNNNNNNNNNNNNNNNNN

Primers:

MT1-BsF: ATATATGGTCTCTGGCGNNNNNNNNNNNNNNNNNNNGTT

MT3-BsR: ATTATTGGTCTCTAAACNNNNNNNNNNNNNNNNNNNC

Template:

Gibson Assembly product of the MT1T2-PCR and MT2T3-PCR fragments was used as template for the second round of PCR amplification with MT1-BsF/MT3-BsR primers, resulting in MT1-T3-PCR.

Length: 1.8-kb

Sequence of MT1-T4-PCR with Targets 1, 2, 3 and 4 for monocots

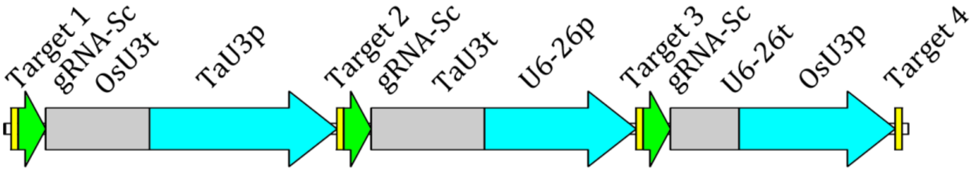

(Target-1)-(gRNA-Sc)-(OsU3t)-(TaU3p)-(Target-2)-(gRNA-Sc)-(TaU3t)-(U6-26p)-
(Target-3)-(gRNA-Sc)-(U6-26t)-(OsU3p)-(Target-4)

GNNNNNNNNNNNNNNNNNNNGTTTTAGAGCTAGAAATAGCAAGTTAAAATAAGGCTAGTCCGTTATCAACTTGAAAAAGTGGCACCGAGTCGGTGCT
TTTTTTTTTCGTTTTGCATTGAGTTTTCTCCGTCGCATGTTTGCAGTTTTATTTTCCGTTTTGCATTGAAATTTCTCCGTCTCATGTTTGCAGCGTGTTCAAAA
AGTACGCAGCTGTATTTCACTTATTTACGGCGCCACATTTTCATGCCGTTTGTGCCAACTATCCCGAGCTAGTGAATACAGCTTGGCTTCACACAACACTGGT
GACCCGCTGACCTGCTCGTACCTCGTACCGTCGTACGGCACAGCATTTGGAATTAAAGGGTGTGATCGATACTGCTTGCTGCTCATGAATCCAAACCACACG
GAGTTCAAATTCCCACAGATTAAGGCTCGTCCGTCGCACAAGGTAATGTGTGAATATTATATCTGTCGTGCAAAATTGCCTGGCCTGCACAATTGCTGTTATA
GTTGGCGGCAGGGAGAGTTTTAACATTGACTAGCGTGCTGATAATTTGTGAGAAATAATAATTGACAAGTAGATACTGACATTTGAGAAGAGCTTCTGAAC
TGTTATTAGTAACAAAAATGGAAAGCTGATGCACGGAAAAAGGAAAGAAAAAGCCATACTTTTTTTTAGGTAGGAAAAGAAAAAGCCATACGAGACTGAT
GTCTCTCAGATGGGCCGGGATCTGTCTATCTAGCAGGCAGCAGCCCACCAACCTCACGGGCCAGCAATTACGAGTCCTTCTAAAAGCTCCCGCCGAGGGG
CGCTGGCGCTGCTGTGCAGCAGCACGTCTAACATTAGTCCCACCTCGCCAGTTTACAGGGAGCAGAACCAGCTTATAAGCGGAGGCGCGGCACCAAGAA
GCGNNNNNNNNNNNNNNNNNNNGTTTTAGAGCTAGAAATAGCAAGTTAAAATAAGGCTAGTCCGTTATCAACTTGAAAAAGTGGCACCGAGTCGGTG
CTTTTTTTTTTGTCCTTCTGTTTTTTTAGTCAGTCTCTTTTTTCAGAAGTACAACATCTTTTTTTTGTCCTTCTGTTTTTTTAGTCAGTCTCTTTTTTCAGAAGTA
CAACATCTTTTTTTTGTCCTTCTGTTTTTTTAGTCAGTCTCTTTTTTCAGAAGTACAACATCTTTTTTTTGTCCTTCTGTTTTTTTAGTCAGTCTCTTTTTTCAGA
AGTACTCTATGATGTATCGTTCTGGGAAATGTCTGTCTGTCTACAACCCCATAATCTATATTTGCAATCACACATCTAATATTCTCTGTGACAAGACAGCCGAAC
ACGACTTGCCTTCCGCACAATACATCATTTCTTCTTAGCTTTTTTTCTTCTTCTTCGTTCATACAGTTTTTTTTTGTTTATCAGCTTACATTTTCTTGAACCGTAG
CTTTCGTTTTCTTCTTTTTAACTTTCCATTCGGAGTTTTTGTATCTTGTTTCATAGTTTGTCCCAGGATTAGAATGATTAGGCATCGAACCTTCAAGAATTTGAT
TGAATAAAACATCTTCATTCTTAAGATATGAAGATAATCTTCAAAAGGCCCCTGGGAATCTGAAAGAAGAGAAGCAGGCCCATTTATATGGGAAAGAACAAT
AGTATTTCTTATATAGGCCCATTTAAGTTGAAAACAATCTTCAAAAGTCCCACATCGCTTAGATAAGAAAACGAAGCTGAGTTTATATACAGCTAGAGTCGAA
GTAGTGATTGNNNNNNNNNNNNNNNNNNNGTTTTAGAGCTAGAAATAGCAAGTTAAAATAAGGCTAGTCCGTTATCAACTTGAAAAAGTGGCACCGA
GTCGGTGCTTTTTTTTGCAAAATTTTCCAGATCGATTTCTTCTTCCTCTGTTCTTCGGCGTTCAATTTCTGGGGTTTTCTCTTCGTTTTCTGTAACTGAAACCT
AAAATTTGACCTAAAAAAAAATCTCAAATAATATGATTCAGTGGTTTTGTACTTTTCAGTTAGTTGAGTTTTGCAGTTCCGATGAGATAAACCAATAAGTAATTC
ATCCAGGTCACCAAGTTCTAGGATTTTCAGAACTGCAACTTATTTTATCAAGGAATCTTTAAACATACGAACAGATCACTTAAAGTTCTTCTGAAGCAACTTA
AAGTTATCAGGCATGCATGGATCTTGGAGGAATCAGATGTGCAGTCAGGGACCATAGCACAAGACAGGCGTCTTCTACTGGTGCTACCAGCAAATGCTGG
AAGCCGGGAACACTGGGTACGTTGGAAACCACGTGATGTGAAGAAGTAAGATAAACTGTAGGAGAAAAGCATTTCGTAGTGGGCCATGAAGCCTTTCAG
GACATGTATTGCAGTATGGGCCGGCCCATTACGCAATTGGACGACAACAAAGACTAGTATTAGTACCACCTCGGCTATCCACATAGATCAAAGCTGATTTAA
AAGAGTTGTGCAGATGATCCGTGGCGNNNNNNNNNNNNNNNNNNN

Primers:

MT1-BsF: ATATATGGTCTCTGGCGNNNNNNNNNNNNNNNNNNNGTT

MT4-BsR: ATTATTGGTCTCTAAACNNNNNNNNNNNNNNNNNNNC

Template:

Gibson Assembly product of the MT1T2-PCR, MT2T3-PCR, and MT3T4-PCR fragments was used as template for the second round of PCR amplification with MT1-BsF/MT4-BsR primers, resulting in MT1-T4-PCR.

Length: 2.5-kb

Sequence of three gRNA expression cassettes for monocots

MT1-T3-PCR + pBUN411 et al.

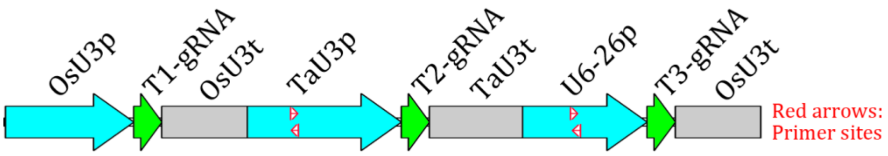

(OsU3p)-(Target-1)-(gRNA-Sc)-(OsU3t)-(TaU3p)-(Target-2)-(gRNA-Sc)-(TaU3t)-
(U6-26p)-(Target-3)-(gRNA-Sc)-(OsU3t)

AGTAATTCATCCAGGTCTCCAAGTTCTAGGATTTTCAGAACTGCAACTTATTTTATCAAGGAATCTTTAAACATACGAACAGATCA
CTTAAAGTTCTTCTGAAGCAACTTAAAGTTATCAGGCATGCATGGATCTTGGAGGAATCAGATGTGCAGTCAGGGACCATAGCA
CAAGACAGGCGTCTTCTACTGGTGCTACCAGCAAATGCTGGAAGCCGGGAACACTGGGTACGTTGGAAACCACGTGATGTGA
AGAAGTAAGATAAACTGTAGGAGAAAAGCATTTCGTAGTGGGCCATGAAGCCTTTCAGGACATGTATTGCAGTATGGGCCGGC
CCATTACGCAATTGGACGACAACAAAGACTAGTATTAGTACCACCTCGGCTATCCACATAGATCAAAGCTGATTTAAAAGAGTTG
TGCAGATGATCCGTGGCGNNNNNNNNNNNNNNNNNNNGTTTTAGAGCTAGAAATAGCAAGTTAAAATAAGGCTAGTCCGTT
ATCAACTTGAAAAAGTGGCACCGAGTCGGTGCTTTTTTTTTTCGTTTTGCATTGAGTTTTCTCCGTCGCATGTTTGCAGTTTTATT
TTCCGTTTTGCATTGAAATTTCTCCGTCTCATGTTTGCAGCGTGTTCAAAAAGTACGCAGCTGTATTTCACTTATTTACGGCGCCA
CATTTTCATGCCGTTTGTGCCAACTATCCCGAGCTAGTGAATACAGCTTGGCTTCACACAACACTGGTGACCCGCTGACCTGCTC
GTACCTCGTACCGTCGTACGGCACAGCATTTGGAATTAAAGGGTGTGATCGATACTGCTTGCTGCTCATGAATCCAAACCACACG
GAGTTCAAATTCCCACAGATTAAGGCTCGTCCGTCGCACAAGGTAATGTGTGAATATTATATCTGTCGTGCAAAATTGCCTGGCCT
GCACAATTGCTGTTATAGTTGGCGGCAGGGAGAGTTTTAACATTGACTAGCGTGCTGATAATTTGTGAGAAATAATAATTGACA
AGTAGATACTGACATTTGAGAAGAGCTTCTGAACTGTTATTAGTAACAAAAATGGAAAGCTGATGCACGGAAAAAGGAAAGAA
AAAGCCATACTTTTTTTTAGGTAGGAAAAGAAAAAGCCATACGAGACTGATGTCTCTCAGATGGGCCGGGATCTGTCTATCTAG
CAGGCAGCAGCCCACCAACCTCACGGGCCAGCAATTACGAGTCCTTCTAAAAGCTCCCGCCGAGGGGCGCTGGCGCTGCTGT
GCAGCAGCACGTCTAACATTAGTCCCACCTCGCCAGTTTACAGGGAGCAGAACCAGCTTATAAGCGGAGGCGCGGCACCAAG
AAGCGNNNNNNNNNNNNNNNNNNNGTTTTAGAGCTAGAAATAGCAAGTTAAAATAAGGCTAGTCCGTTATCAACTTGAAAA
AGTGGCACCGAGTCGGTGCTTTTTTTTTTGTCCTTCTGTTTTTTTAGTCAGTCTCTTTTTTCAGAAGTACAACATCTTTTTTTTGTC
CTTCTGTTTTTTTAGTCAGTCTCTTTTTTCAGAAGTACAACATCTTTTTTTTGTCCTTCTGTTTTTTTAGTCAGTCTCTTTTTTCAGA
AGTACAACATCTTTTTTTTGTCCTTCTGTTTTTTTAGTCAGTCTCTTTTTTCAGAAGTACTCTATGATGTATCGTTCTGGGAAATGTC
TGTCTGTCTACAACCCCATAATCTATATTTGCAATCACACATCTAATATTCTCTGTGACAAGACAGCCGAACACGACTTGCCTTCCG
CACAATACATCATTTCTTCTTAGCTTTTTTTCTTCTTCTTCGTTCATACAGTTTTTTTTTGTTTATCAGCTTACATTTTCTTGAACCGT
AGCTTTCGTTTTCTTCTTTTTAACTTTCCATTCGGAGTTTTTGTATCTTGTTTCATAGTTTGTCCCAGGATTAGAATGATTAGGCAT
CGAACCTTCAAGAATTTGATTGAATAAAACATCTTCATTCTTAAGATATGAAGATAATCTTCAAAAGGCCCCTGGGAATCTGAAAG
AAGAGAAGCAGGCCCATTTATATGGGAAAGAACAATAGTATTTCTTATATAGGCCCATTTAAGTTGAAAACAATCTTCAAAAGTC
CCACATCGCTTAGATAAGAAAACGAAGCTGAGTTTATATACAGCTAGAGTCGAAGTAGTGATTGNNNNNNNNNNNNNNNNN
NNGTTTTAGAGCTAGAAATAGCAAGTTAAAATAAGGCTAGTCCGTTATCAACTTGAAAAAGTGGCACCGAGTCGGTGCTTTTTT
TTTTCGTTTTGCATTGAGTTTTCTCCGTCGCATGTTTGCAGTTTTATTTTCCGTTTTGCATTGAAATTTCTCCGTCTCATGTTTGCA
GCGTGTTCAAAAAGTACGCAGCTGTATTTCACTTATTTACGGCGCCACATTTTCATGCCGTTTGTGCCAACTATCCCGAGCTAGT
GAATACAGCTTGGCTTCACACAACACTGGTGACCCGCTGACCTGCTCGTACCTCGTACCGTCGTACGGCACAGCATTTGGAATT
AAAGGGTGTGATCGATACTGCTTGCTGCT

Notes:

1. Underlined letters come from binary vectors, while the others come from PCR fragments.
2. Red letters indicate primer sites.
3. Primer sequences are as follows:

Colony PCR primers (5'→3'):

TaU3p-F2: TTGACTAGCGTGCTGATAATTTGTG

U6-26p-R2: *AGGTTCGAT*GCCTAATCATTCTAATCCT

(TaU3p-F2 + U6-26p-R2 = 987 bp)

Sequencing primers (5'→3'):

TaU3p-F2: TTGACTAGCGTGCTGATAATTTGTG

TaU3p-R: *CTCACAAATTATCAGCACGCTAGTC*

U6-26p-F: TGTCCCAGGATTAGAATGATTAGGC

Sequence of four gRNA expression cassettes for monocots

MT1-T4-PCR + pBUN411 et al.

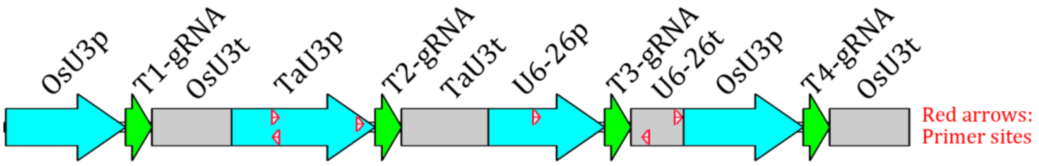

(OsU3p)-(Target-1)-(gRNA-Sc)-(OsU3t)-(TaU3p)-(Target-2)-(gRNA-Sc)-(TaU3t)-
(U6-26p)-(Target-3)-(gRNA-Sc)-(U6-26t)-(OsU3p)-(Target-4)-(gRNA-Sc)-(OsU3t)

AGTAATTCATCCAGGTCTCCAAGTTCTAGGATTTTCAGAACTGCAACTTATTTTATCAAGGAATCTTTAAACATACGAACAGATCACTTAAAGTTCTTCTGAA
GCAACTTAAAGTTATCAGGCATGCATGGATCTTGGAGGAATCAGATGTGCAGTCAGGGACCATAGCACAAGACAGGCGTCTTCTACTGGTGCTACCAGCA
AATGCTGGAAGCCGGGAACACTGGGTACGTTGGAAACCACGTGATGTGAAGAAGTAAGATAAACTGTAGGAGAAAAGCATTTCGTAGTGGGCCATGAAG
CCTTTCAGGACATGTATTGCAGTATGGGCCGGCCCATTACGCAATTGGACGACAACAAAGACTAGTATTAGTACCACCTCGGCTATCCACATAGATCAAAGC
TGATTTAAAAGAGTTGTGCAGATGATCCGTGGCGNNNNNNNNNNNNNNNNNNNGTTTTAGAGCTAGAAATAGCAAGTTAAAATAAGGCTAGTCCGTTA
TCAACTTGAAAAAGTGGCACCGAGTCGGTGCTTTTTTTTTTCGTTTTGCATTGAGTTTTCTCCGTCGCATGTTTGCAGTTTTATTTTCCGTTTTGCATTGAAA
TTTCTCCGTCTCATGTTTGCAGCGTGTTCAAAAAGTACGCAGCTGTATTTCACTTATTTACGGCGCCACATTTTCATGCCGTTTGTGCCAACTATCCCGAGCT
AGTGAATACAGCTTGGCTTCACACAACACTGGTGACCCGCTGACCTGCTCGTACCTCGTACCGTCGTACGGCACAGCATTTGGAATTAAAGGGTGTGATCG
ATACTGCTTGCTGCTCATGAATCCAAACCACACGGAGTTCAAATTCCCACAGATTAAGGCTCGTCCGTCGCACAAGGTAATGTGTGAATATTATATCTGTCGT
GCAAAATTGCCTGGCCTGCACAATTGCTGTTATAGTTGGCGGCAGGGAGAGTTTTAACA**TT*GACTAGCGTGCTGATAATTTGTGAG***AAATAATAATTGACA
AGTAGATACTGACATTTGAGAAGAGCTTCTGAACTGTTATTAGTAACAAAAATGGAAAGCTGATGCACGGAAAAAGGAAAGAAAAAGCCATACTTTTTTT
AGGTAGGAAAAGAAAAAGCCATACGAGACTGATGTCTCTCAGATGGGCCGGGATCTGTCTATCTAGCAGGCAGCAGCCCACCAACCTCACGGGCCAGCA
ATTACGAGTCCTTCTAAAAGCTCCCGCCGAGGGGCGCTGGCGCTGCTGTGCAGCAGCACGTCTAACA**TTAGTCCCACCTCGCCAGTTTACAG**GGAGCAG
AACCAGCTTATAAGCGGAGGCGCGGCACCAAGAAGCGNNNNNNNNNNNNNNNNNNNGTTTTAGAGCTAGAAATAGCAAGTTAAAATAAGGCTAGTC
CGTTATCAACTTGAAAAAGTGGCACCGAGTCGGTGCTTTTTTTTTTGTCCTTCTGTTTTTTTAGTCAGTCTCTTTTTTCAGAAGTACAACATCTTTTTTTTGTC
CTTCTGTTTTTTTAGTCAGTCTCTTTTTTCAGAAGTACAACATCTTTTTTTTGTCCTTCTGTTTTTTTAGTCAGTCTCTTTTTTCAGAAGTACAACATCTTTTTT
TTGTCCTTCTGTTTTTTTAGTCAGTCTCTTTTTTCAGAAGTACTCTATGATGTATCGTTCTGGGAAATGTCTGTCTGTCTACAACCCCATAATCTATATTTGCAAT
CACACATCTAATATTCTCTGTGACAAGACAGCCGAACACGACTTGCCTTCCGCACAATACATCATTTCTTCTTAGCTTTTTTTCTTCTTCTTCGTTCATACAGTT
TTTTTTTGTTTATCAGCTTACATTTTCTTGAACCGTAGCTTTCGTTTTCTTCTTTTTAACTTTCCATTCGGAGTTTTTGTATCTTGTTTCATAGTT**TGTCCCAGGA
TTAGAATGATTAGGC**ATCGAACCTTCAAGAATTTGATTGAATAAAACATCTTCATTCTTAAGATATGAAGATAATCTTCAAAAGGCCCCTGGGAATCTGAAAG
AAGAGAAGCAGGCCCATTTATATGGGAAAGAACAATAGTATTTCTTATATAGGCCCATTTAAGTTGAAAACAATCTTCAAAAGTCCCACATCGCTTAGATAAG
AAAACGAAGCTGAGTTTATATACAGCTAGAGTCGAAGTAGTGATTGNNNNNNNNNNNNNNNNNNNGTTTTAGAGCTAGAAATAGCAAGTTAAAATAAG
GCTAGTCCGTTATCAACTTGAAAAAGTGGCACCGAGTCGGTGCTTTTTTTTGCAAAATTTTCCAGATCGATTTCTTCTTCCTCT**GTTCTTCGGCGTTCAATTT
CTGGGG**TTTTCTCTTCGTTTTCTGTAACTGAAACCTAAAATTTGACCTAAAAAAAATCTCAAATAATATGATTCAGTGGTTTTGTACTTTTCAGTTAGTT**GAGT
TTTGCAGTTCCGATGAGATAAACC**AATAAGTAATTCATCCAGGTCACCAAGTTCTAGGATTTTCAGAACTGCAACTTATTTTATCAAGGAATCTTTAAACATA
CGAACAGATCACTTAAAGTTCTTCTGAAGCAACTTAAAGTTATCAGGCATGCATGGATCTTGGAGGAATCAGATGTGCAGTCAGGGACCATAGCACAAGAC
AGGCGTCTTCTACTGGTGCTACCAGCAAATGCTGGAAGCCGGGAACACTGGGTACGTTGGAAACCACGTGATGTGAAGAAGTAAGATAAACTGTAGGAG
AAAAGCATTTCGTAGTGGGCCATGAAGCCTTTCAGGACATGTATTGCAGTATGGGCCGGCCCATTACGCAATTGGACGACAACAAAGACTAGTATTAGTAC
CACCTCGGCTATCCACATAGATCAAAGCTGATTTAAAAGAGTTGTGCAGATGATCCGTGGCGNNNNNNNNNNNNNNNNNNNGTTTTAGAGCTAGAAAT
AGCAAGTTAAAATAAGGCTAGTCCGTTATCAACTTGAAAAAGTGGCACCGAGTCGGTGCTTTTTTTTTTCGTTTTGCATTGAGTTTTCTCCGTCGCATGTTT
GCAGTTTTATTTTCCGTTTTGCATTGAAATTTCTCCGTCTCATGTTTGCAGCGTGTTCAAAAAGTACGCAGCTGTATTTCACTTATTTACGGCGCCACATTTTC
ATGCCGTTTGTGCCAACTATCCCGAGCTAGTGAATACAGCTTGGCTTCACACAACACTGGTGACCCGCTGACCTGCTCGTACCTCGTACCGTCGTACGGCAC
AGCATTTGGAATTAAAGGGTGTGATCGATACTGCTTGCTGCT

Notes:

1. Underlined letters come from binary vectors, while the others come from PCR fragments.
2. Red letters indicate primer sites.
3. Primer sequences are as follows:

Colony PCR primers (5'→3'):

TaU3p-F: TTAGTCCCACCTCGCCAGTTTACAG

U6-26t-R: CCCCAGAAATTGAACGCCGAAGAAC

(TaU3p-F + U6-26t-R = 1.2 kb)

Sequencing primers (5'→3'):

TaU3p-F2: TT*GACTAGCGTGCTGATAATTTGTG*

TaU3p-R: *CTCACAAATTATCAGCACGCTAGTC*

U6-26p-F: TGTCCCAGGATTAGAATGATTAGGC

U6-26t-F2: GAGTTTTGCAGTTCCGATGAGATAAACC
